# AMDHD1 acts as a tumor suppressor and contributes to activation of TGF-β signaling pathway in cholangiocarcinoma

**DOI:** 10.1038/s41418-024-01361-y

**Published:** 2024-08-14

**Authors:** Zuyi Ma, Jia Sun, Zhenchong Li, Shanzhou Huang, Binglu Li

**Affiliations:** 1 Department of General Surgery, State Key Laboratory of Complex Severe and Rare Diseases, Peking Union Medical College Hospital, Chinese Academy of Medical Science and Peking Union Medical College, Beijing, China; 2 Junior Clinical Cooperation Unit Translational Gastrointestinal Oncology and Preclinical Models, German Cancer Research Center (DKFZ), Heidelberg, Germany; 3 Department of General Surgery, Guangdong Provincial People’s Hospital (Guangdong Academy of Medical Sciences), Southern Medical University, Guangzhou, China; 4 Southern Medical University, Guangzhou, China

**Keywords:** Tumour-suppressor proteins, Ubiquitylation, Tumour biomarkers

## Abstract

Cholangiocarcinoma (CCA) is a malignant tumor of the digestive system, characterized by its aggressive behavior and the absence of effective therapeutic biomarkers. Although recent studies have implicated AMDHD1 in tumor formation, its role in CCA development has been insufficiently explored. We utilized multiple bioinformatic datasets alongside 108 clinical samples to examine AMDHD1 expression in CCA. Then, in vitro and in vivo experiments were conducted to assess its impact on tumor growth and metastasis. Furthermore, proteomic analysis and immunoprecipitation mass spectrometry were employed to identify the downstream effectors of AMDHD1. We discovered that AMDHD1 was down-regulated in CCA and this down-regulation was associated with adverse clinicopathological features and prognosis. We also demonstrated that overexpression of AMDHD1 hindered G1/S progression in the cell cycle and promoted apoptosis, thereby inhibiting tumor growth and metastasis. Mechanistically, we found that AMDHD1 operated in a TGF-β-dependent manner and the inhibition of TGF-β signaling abrogated the effect of AMDHD1 overexpression on CCA cells. Specifically, AMDHD1 inhibited the ubiquitination and degradation of the SMAD4 protein through binding to the MH2 domain and synergistically enhanced SMAD2/3 phosphorylation, which activated of TGF-β signaling pathway and resulted in the suppression of CCA cell proliferation and migration. Our study identifies AMDHD1 as a significant prognostic biomarker and a tumor suppressor in CCA. It underscores the pivotal role of the AMDHD1/TGF-β signaling pathway in the development and progression of CCA.

## Introduction

Cholangiocarcinoma (CCA), originating from the epithelial cells of the bile duct, ranks as the second most common primary hepatobiliary malignancy. Based on anatomical distinctions, CCA is categorized into intrahepatic CCA (iCCA), perihilar CCA (pCCA) and distal CCA (dCCA) subtypes. The incidence of CCA is notably high in Southeast Asia, particularly in China and Thailand, with an average annual increase of 4.4%, primarily attributed to the rising cases of iCCA, while the incidences of pCCA and dCCA are declining or remain stable [1]. CCA represents one of the deadliest digestive tumors, exhibiting a 5-year overall survival (OS) rate of only 7% to 20% [2]. Radical operation offers the only potential cure for patients diagnosed in the early stages; however, options for unresectable or metastatic cases remain severely limited. The combination of gemcitabine and cisplatin, as established by the ABC-02 trial, has been the standard first-line systemic treatment for advanced CCA [3]. In recent years, several innovative therapies, including pemigatinib, durvalumab, infgratinib and ivosidenib, have been approved to enhance patient outcomes [4]. Despite these advances, the long-term survival of CCA patients is still disappointing, largely due to the disease’s aggressive nature and the propensity for early tumor recurrence. Therefore, identifying novel biomarkers for early diagnosis and exploring the molecular mechanisms underlying carcinogenesis and progression in CCA are of significant importance.

Amidohydrolase Domain Containing 1 (AMDHD1), also known as HMFT1272, is localized to chromosome 12q23.1 and encodes an enzyme which is pivotal in the metabolism pathways of histidine, phenylalanine, tyrosine, tryptophan, and vitamin D [5, 6]. AMDHD1 has been identified as a liver-specific gene, under negative regulation by HNF4α and miR-122, playing significant roles in liver development and hepatogenesis [7]. Recent research has begun to elucidate its involvement in tumorigenesis and tumor progression. Notably, downregulation of AMDHD1 has been observed in hepatocellular carcinoma and adrenal cancer through transcriptomic and proteomic analyses [6, 8]. Furthermore, single nucleotide polymorphisms, specifically rs11826 C > T, within AMDHD1 have been implicated in the up-regulation of its expression, providing a protective effect against the risk of breast cancer [9]. Despite these insights, the biological role and molecular mechanisms of AMDHD1 in CCA remain largely uncharted territories. In the current study, we demonstrated that AMDHD1 interacted with SMAD4 and SMAD2/3, which activated Transforming Growth Factor β (TGF-β) signaling pathway and subsequently suppressed the malignant traits of CCA cells, revealing a novel aspect of AMDHD1’s role in cancer biology and offering potential new avenues for therapeutic intervention.

The TGF-β signaling pathway is instrumental in regulating a variety of cellular biological processes, including cell proliferation, migration, apoptosis, differentiation, as well as tumor initiation and progression [10]. SMAD proteins act as pivotal signal transduction molecules within the TGF-β pathway, conveying extracellular signals directly to the nucleus. Activation of TGF-β signaling cascade begins when TGF-β binds to its two transmembrane receptors, TGF-βRI and TGF-βRII, which triggers the phosphorylation of SMAD2/3 and prompts their association with SMAD4. This newly formed heterodimeric SMAD complex then migrates to the nucleus and regulate target genes transcription, influencing a range of biological processes and leading to cell state-specific transcriptional regulation [11]. The role of TGF-β/SMAD signaling in tumorigenesis is paradoxical, as it can be both tumor-suppressive and oncogenic, depending on the type and stage of the cancer. During the early stages of tumor development, TGF-β signaling acts as a suppressor by blocking cell cycle and inducing apoptosis. However, as the tumor advances, cancer cells increasingly ignore the suppressive signals and begin to secrete TGF-β proteins, thereby promoting tumor angiogenesis, metastasis and immune evasion [12]. Deciphering the conditions and mechanisms that drive the shift of TGF-β signaling from a tumor suppressor to an oncogenic factor remains a significant and enduring challenge in cancer biology.

In our study, we utilized multiple bioinformatic datasets and analyzed 108 clinical samples to assess the expression of AMDHD1 in CCA, examining its correlation with clinicopathological characteristics and patient survival. Furthermore, we explored the inhibitory function of AMDHD1 on CCA cells proliferation and migration in vivo and in vitro. Proteomic analysis revealed that AMDHD1 mitigated the malignancy of CCA cells through a TGF-β-dependent mechanism. Importantly, we identified SMAD4 as the pivotal mediator in the AMDHD1-induced activation of TGF-β signaling. AMDHD1 bound to the MH2 functional domain of SMAD4, preventing the ubiquitin-mediated degradation of the SMAD4 protein and thereby enhancing its stability. Additionally, AMDHD1 was found to facilitate the phosphorylation of SMAD2/3, further amplifying TGF-β signaling through its interactions with these molecules. Our findings underscore the crucial involvement of AMDHD1 in TGF-β signaling pathway during tumorigenesis, positioning AMDHD1 as a potential novel therapeutic biomarker for CCA.

## Methods

### Bioinformatic data acquisition and analysis

The Gene Expression Profiling Interactive Analysis (GEPIA), which encompasses RNA sequencing expression data of 9736 tumors and 8587 normal samples from both the GTEx and TCGA projects, was employed to evaluate the transcriptional levels of AMDHD1 across various cancers, with a particular focus on CCA [13]. To corroborate AMDHD1 expression in CCA, we utilized datasets from the Gene Expression Omnibus (GEO), including GSE26566 (featuring 6 normal bile duct and 104 CCA samples), GSE32225 (comprising 6 normal bile duct and 149 CCA samples), GSE32958 (containing 7 normal bile duct and 16 CCA samples) and GSE76311 (incorporating 93 normal bile duct and 92 CCA samples). Furthermore, AMDHD1-associated co-expressed genes were screened out through cBioPortal database, adhering to stringent criteria (|Spearman’s correlation coefficient | >0.5 and *P* < 0.05) [14]. AMDHD1-interacting proteins were determined by Tandem Mass Tag (TMT) proteomic analysis. These genes and proteins were then integrated for functional enrichment analyses using the Gene Ontology (GO), Kyoto Encyclopedia of Genes and Genomes (KEGG) and Gene Set Enrichment Analysis (GSEA) methodologies as previously described [15]. Additionally, the STRING platform was used to establish protein-protein interaction networks, enabling the identification of proteins linked with TGF-β signaling pathway [16].

### CCA specimen acquisition and patient follow-up

Normal bile duct and CCA tissues were collected from 108 patients who underwent surgical resection and were diagnosed with CCA at Peking Union Medical College Hospital (PUMCH) and Guangdong Provincial People’s Hospital (GPPH) from January 2020 to December 2022. Follow-up continued until December 2023 and the median duration was 21.3 months (11.2–48 months). We compiled clinicopathological data, including age, gender, differentiation, perineural invasion, lymphnode metastasis, carbohydrate antigen 19-9 (CA19-9) levels and AJCC 8th edition TNM stage for analysis. Kaplan–Meier survival analyses were employed to evaluate the prognostic significance of AMDHD1 levels in CCA patients. The independent prognostic factors for CCA survivals were identified by Cox regression analyses. The research was carried out in strict accordance with the Declaration of Helsinki and approved by the Ethics Committees of PUMCH and GPPH, with the approval number I-23PJ274.

### Materials and cell transfection

The reagents and antibodies utilized in this research are listed in Table S1. RBE, QBC939, HUCCT1, CCLP-1, SNU-869 and HCCC-9810 cell lines were sourced from Procell (Wuhan, China) with STR identification reports and tested to exclude mycoplasma contamination. Cells were cultured in RPMI-1640 medium supplemented with 10% FBS (Servicebio, China) at 37 °C in 5% CO_2_ atmosphere. For AMDHD1 overexpression (AMDHD-OV) or knockdown (AMDHD1-KD), the vectors Puro-CMV-AMDHD1-3×FLAG and Puro-CMV-shAMDHD1-EGFP (OBiO Technology, China) were transfected into HEK293T cells. Three days post transfection, viral particles were harvested using concentrated virus precipitation solution from letinous edodes according to the protocols provided by System Biosciences, and TUNDUX virus transducers were utilized for infecting CCA cells. Subsequently, positively transduced cells were selected using puromycin. The processes for overexpressing and knocking down SMAD4 were identical to those described above. Table S2 details the sequences of shRNA oligonucleotides.

### In vitro functional experiments

Cell proliferation assays (colony formation and CCK-8 assays), migration assays (Transwell and wound healing assays) and flow cytometry to evaluate cell cycle and apoptosis was completed following previously established protocols [15].

### Real-time quantitative polymerase chain reaction (RT-qPCR) and western blot

The mRNA levels of target genes were assessed by RT-qPCR. Total RNA was extracted utilizing Invitrogen TRIzol (Thermo Fisher Scientific, USA) and converted to cDNA employing a reverse transcription kit. The RT-qPCR was completed utilizing the SYBR Green mix (Servicebio, China) and specific primers (Table S2). Relative mRNA expression was quantified by 2^−∆∆Ct^ method. To analyze the proteins, BCA Protein Quantitation Assay was used to determine protein concentration in cells and tissues lysed with RIPA buffer. Proteins were separated by 10% SDS-PAGE and transferred to nitrocellulose membranes. After blocking with BSA solution (Servicebio, China) for 15 min, the membranes were incubated with specific primary antibodies overnight. Secondary antibodies were applied before the final detection of protein bands using the Amersham Imager 680 system (Cytiva, USA).

### Immunohistochemistry (IHC) and immunofuorescence (IF) analysis

Paraffin-embedded human CCA tissues and mouse xenograft samples were subjected to IHC staining. Following deparaffinization and blocking, the samples were incubated with specific primary antibodies. IHC staining results were captured photographically and independently assessed by two pathologists. The H-scores were automatically generated by AIPATHWELL software (Servicebio, China), a digital pathological image analysis software based on artificial intelligence deep learning. To categorize high- and low-AMDHD1 groups, the X-tile software was adopted to identify the optimal cut-off value of the H-scores [17]. For IF analysis, HUCCT1 cells were treated with designated primary antibodies at 4 °C overnight, followed by incubation with the appropriate secondary antibodies at 37 °C for 2 h in a dark environment. Subsequently, nuclei were visualized by staining cells with DAPI for 10 min. Fluorescence imaging was conducted by confocal microscopy (Nikon A1R, Japan) in the dark setting.

### TMT proteomic analysis

Total proteins were extracted from AMDHD1-OV and control HUCCT1 cells and the concentrations were measured using the BCA Protein Quantitation Assay. Then, 20 μg of each protein sample was subjected to 12% SDS-PAGE and visualized using Coomassie Brilliant Blue R-250 staining. The proteins were then extracted from the gel, digested overnight with trypsin and CaCl_2_, and labeled with TMT reagents. Labeled samples were combined in equal volumes, desalted and lyophilized. Fractionated peptides were analyzed by shotgun proteomics using the EASY-nLC™ 1200 UHPLC system and Q Exactive™ HF-X mass spectrometer (both from Thermo Fisher Scientific). The resulting spectra were processed using Proteome Discoverer 2.2 software (AB Sciex, USA). Search parameters included a precursor ion mass tolerance of 10 ppm and a product ion mass tolerance of 0.02 Da. Fixed modifications included carbamidomethyl, while dynamic modifications included oxidation of methionine and TMT labeling. N-terminal modifications included acetylation and TMT labeling. A maximum of two miscleavage sites were permitted. Peptide Spectrum Matches (PSMs) were filtered to ensure a confidence level greater than 99%, and only proteins identified with at least one unique peptide were considered. PSMs and proteins were retained for further analysis with an FDR of no more than 1.0%. Differentially expressed proteins were identified based on a |log_2_Fold Change | > 0.5 and *P* < 0.05. Data from mass spectrometry proteomics has been deposited in the ProteomeXchange Consortium’s iProX partner repository as dataset IPX0008308000 [18].

### Co-immunoprecipitation (CO-IP) and mass spectrum (MS) analysis

For transfection-based CO-IP assays, we transfected plasmids overexpressing MYC-, Flag-, or HA-tagged proteins into HEK293T cells using Lipofectamine 3000. After 36 h post transfection, proteins were extracted from the cells and incubated with corresponding tag-specific antibodies and equal amounts of protein A/G beads overnight at 4 °C. Following three washes to eliminate non-specific binding, BCA method was used to quantify protein concentrations. Proteins were then eluted and subjected to western blot analysis. To investigate interactions between endogenous proteins, extracts from CCA cells were immunoprecipitated with specific primary antibodies (or IgG as a control) at 4 °C overnight. The immunoprecipitated complexes were thoroughly washed and prepared in SDS sample buffer for subsequent western blot analysis. MS analysis was employed to identify proteins that bind to AMDHD1. Bands of interest were excised from gels stained with Coomassie Blue, digested and analyzed by the Q Exactive™ HF-X mass spectrometer. Data quality control and analysis were carried out using Proteome Discoverer 2.2 software.

### Ubiquitination assay

Ubiquitin and substrate plasmids were transiently co-transfected into AMDHD1-OV, AMDHD1-KD and control cells. Twenty-four hours post transfection, cells were treated with the proteasome inhibitor MG132 for 6 h. Subsequently, cells were lysed using RIPA buffer and harvested. The subsequent specific procedures were outlined in the CO-IP section. SMAD4 ubiquitination was measured by immunoprecipitation with the antibody against SMAD4, followed by immunoblotting with the antibody against ubiquitin.

### Animal experiments

Animal experiments were performed with the approval and under the supervision of the Animal Welfare & Ethics Committee of PUMCH, with the ethical approval number XHDW-2024-32. Eight-week-old male Balb/c nude mice purchased from OBiO Technology were randomized into different groups. There were at least 6 mice in each group (*n* = 6). Xenograft models were established by subcutaneously injecting 1 × 10^6^ HUCCT1 cells into the BALB/c nude mice. Subcutaneous tumor sizes were measured and recorded every seven days. After six weeks post implantation, the mice were sacrificed and xenografts were excised, weighed and processed for IHC analysis using specific antibodies. Lung metastasis models were generated by intravenously injecting 2 × 10^5^ HUCCT1 cells labeled with luciferase into the BALB/c nude mice. Bioluminescence imaging was conducted every other day using the NightOWL LB 983 system (Berthold, Germany) to monitor metastasis. At the end of eight weeks, the mice were euthanized and their lungs were harvested, imaged and stained with Hematoxylin and eosin (H&E) for the evaluation of metastatic lesions.

### Statistical analysis

The investigators were aware of the group allocation during the study including animal experiments. Student’s *t* tests (unpaired two-tailed) and ANOVA were used to compare continuous variables between two groups and among multiple groups, respectively. Qualitative variables were analyzed utilizing the χ^2^ test and Fisher’s exact test. The estimated variation within each group was similar. Correlations between variables were assessed employing Spearman’s correlation coefficient. Each experiment was performed independently three times and the quantitative data were shown as mean ± SD unless specified otherwise. Statistical analyses and graphical presentations were completed with the help of R software version 4.3.2 and GraphPad Prism version 8.3.0. A *P* value of less than 0.05 was deemed statistical significance unless otherwise noted.

## Results

### Down-regulation of AMDHD1 correlates with adverse clinical outcomes in CCA

**Fig. 1:**
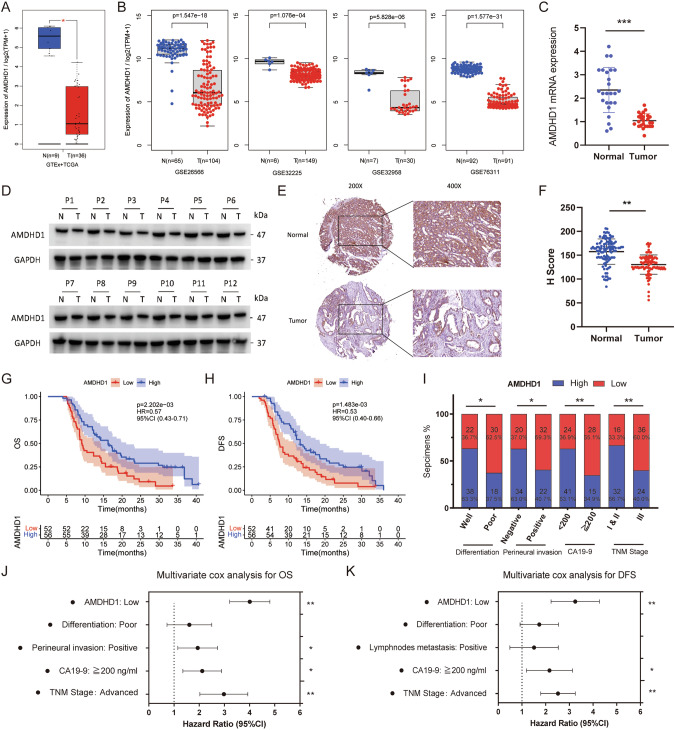
Down-regulation of AMDHD1 correlates with adverse clinical outcomes in cholangiocarcinoma (CCA). **A** Low mRNA expression of AMDHD1 in CCA was identified in GEPIA database. **B** Down-regulation of AMDHD1 was identified in CCA in 4 individual GEO datasets (GSE26566, GSE32225, GSE32958 and GSE76311). **C** The low mRNA expressions of AMDHD1 were identified in CCA tissues of 25 pairs samples. **D** The low protein expressions of AMDHD1 were identified in CCA tissues of 12 pairs samples. **E** Representative images of AMDHD1 staining in CCA specimens and normal bile duct tissues. **F** Immunohistochemistry staining score showed the protein levels of AMDHD1 were down-regulated in CCA tissues. Kaplan–Meier analyses showed CCA patients with high expression of AMDHD1 had superior overall survival (**G**) and disease-free survival (**H**) than those with low expression. **I** AMDHD1 expression in CCA was significantly correlated with tumor differentiation, perineural invasion, CA19-9 index and TNM stage. Multivariate Cox regression analyses showed low expression of AMDHD1 was independent risk factor for overall survival (**J**) and disease-free survival (**K**) of 108 CCA patients. N, normal; T, tumor; OS, overall survival; DFS, disease-free survival. CA19-9, carbohydrate antigen 19-9. All *P < 0.05, **P < 0.01, ***P < 0.001. *P* values were determined by Non-parametric Mann-Whitney U-test in (**A**, **B**). *P* values were assessed by two-tailed *t*-tests in (**C**, **F**). *P* values were determined by the log-rank (Mantel-Cox) test in (**G**, **H**). *P* values were determined by χ2 tests or Fisher’s exact tests in (**I**). *P* values were determined by cox regression analysis in (**J**, **K**).

Initially, differential analyses utilizing public databases were conducted to investigate the role of AMDHD1 during tumorigenesis. The GEPIA database indicated the lower expression of AMDHD1 in various types of cancer (Fig. S1A) and particularly the mRNA expression of AMDHD1 was notably down-regulated in CCA compared with normal bile duct tissues (Fig. 1A). Furthermore, four GEO datasets, namely GSE32958, GSE26566, GSE76311 and GSE32225, consistently demonstrated that AMDHD1 expression was reduced in CCA compared to normal tissues (Fig. 1B). Complementary analyses involving RT-qPCR on 25 pairs of fresh tumor specimens and western blot analysis on 12 pairs of tumor specimens from PUMCH confirmed the decreased mRNA and protein expression levels of AMDHD1 in CCA tissues (Fig. 1C, D). The H scores of IHC from 108 pairs of CCA and adjacent normal tissues sourced from PUMCH and GPPH further validated AMDHD1’s down-regulation in CCA (Fig. 1E, F). Subsequently, 108 CCA patients were categorized into high-AMDHD1 and low-AMDHD1 groups based on the H scores. Kaplan-Meier analyses revealed that patients with lower AMDHD1 levels exhibited significantly worse OS and disease free survival (DFS) compared to those with higher levels (Fig. 1G, H). Additionally, low AMDHD1 expression was associated with adverse clinical characteristics such as poor tumor differentiation, presence of perineural invasion, elevated CA19-9 levels and advanced TNM stage (Fig. 1I and Table S3). Cox regression analyses established the down-regulation of AMDHD1 as an independent prognostic risk factor for both OS and DFS in CCA (Fig. 1J, K and Tables S4, S5). Moreover, the association between low AMDHD1 expression and poorer OS was also observed in other cancer types, including kidney renal clear cell carcinoma, liver hepatocellular carcinoma, pancreatic adenocarcinoma (PAAD), sarcoma, skin cutaneous melanoma and uveal melanoma (Fig. S1B). Collectively, these results underscore the potential of AMDHD1 as a prognostic marker for CCA.

### AMDHD1 inhibits the proliferation and migration of CCA cells in vitro and in vivo

**Fig. 2:**
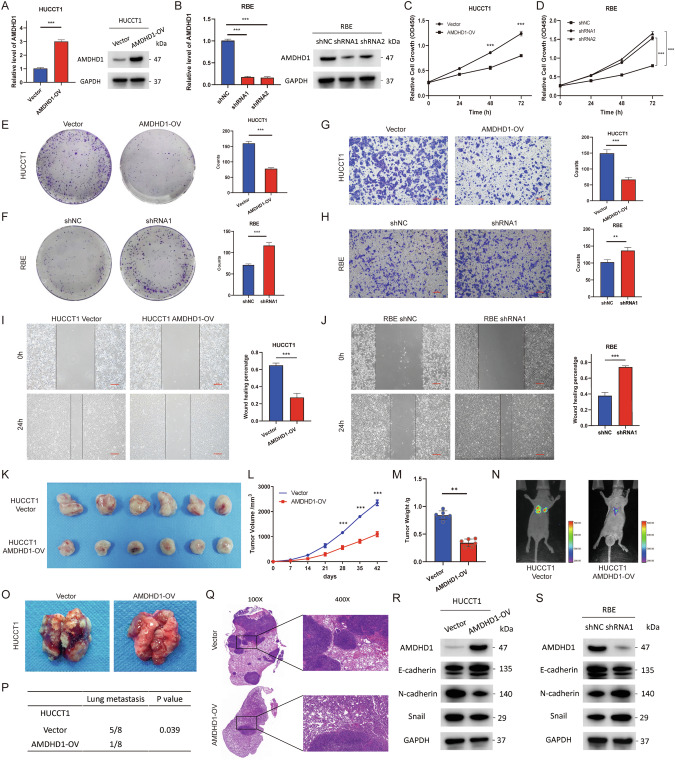
AMDHD1 inhibits the proliferation and migration of cholangiocarcinoma (CCA) cells in vitro and in vivo. RT-qPCR and western blot were used to detect the over-expression or knockdown of AMDHD1 in HUCCT1 (**A**) and RBE cells (**B**). CCK-8 assays were used to detect the proliferation of HUCCT1/AMDHD1-OV (**C**) and RBE/AMDHD1-KD cells (**D**). Colony formation assays were used to detect the proliferation of HUCCT1/AMDHD1-OV (**E**) and RBE/AMDHD1-KD cells (**F**). Transwell assays were used to detect the migration of HUCCT1/AMDHD1-OV (**G**) and RBE/AMDHD1-KD cells (**H**). Wound-healing assays were used to detect the migration of HUCCT1/AMDHD1-OV (**I**) and RBE/AMDHD1-KD cells (**J**). **K** Xenograft models (*n* = 6 per group) were established and tumors dissected from respective groups were shown. **L** Tumor growth curves showed the over-expression of AMDHD1 retarded tumor growth rate. **M** Tumor weight was measured and a tumor weight decrease was observed in AMDHD1-OV group compared with control group. **N**–**P** Lung metastasis models (*n* = 6 per group) were established and the quantitation of lung metastasis was assessed by bioluminescence measurements (**N**). Lungs of mice were dissected from respective groups (**O**) and metastases were obviously inhibited in the AMDHD1-OV group compared to control group (**P**). **Q** Representative H&E images of lung metastatic tumors from indicated groups. Western blot showed over-expression of AMDHD1 up-regulated E-cadherin and declined N-cadherin and Snail in HUCCT1 cells (**R**), whereas AMDHD1 knockdown reduced E-cadherin and increased N-cadherin and Snail in RBE cells (**S**). All *P < 0.05, **P < 0.01, ***P < 0.001. Scale bars in (**G**, **H**): 100 μm. Scale bars in (**I**, **J**): 250 μm. *P* values were assessed using two-tailed *t* tests and ANOVA followed by Dunnett’s tests for multiple comparison in (**A**–**J**, **L**, **M**). *P* values were determined by χ2 tests or Fisher’s exact tests in P.

The mRNA and protein expressions of AMDHD1 were assessed across several CCA cell lines. Our findings suggested AMDHD1 was expressed relatively high in RBE and HCCC-9810 cells, while its expression was notably lower in HUCCT1 and QBC939 cells (Fig. S2A, B). The HUCCT1 and QBC939 cell lines were subsequently engineered to stably overexpress AMDHD1 to facilitate gain-of-function analysis (Fig. 2A and Fig. S3A). Conversely, two sh-AMDHD1 were introduced into RBE and HCCC-9810 cells to knockdown AMDHD1 expression. ShRNA1, which demonstrated superior knockdown efficiency, was selected for subsequent loss-of-function analyses (Fig. 2B and Fig. S3B). The cell morphology post AMDHD1 overexpression or knockdown were documented in Fig. S2C–F. Following the verification of the AMDHD1 expression by RT-qPCR and western blot analyses, we evaluated the effects on cell viability and proliferation by CCK8 and colony formation assays. It’s revealed that AMDHD1 overexpression inhibited the proliferation of HUCCT1 and QBC939 cells, whereas knockdown of AMDHD1 increased proliferative activity in RBE and HCCC-9810 cells (Fig. 2C–F and Fig. S3C–F). Additionally, cell migration assessments, utilizing Transwell and wound healing assays, showed AMDHD1 overexpression suppressed HUCCT1 and QBC939 cells migratory capabilities, while AMDHD1 down-regulation enhanced the migratory abilities of RBE and HCCC-9810 cells (Fig. 2G–J and Fig. S3G–J).

To further investigate the impact of AMDHD1 on tumor growth and metastasis in vivo, HUCCT1/AMDHD1-OV and control cells were injected into BALB/c-nude mice to establish xenograft and lung metastasis models. Results from xenograft models indicated that AMDHD1 overexpression significantly reduced tumor growth rates and resulted in lower tumor weights in the AMDHD1-OV group compared with controls at the study’s conclusion (Fig. 2K–M). Similarly, lung metastasis models revealed pronounced inhibition of metastases in the AMDHD1-OV group eight weeks post injection compared to controls (Fig. 2N–P). H&E staining of lung sections confirmed these findings (Fig. 2Q). Western blot further demonstrated AMDHD1 overexpression led to an upregulation of E-cadherin and significant downregulation of N-cadherin and Snail (Fig. 2R and Fig. S3K), whereas activation of the epithelial-mesenchymal transition (EMT) was observed in AMDHD1-KD cells (Fig. 2S and Fig. S3L). Collectively, these results indicate that AMDHD1 significantly inhibits the proliferation and migration of CCA cells in vitro and in vivo, underscoring its potential as a therapeutic target in CCA treatment.

### AMDHD1 promotes apoptosis and arrests the cell cycle of CCA cells

**Fig. 3:**
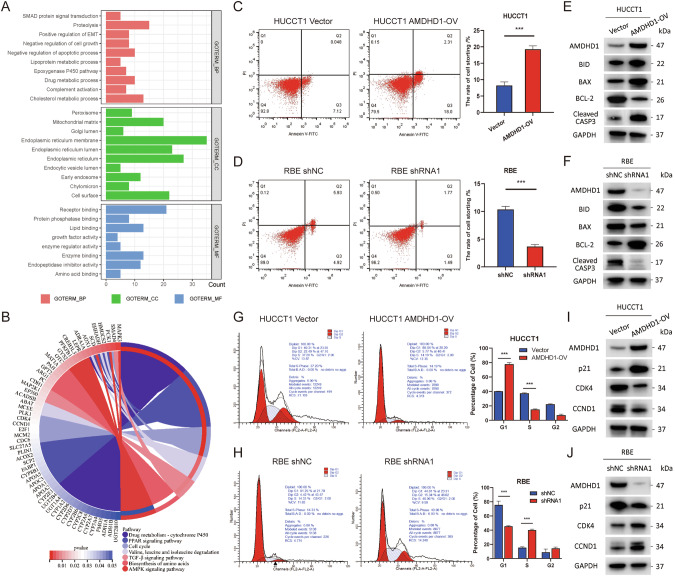
AMDHD1 promotes apoptosis and arrests cell cycle of cholangiocarcinoma (CCA) cells. **A** Gene Ontology enrichment analysis of AMDHD1 based on cBioPortal database. **B** Kyoto Encyclopedia of Genes and Genomes pathway analysis of AMDHD1 based on cBioPortal database. Flow cytometry was used to detect the percentage of apoptotic cells in HUCCT1/AMDHD1-OV (**C**) and RBE/AMDHD1-KD cells (**D**). Western blot analysis of protein levels of AMDHD1, BAX, cleaved CASP3, BID and BCL-2 in HUCCT1/AMDHD1-OV (**E**) and RBE/AMDHD1-KD cells (**F**). Flow cytometry was used to detect the cell cycle of HUCCT1/AMDHD1-OV (**G**) and RBE/AMDHD1-KD cells (**H**). Western blot analysis of protein levels of AMDHD1, p21, CDK4 and CCND1 in HUCCT1/AMDHD1-OV (**I**) and RBE/AMDHD1-KD cells (**J**). All ***P < 0.001. *P* values were assessed using two-tailed t-tests in (**C**, **D**, **G**, **H**).

To delve deeper into the physiological functions of AMDHD1, we identified 304 co-expressed genes associated with AMDHD1 using the cBioPortal database and performed GO and KEGG analyses. The GO analysis suggested that AMDHD1 might be predominantly localized in the cytoplasm and implicated in processes such as proteolysis, cell growth, apoptosis, EMT and SMAD protein signal transduction (Fig. 3A). The KEGG analysis indicated that AMDHD1 and its co-expressed genes may involve in regulating the cell cycle, TGF-β signaling pathway, AMPK signaling pathway and the biosynthesis of amino acids (Fig. 3B).

Building on these insights, we employed flow cytometry to examine AMDHD1’s specific role in apoptosis and the cell cycle. Our data indicated that overexpression of AMDHD1 increased the percentage of apoptotic HUCCT1 and QBC939 cells, while knockdown of AMDHD1 reduced apoptosis in RBE and HCCC-9810 cell lines (Figs. 3C, D and S4A, B). Further investigation into apoptosis modulators via western blot analysis revealed that AMDHD1 overexpression upregulated the expression of BAX and cleaved CASP3, while decreased the levels of BID and BCL-2 (Figs. 3E and S4C). Conversely, knockdown of AMDHD1 resulted in reduced expression of BAX and cleaved CASP3, as well as increased levels of BID and BCL-2 (Figs. 3F and S4D). The findings collectively suggest that AMDHD1 can activate the apoptotic pathway in CCA cells, underscoring its potential significance in cancer biology.

Flow cytometry analyses, as depicted in Fig. 3G, H and Fig. S4E, F, demonstrated that overexpression of AMDHD1 caused an accumulation of HUCCT1 and QBC939 cells in the G1/S phase of the cell cycle, whereas AMDHD1 knockdown in HUCCT1 cell lines resulted in an increased percentage of cells in the G1 phase. The expression levels of cyclins and cyclin-dependent kinases were examined and the results showed an upregulation of the cell cycle inhibitor p21 and a decrease in CDK4 and CCND1 levels in CCA cells overexpressing AMDHD1 (Figs. 3I and S4G). In contrast, cells with AMDHD1 knockdown exhibited reduced levels of p21 and increased expressions of CDK4 and CCND1 (Figs. 3J and S4H). Given that CDK4 and CCND1 are critical regulators of the transition from G1 phase to S phase [19], our findings suggest that AMDHD1 can impede G1/S progression through the p21/CDK4 pathway, highlighting its potential regulatory role in the cell cycle of CCA cells.

### AMDHD1 functions in a TGF-β dependent manner

**Fig. 4:**
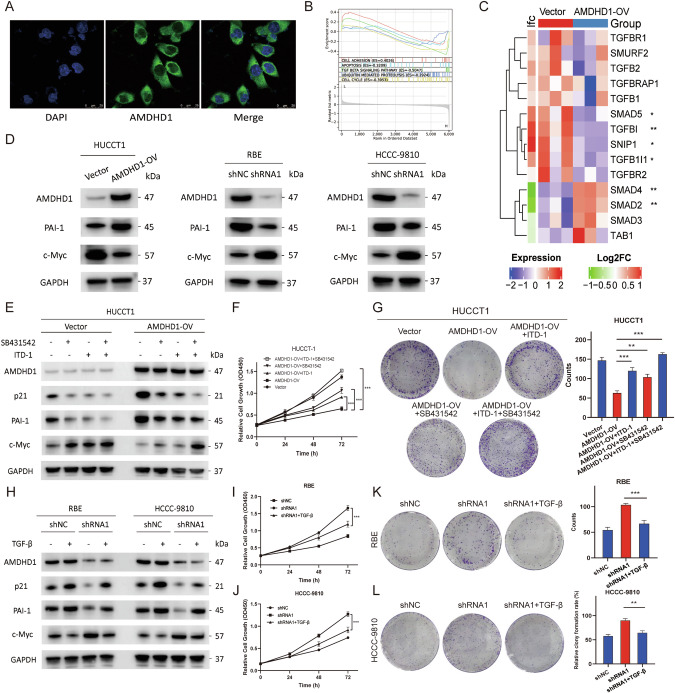
AMDHD1 functions in a TGF-β dependent manner. **A** Immunofluorescence staining for the intracellular localization of AMDHD1 in HUCCT1 cells. **B** Gene set enrichment analysis of Tandem Mass Tag proteomic analysis on HUCCT1/AMDHD1-OV and control cells groups. **C** Heatmap of proteomic analysis showed differential expression levels of key molecules in TGF-β signaling pathway. **D** Western blot was used to detect the PAI-1 and c-Myc expressions in AMDHD1-OV and AMDHD1-KD cells. **E** HUCCT1/AMDHD1-OV cells were treated with SB431542 or ITD-1 and the protein levels of AMDHD1, p21, PAI-1 and c-Myc were detected by immunoblotting. CCK-8 assays (**F**) and colony formation assays (**G**) were performed in HUCCT1/AMDHD1-OV cells treated with SB431542 or ITD-1. **H** The protein levels of AMDHD1, p21, PAI-1 and c-Myc were detected by immunoblotting in RBE/AMDHD1-KD and HCCC-9810/AMDHD1-KD cells treated with TGF-β. CCK-8 assays were performed in RBE/AMDHD1-KD cells (**I**) and HCCC-9810/AMDHD1-KD cells (**J**) treated with TGF-β. Colony formation assays were performed in RBE/AMDHD1-KD cells (**K**) and HCCC-9810/AMDHD1-KD cells (**L**) treated with TGF-β. All *P < 0.05, **P < 0.01, ***P < 0.001. *P* values were assessed using two-tailed t-tests and ANOVA followed by Dunnett’s tests for multiple comparisons in (**C**, **F**, **G**, **I**, **J**, **K**, **L**).

AMDHD1 is predominantly localized in the cytoplasm, as illustrated in Fig. 4A, and is implicated in the regulation of proteolysis (Fig. 3A). To further investigate its downstream pathways and molecules, TMT proteomic analysis was performed using a Q Exactive™ HF-X mass spectrometer on HUCCT1/AMDHD1-OV and control cells and identified 483 proteins as differentially expressed proteins (|Log2Fold Change | > 0.5). GSEA was conducted to discern the pathways differentially activated between high- and low-AMDHD1 expression groups. As depicted in Fig. 4B, significant enrichment was observed in biological processes such as cell adhesion, ubiquitin mediated proteolysis, apoptosis, cell cycle, and notably, the TGF-β signaling pathway. To further explore AMDHD1’s role within TGF-β pathway, we assessed the expression of key TGF-β signaling molecules based on the proteomic data. Our findings indicated reduced expressions of SMAD5, TGFBI, SNIP1 and TGFB1I1 in AMDHD1-OV cells, while expressions of SMAD4 and SMAD2 were increased (Fig. 4C). Additionally, we investigated AMDHD1’s specific impact on TGF-β-induced transcription of known direct targets within TGF-β signaling pathway, including PAI-1, c-Myc and p21 [20–22]. Typically, TGF-β stimulation suppresses c-Myc expression while enhancing PAI-1 and p21 expressions [23]. Consistent with expectations, AMDHD1 overexpression in HUCCT1 cells significantly augmented TGF-β responses, evidenced by increased expression of p21, PAI-1 and decreased expression of c-Myc. Conversely, p21 and PAI-1 levels were reduced and c-Myc levels were increased following AMDHD1 knockdown (Fig. 4D and Fig. S5A). These findings suggest that AMDHD1 potentially enhances TGF-β signaling pathway.

To further investigate whether AMDHD1 functions in a TGF-β dependent manner, inhibitors SB431542 and ITD-1 were utilized in rescue experiments to suppress the activities of TGF-β1 and TGF-β2 signaling, respectively [24, 25]. The results indicated that either individual or combined treatment with SB431542 and ITD-1 in AMDHD1-OV cells significantly mitigated the AMDHD1-induced upregulation of PAI-1 and p21, while alleviating the AMDHD1-induced decrease in c-Myc expression (Fig. 4E). Subsequently, cell viability and proliferation assessments via CCK-8 and colony formation assays revealed that inhibition of TGF-β signaling reversed the suppression of cell proliferation induced by AMDHD1 overexpression (Fig. 4F, G). Conversely, the upregulation of c-Myc and downregulation of p21 and PAI-1 observed in AMDHD1-KD cells were counteracted by TGF-β stimulation (Fig. 4H). Additionally, cell proliferation assays demonstrated that TGF-β treatment hindered the increased cell viability caused by the deletion of AMDHD1 (Fig. 4I–L). Collectively, AMDHD1 exerts its regulatory effects on the malignant phenotype of CCA cells through a TGF-β dependent pathway.

### Identifying the downstream effectors of AMDHD1 in TGF-β signaling pathway

**Fig. 5:**
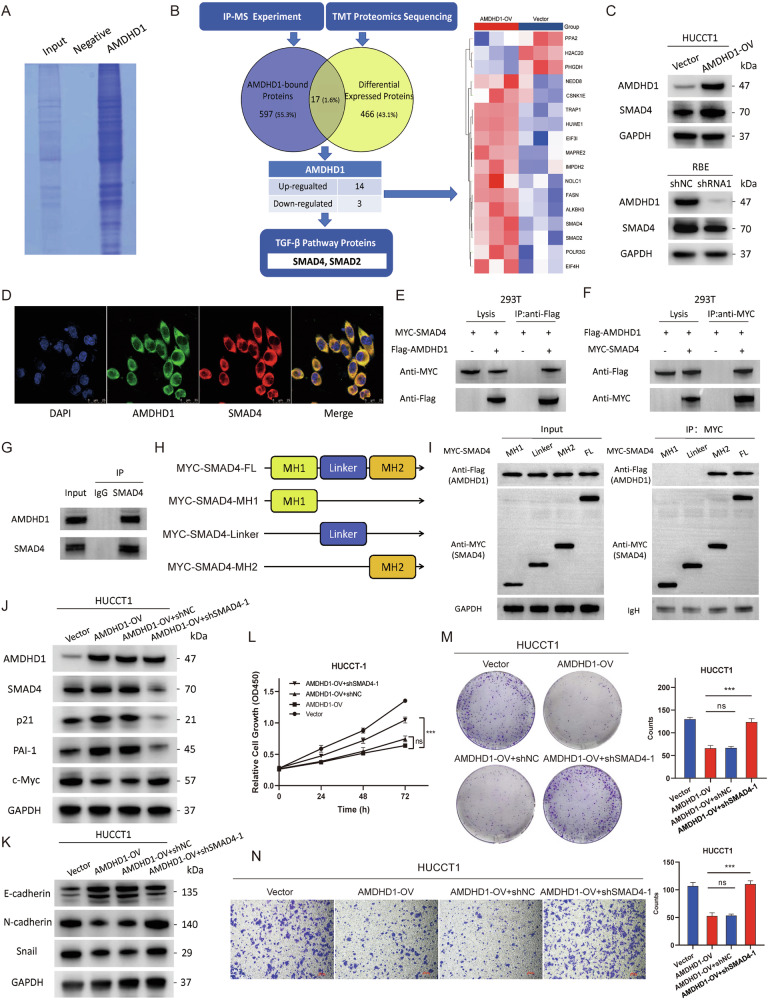
Identification of AMDHD1 downstream effectors in TGF-β pathway. **A** Coomassie blue staining showing the results of an immunoprecipitation assay with AMDHD1. **B** Flow diagram of the bioinformatic analyses and selection of downstream gene candidates targeted by AMDHD1. Venn diagram depicts the overlap between 483 differentially expressed proteins upon AMDHD1 over-expression (yellow) and 614 AMDHD1-bound proteins (blue). Among 17 overlapping proteins, 14 proteins were up-regulated and 3 proteins were down-regulated in HUCCT1/AMDHD1-OV cells. 2 downstream candidate proteins (SMAD4 and SMAD2) were finally selected through STRING functional enrichment analyses. **C** Western blot was used to detect the SMAD4 levels in AMDHD1 over-expressed and knockdown cells. **D** Double-label immunofluorescence staining for the intracellular localization of AMDHD1 and SMAD4 in HUCCT1 cells. HEK293T cells were transfected with Flag-tagged AMDHD1 and MYC-tagged SMAD4 expression plasmids as indicated, whole cell lysates were extracted and immunoprecipitated with anti-Flag antibodies (**E**) or anti-MYC antibodies (**F**). **G** Lysates of HUCCT1 cells were immunoprecipitated with SMAD4 antibody or normal rabbit IgG, then subjected to immunoblotting with AMDHD1 or SMAD4 antibody. **H** The schematic illustration of SMAD4 and its truncated fragments were shown. **I** Total lysates from HUCCT1 cells overexpressing MYC-tagged full-length or truncated SMAD4 were subjected to IP with anti-MYC Ab, followed by western blotting using the indicated antibodies (Abs). Ig heavy chain (H chain) was used as a loading control. **J**, **K** HUCCT1/AMDHD1-OV cells were transfected with SMAD4-shRNA and the protein levels of AMDHD1, p21, PAI-1, c-Myc, E-cadherin, N-cadherin and Snail were analyzed by immunoblotting. CCK-8 assays (**L**), colony formation assays (**M**) and transwell assays (**N**) were performed in HUCCT1/AMDHD1-OV cells transfected with SMAD4-shRNA. All ns not significant, ***P < 0.001. Scale bars: 200 μm. *P* values were assessed using two-tailed t-tests and ANOVA followed by Dunnett’s tests for multiple comparison in (**B**, **L**, **M**, **N**).

To elucidate how AMDHD1 potentiates TGF-β-induced responses, we performed immunoprecipitation mass spectrometry (IP-MS) on AMDHD1-OV cells to identify proteins that interact with AMDHD1 and provide a deeper mechanistic insight (Fig. 5A). Our findings revealed 614 significant AMDHD1-binding proteins, primarily involved in the regulation of SMAD protein signal transduction, protein phosphorylation, cyclin-dependent protein activity, proteolysis and EMT (Fig. S5B). Moreover, KEGG pathway analysis indicated that AMDHD1-binding proteins were likely associated with processes such as protein processing in the endoplasmic reticulum, apoptosis, cell cycle, TGF-β signaling pathway and ubiquitin-mediated proteolysis (Fig. S5C), aligning closely with our previous findings. Subsequently, we conducted a Venn diagram analysis to compare the 483 differentially expressed proteins identified through TMT proteomic analysis with the 614 AMDHD1-binding proteins revealed by IP-MS, identifying 17 proteins common to both datasets (Fig. 5B). These 17 candidate proteins were then analyzed using the STRING database for functional enrichment to assess their connections to TGF-β signaling pathway (Fig. S5D). Finally SMAD4 and SMAD2 were pinpointed as potential downstream effectors of AMDHD1 within TGF-β signaling pathway and were selected for further investigation. Additionally, the expressions of other pivotal molecules within TGF-β pathway, including TGFB1, TGFB2, TGFBR1 and TGFBR2, remained unchanged with the overexpression or knockdown of AMDHD1 (Fig. S5E).

SMAD4, a pivotal mediator of TGF-β signaling pathway, is recognized as a tumor suppressor in various cancers, including CCA. The initial discovery of SMAD4 deletion in PAAD has been extended to multiple cancer types [12]. In CCA, SMAD4 is among the most frequently mutated genes and has been shown to curtail cell proliferation, tumor progression and drug resistance through TGF-β and Wnt/β-catenin signaling pathways [12, 26]. In our research, we demonstrated that the expression of SMAD4 was reduced in CCA compared to normal bile duct tissues by IHC (Fig. S5F). Further the protein levels of SMAD4 were found elevated in AMDHD1-OV cells and diminished following AMDHD1 depletion (Figs. 5C and S5G). Notably, the mRNA levels of SMAD4 remained unchanged in both AMDHD1-OV and AMDHD1-KD cells (Fig. S5H), suggesting that AMDHD1 may exert its effects on SMAD4 expression through translational or post-translational mechanisms. Confocal microscopy results demonstrated significant colocalization of AMDHD1 and SMAD4 in the cytoplasm of HUCCT1 cells (Fig. 5D). Moreover, Flag-tagged AMDHD1 and MYC-tagged SMAD4 were ectopically expressed in HEK293T cells, followed by an IP experiment, which indicated a direct interaction between AMDHD1 and SMAD4 (Fig. 5E, F). Subsequent endogenous CO-IP experiments confirmed this interaction in HUCCT1 cells, eliminating the possibility of artifactual interactions (Fig. 5G). To delineate the specific region of SMAD4 required for interaction with AMDHD1, MYC-tagged full-length SMAD4 and various deletion mutants were co-transfected into HEK293T cells along with an AMDHD1 expression vector (Fig. 5H). IP results identified the MH2 domain of SMAD4 as essential for binding to AMDHD1 (Fig. 5I).

To ascertain whether SMAD4 is crucial for AMDHD1’s functions, we conducted rescue experiments in which shRNAs targeting SMAD4 were transfected into AMDHD1-OV cells to achieve SMAD4 knockdown (Fig. S5I). As expected, SMAD4 depletion significantly counteracted the AMDHD1-induced upregulation of PAI-1, p21 and E-cadherin, while mitigating the reduction of c-Myc, Snail and N-cadherin (Fig. 5J, K). Cell viability and proliferation, assessed through CCK-8 and colony formation assays, revealed that the suppression of cell viability caused by AMDHD1 overexpression was negated by the downregulation of SMAD4 (Fig. 5L, M). Additionally, Transwell assays demonstrated that the migration inhibition resulting from AMDHD1 overexpression was reversed following SMAD4 knockdown (Fig. 5N). We further extended our investigation by transfecting an SMAD4 expression vector into AMDHD1-KD cells to assess functional recovery. The results indicated that overexpression of SMAD4 effectively reversed the AMDHD1 deletion-induced decreases in PAI-1, p21 and E-cadherin levels, as well as the increases in c-Myc, N-cadherin and Snail levels (Fig. S6A, B). Additionally, cell proliferation and migration assays demonstrated that the heightened proliferative and migratory capacities observed in CCA cells following AMDHD1 deletion were inhibited upon SMAD4 restoration (Fig. S6C–E). In summary, these results firmly establish that SMAD4 plays a critical role in mediating the effects of AMDHD1 on the malignant phenotype of CCA cells.

### AMDHD1 inhibits the ubiquitination and degradation of SMAD4

**Fig. 6:**
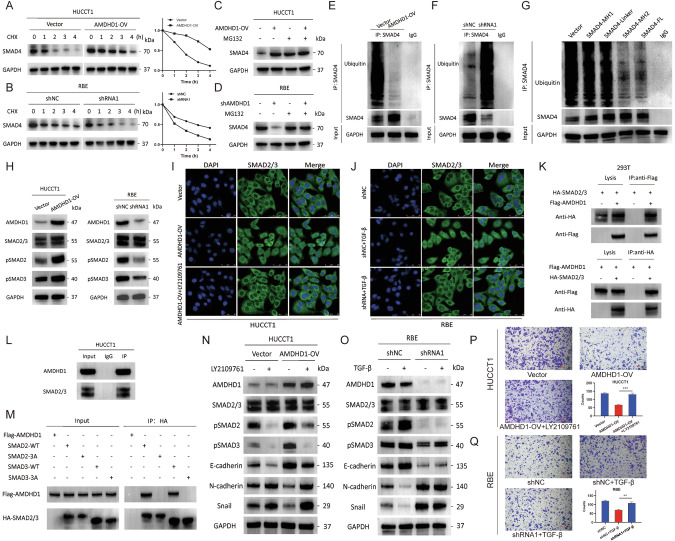
AMDHD1 inhibites the ubiquitination degradation of SMAD4 and promotes SMAD2/3 phosphorylation. Western blot showed the half-life of SMAD4 in HUCCT1/AMDHD1-OV (**A**) and RBE/AMDHD1-KD cells (**B**). The expression of SMAD4 in HUCCT1/AMDHD1-OV (**C**) and RBE/AMDHD1-KD cells (**D**) treated with MG132 was determined by Western blot. Western blot analysis of ubiquitinated SMAD4 immunoprecipitated from HUCCT1/AMDHD1-OV (**E**) and RBE/AMDHD1-KD cells (**F**) treated with MG132. **G** Western blot analysis of ubiquitinated SMAD4 immunoprecipitated from HUCCT1 cells over-expressing MYC-tagged full-length or truncated SMAD4 protein. **H** Western blot was used to detect the SMAD2/3, pSMAD2 and pSMAD3 levels in HUCCT1/AMDHD1-OV and RBE/AMDHD1-KD cells. **I** The intracellular localization of SMAD2/3 was determined by immunofluorescence in HUCCT1/AMDHD1-OV cells treated with LY2109761. **J** The intracellular localization of SMAD2/3 was determined by immunofluorescence in RBE/AMDHD1-KD cells treated with TGF-β. **K** HEK293T cells were transfected with Flag-tagged AMDHD1 and HA-tagged SMAD2/3 expression plasmids as indicated, whole cell lysates were extracted and immunoprecipitated with anti-Flag antibodies or anti-HA antibodies. **L** Lysates of HUCCT1 cells were immunoprecipitated with SMAD2/3 antibody or normal rabbit IgG, and then subjected to immunoblot with AMDHD1 or SMAD2/3 antibody. **M** Three Serine residues (464, 465 and 467) in SMAD2/3 were converted to alanine to establish SMAD2-A and SMAD3-A mutants. CO-IP assays showed SMAD2-A and SMAD3-A mutants could not pulldown AMDHD1 compared to wild-type SMAD2/3. **N** Western blot analysis of protein levels of AMDHD1, SMAD2/3, pSMAD2, pSMAD3, E-cadherin, N-cadherin and Snail from HUCCT1/AMDHD1-OV cells treated with LY2109761. **O** Western blot analysis of protein levels of AMDHD1, SMAD2/3, pSMAD2, pSMAD3, E-cadherin, N-cadherin and Snail from RBE/AMDHD1-KD cells treated with TGF-β. Transwell assays were performed in HUCCT1/AMDHD1-OV cells treated with LY2109761 (**P**) and RBE/AMDHD1-KD cells treated with TGF-β (**Q**). All ns not significant, **P < 0.01, ***P < 0.001. Scale bars: 200 μm. *P* values were assessed using two-tailed t-tests and ANOVA followed by Dunnett’s tests for multiple comparison in (**P**, **Q**).

To identify the specific translational or post-translational modification exerted by AMDHD1 on SMAD4 expression, we assessed the degradation half-life of SMAD4 protein using cycloheximide (CHX) treatment. Our results demonstrated that AMDHD1 significantly increased the stability of SMAD4 protein and extended its half-life (Fig. 6A). Conversely, when AMDHD1 was knocked down, SMAD4 protein degradation accelerated, and its half-life was reduced over time (Fig. 6B), indicating that AMDHD1 enhanced SMAD4 stability by inhibiting its protein degradation. Given that the ubiquitin-proteasome pathway is predominantly responsible for SMAD4 protein degradation [27, 28], we investigated the deeper regulatory mechanism behind AMDHD1-induced SMAD4 stabilization. The proteasome inhibitor (MG132) was used to treat HUCCT1 cells with either upregulated or downregulated AMDHD1. Western blot analysis revealed that SMAD4 protein levels did not increase after MG132 treatment in AMDHD1-OV cells (Fig. 6C), while MG132 significantly restored SMAD4 protein levels in AMDHD1-KD cells (Fig. 6D), suggesting that AMDHD1-induced stabilization of SMAD4 involved the ubiquitin-proteasome pathway. Next, we examined the polyubiquitination levels of SMAD4 protein in AMDHD1-OV and AMDHD1-KD cells. As anticipated, SMAD4 ubiquitination was substantially reduced with the overexpression of AMDHD1 and increased following AMDHD1 knockdown (Fig. 6E, F). To further confirm the interaction between AMDHD1 and SMAD4, deletion mutants of SMAD4 were co-transfected with the AMDHD1 vector into HEK293T cells. It’s suggested the MH2 domain of SMAD4 was indispensable for the AMDHD1-induced suppression of SMAD4 ubiquitination (Fig. 6G), aligning with our previous findings. In summary, these results clarify that AMDHD1 reduces the ubiquitination of SMAD4 protein, thereby preventing its degradation.

### AMDHD1 interacts with SMAD2/3 and promotes their phosphorylation

The TGF-β/SMAD signaling pathway plays a crucial role in EMT, with SMAD4 acting as an essential cofactor. SMAD4 binds to phosphorylated and activated SMAD2/3, facilitating their nuclear translocation and subsequent transcriptional regulation of target genes [11]. Previously, we identified SMAD2 as a potential target of AMDHD1 within TGF-β pathway (Fig. 5B). However, alterations in the protein expression of SMAD2/3 were not detected following the overexpression or knockdown of AMDHD1 in western blot analyses (Fig. 6H). Interestingly, the overexpression of AMDHD1 significantly increased the expression of phosphorylated SMAD2/3 (pSMAD2/3), whereas AMDHD1 deletion markedly reduced pSMAD2/3 levels (Fig. 6H). IF assays demonstrated that SMAD2/3 translocation to the nucleus was induced by AMDHD1 overexpression in HUCCT1 cells. This translocation was inhibited by LY2109761, a dual inhibitor of TGF-β receptor types I and II (Fig. 6I) [29]. In RBE cells stimulated with TGF-β, we observed an enhancement in the nuclear translocation of SMAD2/3, which was reversed by AMDHD1 silencing (Fig. 6J). Further investigations involved co-transfecting Flag-AMDHD1 and HA-SMAD2/3 into HEK293T cells for CO-IP experiments, where we discovered an interaction between AMDHD1 and SMAD2/3 (Fig. 6K), which was confirmed by endogenous CO-IP experiments in HUCCT1 cells (Fig. 6L). To determine the necessity of SMAD2/3 phosphorylation for their interaction with AMDHD1, we mutated three serine residues in SMAD2/3 (464, 465 and 467) — crucial for TGFBR1-mediated phosphorylation and activation of TGF-β/SMADs pathway — to alanine, resulting in SMAD2-A and SMAD3-A mutants [30]. These mutants failed to pulldown AMDHD1 compared to the wild-type SMAD2/3 (Fig. 6M). Additional rescue experiments showed that the phosphorylation of SMAD2/3 induced by AMDHD1, along with the inhibition of EMT and suppression of migration, was reversed upon treatment with LY2109761 (Fig. 6N, P). Conversely, silencing AMDHD1 impeded the phosphorylation of SMAD2/3, activation of EMT, and enhancement of migration triggered by TGF-β stimulation (Fig. 6O, Q). Collectively, our results suggest that AMDHD1 promotes the phosphorylation of SMAD2/3 and the signaling of TGF-β/SMADs pathway through its interaction with SMAD2/3.

### AMDHD1 correlates with SMAD4 expression in CCA

**Fig. 7:**
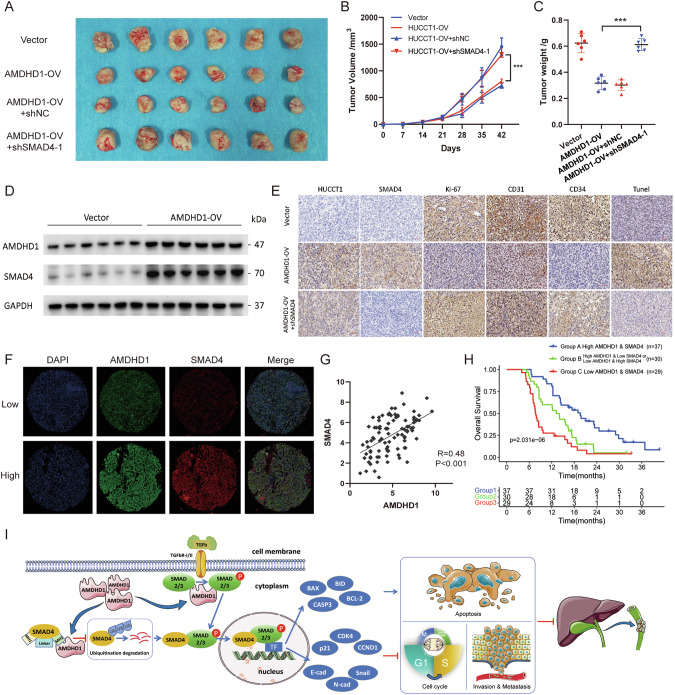
AMDHD1 correlates with SMAD4 in cholangiocarcinoma (CCA). **A** BALB/c-nudes (*n* = 6 per group) were euthanized 42 days after the injection of HUCCT1/AMDHD1-OV cells transfected with or without SMAD4-shRNA and tumors dissected from respective groups were shown. **B** Tumor growth curves of respective groups were shown. **C** Tumor weight was measured in respective groups. **D** The protein levels of AMDHD1 and SMAD4 of tumor tissues from xenograft models were analyzed by immunoblotting. **E** IHC staining of AMDHD1, SMAD4, Ki67, CD31, CD34, and Tunel in tumor tissues from xenograft models. **F** Representative images of double-label immunofluorescence staining of AMDHD1 and SMAD4 in 96 CCA tissues. **G** IF staining showed AMDHD1 expression level highly correlated with SMAD4 expression. **H** Kaplan–Meier analysis in CCA patients groups showed that patients with low AMDHD1/SMAD4 expression had the worst overall survival among all the groups. **I** A schematic diagram for a novel regulatory mechanism that AMDHD1 inhibited the ubiquitination degradation of SMAD4 protein and synergistically promoted SMAD2/3 phosphorylation, which activates TGF-β signaling pathway and regulates cell cycle, apoptosis and metastasis in CCA. All ***P < 0.001. Scale bars: 50 μm. *P* values were assessed using two-tailed t-tests and ANOVA followed by Dunnett’s tests for multiple comparison in (**B**, **C**). Spearman’s correlation was performed in (**G**). Kaplan–Meier analyses and log-rank tests were conducted in (**H**).

To examine the association between AMDHD1 and SMAD4 in vivo, xenograft mouse models were established by injecting either control cells or HUCCT1/AMDHD1-OV cells treated with control adenovirus, SMAD4 knockdown adenovirus into BALB/c-nude mice (Fig. 7A). As anticipated, we observed that the inhibition of tumor growth induced by overexpression of AMDHD1 was reversed after SMAD4 depletion (Fig. 7B, C). The protein expression of SMAD4 was upregulated in the tumor tissues of the AMDHD1-OV group (Fig. 7D). IHC results revealed that the expression levels of Ki67, CD31, and CD34 were decreased in the AMDHD1-OV group compared to the control group, and the reductions were negated upon SMAD4 downregulation (Fig. 7E). Additionally, the level of TUNEL staining was enhanced in the AMDHD1 overexpression group, which decreased following SMAD4 knockdown (Fig. 7E). To extend these findings, double-labeled IF staining was employed to evaluate CCA tissues from 96 patients from PUMCH and GPPH (Fig. 7F). A significant positive correlation (*R* = 0.48, *P* < 0.001) was detected between the expressions of AMDHD1 and SMAD4 (Fig. 7G). Based on the levels of AMDHD1 and SMAD4 expression, CCA patients were classified into three groups: Group A with high expression of both AMDHD1 and SMAD4 (*n* = 37), Group B with mixed expression profiles (high AMDHD1 and low SMAD4 expression or vice versa; *n* = 30), and Group C with low expression of both AMDHD1 and SMAD4 (*n* = 29). Kaplan–Meier survival analysis indicated that Group A had the best OS rates, whereas Group C had the poorest (Fig. 7H). These findings suggest that CCA patients with low expression levels of both AMDHD1 and SMAD4 are at a higher risk of aggressive disease.

## Discussion

Identifying novel driver genes and elucidating their molecular mechanisms in regulating tumor initiation and progression holds immense potential for developing targeted therapies and improving the prognosis of CCA patients. In this study, we initially discovered a downregulation of AMDHD1 in CCA, which was associated with adverse clinicopathological features and poor prognosis, suggesting that AMDHD1 may serve as a prognostic marker for CCA. We further demonstrated that overexpression of AMDHD1 significantly inhibited proliferation and migration of CCA cells in vitro, and curtailed tumor growth and metastasis in vivo. In addition, AMDHD1 was shown to disrupt G1/S phase progression via the p21/CDK4 pathway and to trigger the apoptotic pathway in CCA cells. Proteomic sequencing analysis underpinned the biological significance of AMDHD1 in CCA, attributing its effects to modulation of the TGF-β signaling pathway.

Accumulating evidence has revealed that TGF-β signaling plays a paradoxical role in oncogenesis, possessing both tumor-promoting and tumor-suppressive functions [12]. The transition of TGF-β signaling from a tumor-suppressive to a tumor-promoting role is considered a critical event in tumorigenesis. In various cancers including CCA, somatic mutations and deletions in genes encoding SMAD proteins and TGF-β receptors are frequent, showing an inverse relationship with patient prognosis [31–33]. Similar to its dual role in other cancers, TGF-β signaling in CCA can either prevent tumor initiation by inhibiting cell proliferation and inducing apoptosis or facilitate tumor progression by enhancing invasion and immune suppression [34]. Further, existing studies have confirmed that epithelial TGF-β signaling impedes CCA development originating from hepatocytes and cholangiocytes by curtailing cholangiocyte proliferation, especially in the context of PTEN deletion [35]. Additionally, TGF-β/SMAD signaling interacts with the Wnt pathway by inhibiting β-catenin phosphorylation and nuclear translocation, thereby reducing CCA proliferation and migration and enhancing the efficacy of Pemigatinib [26]. Hence, exploring the upstream and downstream effectors, as well as the crosstalk mechanisms, is crucial for a comprehensive understanding of the role of TGF-β signaling. In our research, multiple functional enrichment analyses demonstrated that AMDHD1 was involved in the TGF-β signaling pathway along with its co-expressed genes, differentially expressed proteins, and binding partners. Overexpression of AMDHD1 resulted in the upregulation of p21 and PAI-1 and downregulation of c-Myc in CCA cells, signifying a direct enhancement of TGF-β signaling by AMDHD1. Furthermore, the impact of AMDHD1 on the direct targets of TGF-β signaling and the malignant phenotype of CCA cells was abrogated by TGF-β receptor inhibitors, suggesting that AMDHD1 operates in a TGF-β-dependent manner. Considering the supportive role of AMDHD1 in TGF-β signaling, deletions and mutations of AMDHD1 in CCA could impair the tumor-suppressive functions of TGF-β signaling, thereby contributing to tumorigenesis.

AMDHD1, a member of the M38 family of metallopeptidases, encodes a protein with amidohydrolase domains, traditionally associated with protein binding and involvement in proteolytic processes [36]. To date, direct evidence towards AMDHD1’s protein-binding capabilities and its regulatory impact on biological functions remains scarce. In this study, we utilized IP-MS analysis to demonstrate the direct binding of AMDHD1 to proteins. We identified 5670 AMDHD1-binding peptides and 614 potential AMDHD1-bound proteins. Notably, we pinpointed SMAD4 and SMAD2 as the prospective downstream effectors of AMDHD1 within TGF-β signaling pathway. In cholangiocytes, SMAD4 is crucial in restraining the proliferative response to injury and curtailing CCA development [37]. It has been established that ubiquitination and subsequent proteasomal degradation of SMAD4 facilitate tumor progression by endorsing EMT and cell migration in PAAD and colorectal cancer [27, 38]. Consistent with previous findings, our data confirmed that AMDHD1 interacted with SMAD4 via the MH2 domain. This interaction impeded SMAD4 ubiquitination and degradation, thereby elevating SMAD4 levels and diminishing the viability and migration of CCA cells. The suppression effect of AMDHD1 overexpression on TGF-β targets, EMT pathway and the malignant phenotype of CCA cells was abrogated upon SMAD4 deletion. Furthermore, AMDHD1 was shown to bind to pSMAD2/3, enhancing their phosphorylation and synergistically promoting the nuclear translocation of pSMAD2/3, thereby activating TGF-β signaling. Phosphorylation at different SMAD sites plays distinct roles in TGF-β signaling [39, 40]. Specifically, phosphorylation of SMAD4 at Thr276 enhances nuclear accumulation and transcriptional activity [39], whereas phosphorylation at Thr197 promotes ubiquitination and degradation [27]. Similarly, while TGF-β-induced COOH-terminal pSMAD2/3 (pSMAD2/3 C) inhibits cell viability, cytokine-mediated linker pSMAD2/3 (pSMAD2/3 L) encourages cell proliferation and hepatocarcinogenesis [41]. Our findings illustrated that AMDHD1-mediated pSMAD2/3 was crucial for their interaction, highlighting the importance of further research into the specific phosphorylation sites on pSMAD2/3 influenced by AMDHD1 and their roles in TGF-β signaling. Taken together, we proposed a regulatory model wherein AMDHD1 inhibited SMAD4 degradation through MH2 domain interaction and concurrently promoted SMAD2/3 phosphorylation, thus activating TGF-β signaling and influencing cell cycle, apoptosis and invasion dynamics in CCA (Fig. 7H). Intriguingly, we established that AMDHD1 could bind simultaneously to SMAD4 and SMAD2/3, leading to future investigations into whether AMDHD1 acts as a scaffold protein, facilitating the integration and nuclear translocation of SMAD proteins. Finally, we developed a prognostic stratification based on the expression levels of AMDHD1 and SMAD4, revealing that CCA patients with low levels of both AMDHD1 and SMAD4 exhibited the poorest OS. These results suggested that a combined analysis of AMDHD1 and SMAD4 expression could serve as a valuable tool for identifying high-risk and aggressive forms of CCA. One limitation of our findings was that the prognostic value of AMDHD1 and SMAD4 had not been validated in prospective studies. Another issue that was not addressed in the study was the lack of explorations on preclinical application. Future studies may involve developing a treatment strategy of forced expression of AMDHD1 via exosomes or nanoprobe-mediated delivery system in a pre-clinical CCA model.

## Conclusion

In conclusion, our study elucidates a novel regulatory mechanism wherein AMDHD1 impedes the ubiquitin-mediated degradation of SMAD4 and collaboratively enhances the phosphorylation of SMAD2/3, which activates TGF-β signaling pathway and lead to the suppression of growth and metastasis in CCA cells. Our findings position AMDHD1 as a pivotal prognostic marker and tumor suppressor, offering a novel therapeutic avenue through the combined targeting of AMDHD1 and SMAD4 in CCA.

## Supplementary information

Supplementary Tables and Figures

Western Blot Raw Data

## Data Availability

The original contributions presented in the study are included in the article or the supplementary material. This paper does not report original code. Other data that support the findings are available from the corresponding author upon reasonable request.

## References

[1] Valle JW, Kelley RK, Nervi B, Oh DY, Zhu AX. Biliary tract cancer. Lancet. 2021;397:428–44. 33516341 10.1016/S0140-6736(21)00153-7

[2] Banales JM, Marin J, Lamarca A, Rodrigues PM, Khan SA, Roberts LR, et al. Cholangiocarcinoma 2020: the next horizon in mechanisms and management. Nat Rev Gastroenterol Hepatol. 2020;17:557–88. 32606456 10.1038/s41575-020-0310-z PMC7447603

[3] Valle J, Wasan H, Palmer DH, Cunningham D, Anthoney A, Maraveyas A, et al. Cisplatin plus gemcitabine versus gemcitabine for biliary tract cancer. N. Engl J Med. 2010;362:1273–81. 20375404 10.1056/NEJMoa0908721

[4] Kam AE, Masood A, Shroff RT. Current and emerging therapies for advanced biliary tract cancers. Lancet Gastroenterol Hepatol. 2021;6:956–69. 34626563 10.1016/S2468-1253(21)00171-0

[5] Jiang X, O’Reilly PF, Aschard H, Hsu YH, Richards JB, Dupuis J, et al. Genome-wide association study in 79,366 European-ancestry individuals informs the genetic architecture of 25-hydroxyvitamin D levels. Nat Commun. 2018;9:260. 29343764 10.1038/s41467-017-02662-2 PMC5772647

[6] Assié G, Guillaud-Bataille M, Ragazzon B, Bertagna X, Bertherat J, Clauser E. The pathophysiology, diagnosis and prognosis of adrenocortical tumors revisited by transcriptome analyses. Trends Endocrinol Metab. 2010;21:325–34. 20097573 10.1016/j.tem.2009.12.009

[7] Song Y, Ahn J, Suh Y, Davis ME, Lee K. Identification of novel tissue-specific genes by analysis of microarray databases: a human and mouse model. PLoS ONE. 2013;8:e64483. 23741331 10.1371/journal.pone.0064483 PMC3669334

[8] Zhang Q, Xiao Z, Sun S, Wang K, Qian J, Cui Z, et al. Integrated proteomics and bioinformatics to identify potential prognostic biomarkers in hepatocellular carcinoma. Cancer Manag Res. 2021;13:2307–17. 33732023 10.2147/CMAR.S291811 PMC7959210

[9] Wang H, Zhao L, Liu H, Luo S, Akinyemiju T, Hwang S, et al. Variants in SNAI1, AMDHD1 and CUBN in vitamin D pathway genes are associated with breast cancer risk: a large-scale analysis of 14 GWASs in the DRIVE study. Am J Cancer Res. 2020;10:2160–73. 32775008 PMC7407344

[10] Shi Y, Massagué J. Mechanisms of TGF-beta signaling from cell membrane to the nucleus. Cell. 2003;113:685–700. 12809600 10.1016/s0092-8674(03)00432-x

[11] Attisano L, Wrana JL. Signal transduction by the TGF-beta superfamily. Science. 2002;296:1646–7. 12040180 10.1126/science.1071809

[12] Zhao M, Mishra L, Deng CX. The role of TGF-β/SMAD4 signaling in cancer. Int J Biol Sci. 2018;14:111–23. 29483830 10.7150/ijbs.23230 PMC5821033

[13] Tang Z, Li C, Kang B, Gao G, Li C, Zhang Z. GEPIA: a web server for cancer and normal gene expression profiling and interactive analyses. Nucleic Acids Res. 2017;45:W98–102. 28407145 10.1093/nar/gkx247 PMC5570223

[14] Gao J, Aksoy BA, Dogrusoz U, Dresdner G, Gross B, Sumer SO, et al. Integrative analysis of complex cancer genomics and clinical profiles using the cBioPortal. Sci Signal. 2013;6:pl1. 23550210 10.1126/scisignal.2004088 PMC4160307

[15] Ma Z, Li Z, Wang S, Zhou Z, Liu C, Zhuang H, et al. ZMAT1 acts as a tumor suppressor in pancreatic ductal adenocarcinoma by inducing SIRT3/p53 signaling pathway. J Exp Clin Cancer Res. 2022;41:130. 35392973 10.1186/s13046-022-02310-8 PMC8988381

[16] von Mering C, Jensen LJ, Snel B, Hooper SD, Krupp M, Foglierini M, et al. STRING: known and predicted protein-protein associations, integrated and transferred across organisms. Nucleic Acids Res. 2005;33:D433–7. 15608232 10.1093/nar/gki005 PMC539959

[17] Camp RL, Dolled-Filhart M, Rimm DL. X-tile: a new bio-informatics tool for biomarker assessment and outcome-based cut-point optimization. Clin Cancer Res. 2004;10:7252–9. 15534099 10.1158/1078-0432.CCR-04-0713

[18] Ma J, Chen T, Wu S, Yang C, Bai M, Shu K, et al. iProX: an integrated proteome resource. Nucleic Acids Res. 2019;47:D1211–7. 30252093 10.1093/nar/gky869 PMC6323926

[19] Jirawatnotai S, Hu Y, Livingston DM, Sicinski P. Proteomic identification of a direct role for cyclin d1 in DNA damage repair. Cancer Res. 2012;72:4289–93. 22915759 10.1158/0008-5472.CAN-11-3549 PMC3432743

[20] Datto MB, Li Y, Panus JF, Howe DJ, Xiong Y, Wang XF. Transforming growth factor beta induces the cyclin-dependent kinase inhibitor p21 through a p53-independent mechanism. Proc Natl Acad Sci USA. 1995;92:5545–9. 7777546 10.1073/pnas.92.12.5545 PMC41732

[21] Dennler S, Itoh S, Vivien D, Ten DP, Huet S, Gauthier JM. Direct binding of Smad3 and Smad4 to critical TGF beta-inducible elements in the promoter of human plasminogen activator inhibitor-type 1 gene. EMBO J. 1998;17:3091–100. 9606191 10.1093/emboj/17.11.3091 PMC1170648

[22] Warner BJ, Blain SW, Seoane J, Massagué J. Myc downregulation by transforming growth factor beta required for activation of the p15(Ink4b) G(1) arrest pathway. Mol Cell Biol. 1999;19:5913–22. 10454538 10.1128/mcb.19.9.5913 PMC84444

[23] Yuan B, Liu J, Cao J, Yu Y, Zhang H, Wang F, et al. PTPN3 acts as a tumor suppressor and boosts TGF-β signaling independent of its phosphatase activity. EMBO J. 2019;38:e99945. 31304624 10.15252/embj.201899945 PMC6627230

[24] Laping NJ, Grygielko E, Mathur A, Butter S, Bomberger J, Tweed C, et al. Inhibition of transforming growth factor (TGF)-beta1-induced extracellular matrix with a novel inhibitor of the TGF-beta type I receptor kinase activity: SB-431542. Mol Pharm. 2002;62:58–64. 10.1124/mol.62.1.58 12065755

[25] Willems E, Cabral-Teixeira J, Schade D, Cai W, Reeves P, Bushway PJ, et al. Small molecule-mediated TGF-β type II receptor degradation promotes cardiomyogenesis in embryonic stem cells. Cell Stem Cell. 2012;11:242–52. 22862949 10.1016/j.stem.2012.04.025 PMC3419596

[26] Liu J, Ren G, Li K, Liu Z, Wang Y, Chen T, et al. The Smad4-MYO18A-PP1A complex regulates β-catenin phosphorylation and pemigatinib resistance by inhibiting PAK1 in cholangiocarcinoma. Cell Death Differ. 2022;29:818–31. 34799729 10.1038/s41418-021-00897-7 PMC8990017

[27] Gao P, Hao JL, Xie QW, Han GQ, Xu BB, Hu H, et al. PELO facilitates PLK1-induced the ubiquitination and degradation of Smad4 and promotes the progression of prostate cancer. Oncogene. 2022;41:2945–57. 35437307 10.1038/s41388-022-02316-8

[28] Wu N, Jiang M, Liu H, Chu Y, Wang D, Cao J, et al. LINC00941 promotes CRC metastasis through preventing SMAD4 protein degradation and activating the TGF-β/SMAD2/3 signaling pathway. Cell Death Differ. 2021;28:219–32. 32737443 10.1038/s41418-020-0596-y PMC7853066

[29] Melisi D, Ishiyama S, Sclabas GM, Fleming JB, Xia Q, Tortora G, et al. LY2109761, a novel transforming growth factor beta receptor type I and type II dual inhibitor, as a therapeutic approach to suppressing pancreatic cancer metastasis. Mol Cancer Ther. 2008;7:829–40. 18413796 10.1158/1535-7163.MCT-07-0337 PMC3088432

[30] Abdollah S, Macías-Silva M, Tsukazaki T, Hayashi H, Attisano L, Wrana JL. TbetaRI phosphorylation of Smad2 on Ser465 and Ser467 is required for Smad2-Smad4 complex formation and signaling. J Biol Chem. 1997;272:27678–85. 9346908 10.1074/jbc.272.44.27678

[31] Ong CK, Subimerb C, Pairojkul C, Wongkham S, Cutcutache I, Yu W, et al. Exome sequencing of liver fluke-associated cholangiocarcinoma. Nat Genet. 2012;44:690–3. 22561520 10.1038/ng.2273

[32] Jiao Y, Pawlik TM, Anders RA, Selaru FM, Streppel MM, Lucas DJ, et al. Exome sequencing identifies frequent inactivating mutations in BAP1, ARID1A and PBRM1 in intrahepatic cholangiocarcinomas. Nat Genet. 2013;45:1470–3. 24185509 10.1038/ng.2813 PMC4013720

[33] Argani P, Shaukat A, Kaushal M, Wilentz RE, Su GH, Sohn TA, et al. Differing rates of loss of DPC4 expression and of p53 overexpression among carcinomas of the proximal and distal bile ducts. Cancer. 2001;91:1332–41. 11283934

[34] Papoutsoglou P, Louis C, Coulouarn C. Transforming Growth Factor-Beta (TGFβ) signaling pathway in cholangiocarcinoma. Cells. 2019;8:960. 10.3390/cells8090960 PMC6770250 31450767

[35] Mu X, Pradere JP, Affò S, Dapito DH, Friedman R, Lefkovitch JH, et al. Epithelial transforming growth factor-β signaling does not contribute to liver fibrosis but protects mice from cholangiocarcinoma. Gastroenterology. 2016;150:720–33. 26627606 10.1053/j.gastro.2015.11.039 PMC6490681

[36] Seibert CM, Raushel FM. Structural and catalytic diversity within the amidohydrolase superfamily. Biochemistry. 2005;44:6383–91. 15850372 10.1021/bi047326v

[37] Guo B, Friedland SC, Alexander W, Myers JA, Wang W, O’Dell MR, et al. Arid1a mutation suppresses TGF-β signaling and induces cholangiocarcinoma. Cell Rep. 2022;40:111253. 36044839 10.1016/j.celrep.2022.111253 PMC9808599

[38] Liang Q, Tang C, Tang M, Zhang Q, Gao Y, Ge Z. TRIM47 is up-regulated in colorectal cancer, promoting ubiquitination and degradation of SMAD4. J Exp Clin Cancer Res. 2019;38:159. 30979374 10.1186/s13046-019-1143-x PMC6461818

[39] Roelen BA, Cohen OS, Raychowdhury MK, Chadee DN, Zhang Y, Kyriakis JM, et al. Phosphorylation of threonine 276 in Smad4 is involved in transforming growth factor-beta-induced nuclear accumulation. Am J Physiol Cell Physiol. 2003;285:C823–30. 12801888 10.1152/ajpcell.00053.2003

[40] Demagny H, Araki T, De Robertis EM. The tumor suppressor Smad4/DPC4 is regulated by phosphorylations that integrate FGF, Wnt, and TGF-β signaling. Cell Rep. 2014;9:688–700. 25373906 10.1016/j.celrep.2014.09.020

[41] Yoshida K, Matsuzaki K, Murata M, Yamaguchi T, Suwa K, Okazaki K. Clinico-pathological importance of TGF-β/Phospho-Smad signaling during human hepatic fibrocarcinogenesis. Cancers. 2018;10:183. 10.3390/cancers10060183 PMC6025395 29874844

