# Supplementary material for: AMDHD1 acts as a tumor suppressor and contributes to activation of TGF-β signaling pathway in cholangiocarcinoma
Source: Cell Death Differ. 2024 Aug 14;32(1):162–76. doi: 10.1038/s41418-024-01361-y (PMC11742690; doi:10.1038/s41418-024-01361-y)
Supplement: Supplementary file 1 — Supplementary Tables and Figures [file 41418_2024_1361_MOESM1_ESM.pdf]

AMDHD1 acts as a tumor suppressor and contributes to activation of TGF-β signaling pathway in cholangiocarcinoma

Authors: Zuyi Ma, Jia Sun, Zhenchong Li, Shanzhou Huang, Binglu Li

Table of contents

Supplementary tables..2

Supplementary figures..8

Supplementary Tables

Supplementary Table 1. The antibodies and agents used in the study.

Antibody/Agent	Assay	Catalog	Origin	Dilution/ Concentration	Incubation period
AMDHD1	WB	NBP1-82701	Novus Biologicals (Centennial, USA)	1/1000	overnight, 4°C
	IHC			1/400	overnight, 4°C
	IF			1/500	overnight, 4°C
	IP			1/100	overnight, 4°C
N-Cadherin	WB	CY5015	Abways Technology (Shanghai, China)	1/2000	overnight, 4°C
BID	WB	CY6785		1/1000	overnight, 4°C
BAX	WB	CY5059		1/1000	overnight, 4°C
Bcl-2	WB	CY6717		1/1000	overnight, 4°C
p21	WB	CY5543		1/1000	overnight, 4°C
CDK4	WB	CY5836		1/1000	overnight, 4°C
Cyclin D1	WB	CY5404		1/2000	overnight, 4°C
PAI-1	WB	CY6901		1/500	overnight, 4°C
c-Myc	WB	CY5150		1/5000	overnight, 4°C
TGFB1	WB	CY6608		1/1000	overnight, 4°C
TGFBR1	WB	CY2905		1/1000	overnight, 4°C
TGFB2	WB	CY1266		1/1000	overnight, 4°C
TGFBR2	WB	CY6879		1/1000	overnight, 4°C
SMAD4	WB	#38454	Cell Signaling Technology (Danvers, USA)	1/1000	overnight, 4°C
	IHC			1/200	overnight, 4°C
	IF			1/500	overnight, 4°C
	IP			1/100	overnight, 4°C
SMAD2/3	WB	#8685		1/1000	overnight, 4°C
	IF			1/500	overnight, 4°C
	IP			1/100	overnight, 4°C
pSMAD2	WB	#3108		1/1000	overnight, 4°C
GAPDH	WB	#5174		1/1000	overnight, 4°C
Rabbit IgG, HRP	WB	#7074		1/2000	1h, 25°C
Mouse IgG, HRP	WB	#7076		1/2000	1h, 25°C
pSMAD3	WB	ab52903	Abcam (Cambridge, UK)	1/1000	overnight, 4°C
Cleaved Caspase-3	WB	ab32042		1/1000	overnight, 4°C
Snail	WB	TA500366S	OriGene Technologies (Rockville, USA)	1/1000	overnight, 4°C

E-Cadherin	WB	CQA8367	Cohesion Biosciences (London, UK)	1/1000	overnight, 4°C
Ubiquitin	WB	A19686	ABclonal Technology (Wuhan, China)	1/500	overnight, 4°C
TGF-β cytokine		HY-P7118	MedChemExpress (Monmouth Junction, USA)	0.2ng/mL	1h, 37°C
SB431524	Functional experiments	S1067	Selleck Chemicals (Houston, USA)	10uM	1h, 37°C
ITD-1		S6713		3uM	1h, 37°C
LY2109761		S2704		2uM	24h, 37°C
Cycloheximide		S7418		10uM	4h, 37°C
MG132		S2619		10uM	24h, 37°C

Supplementary Table 2. Sequences of the oligonucleotides for shRNA and Real-time quantitative PCR.

Assay	Target	Sequence (5'- 3')
shRNA	AMDHD1	GCACACACACATCCAGTATGG
		GGAAGAAGTGAGTGATGAAGG
	SMAD4	GCCATAGTGAAGGACTGTTGC
		GGACTGTTGCAGATAGCATCA
RT-qPCR	AMDHD1	CAGGAGCCACCTACATGGAAA
		CATATCCACTCTTGCACTCCAC
	SMAD4	ACGAACGAGTTGTATCACCTGG
		TGCACGATTACTTGGTGGATG
	CDKN1A	TGTCCGTCAGAACCCATGC
		AAAGTCGAAGTTCCATCGCTC
	SERPINE1	ACCGCAACGTGGTTTTCTCA
		TTGAATCCCATAGCTGCTTGAAT
	c-Myc	GGCTCCTGGCAAAAGGTCA
		CTGCGTAGTTGTGCTGATGT

Supplementary Table 3. Correlation between AMDHD1 expression with clinicopathological characteristics of cholangiocarcinoma patients.

Clinicopathological variables	Patients(n=108)	AMDHD1 expression		P Value
		High (56)	Low (52)	
Gender				
Male	68	33	35	0.483
Female	40	23	17	
Age				
≧60	70	38	32	0.627
<60	38	18	20	
Differentiation				
Poor	60	38	22	**<0.05**
Well	48	18	30	
Lymphnodes metastasis				
Positive	42	13	29	0.963
Negative	66	22	44	
Perineural invasion				
Positive	54	34	20	**<0.05**
Negative	54	22	32	
CA19-9				
≧200 ng/ml	65	41	24	**<0.01**
<200 ng/ml	43	15	28	
TNM stage				
Advanced (III & IV)	48	32	16	**<0.01**
Early (I & II)	60	24	36	

Supplementary Table 4. Univariate and multivariate Cox regression analysis of risk factors associated with overall survival.

Clinicopathological variables	Univariate analysis			Multivariate analysis		
	HR	95% CI	P Value	HR	95% CI	P Value
AMDHD1 expression (Low vs. High)	5.42	4.54-6.30	**<0.01**	4.01	3.21-4.81	**<0.01**
Gender (Male vs. Female)	1.58	0.73-2.43	0.43			
Age (≧60 vs. <60)	1.18	0.46-1.90	0.71			
Differentiation (Poor vs. Well)	2.06	1.22-2.90	**<0.05**	1.61	0.72-2.50	0.46
Lymphnodes metastasis (Positive vs. Negative)	1.86	0.94-2.78	0.07			
Perineural invasion (Positive vs. Negative)	2.75	1.92-3.58	**<0.01**	1.94	1.15-2.73	**<0.05**
CA19-9 (≧200 ng/ml vs. <200 ng/ml)	2.81	2.08-3.54	**<0.01**	2.12	1.35-2.89	**<0.05**
TNM stage (Advanced vs. Early)	4.02	2.89-5.15	**<0.01**	2.98	2.03-3.93	**<0.01**

Supplementary Table 5. Univariate and multivariate Cox regression analysis of risk factors associated with disease-free survival.

Clinicopathological variables	Univariate analysis			Multivariate analysis		
	HR	95% CI	P Value	HR	95% CI	P Value
AMDHD1 expression (Low vs. High)	5.01	4.11-5.91	**<0.01**	3.25	2.23-4.27	**<0.01**
Gender (Male vs. Female)	1.21	0.46-1.96	0.74			
Age (≧60 vs. <60)	1.12	0.31-1.93	0.83			
Differentiation (Poor vs. Well)	2.47	1.56-3.38	**<0.05**	1.73	0.92-3.22	0.08
Lymphnodes metastasis (Positive vs. Negative)	2.26	1.31-3.21	**<0.05**	1.51	0.49-2.53	0.63
Perineural invasion (Positive vs. Negative)	1.32	0.69-1.95	0.49			
CA19-9 (≧200 ng/ml vs. <200 ng/ml)	2.52	1.72-3.32	**<0.01**	2.16	1.19-3.13	**<0.05**
TNM stage (Advanced vs. Early)	3.89	2.92-4.86	**<0.01**	2.52	1.79-3.25	**<0.01**

Supplementary Figures

Supplementary Figure 1. AMDHD1 expressions in pan-cancer.

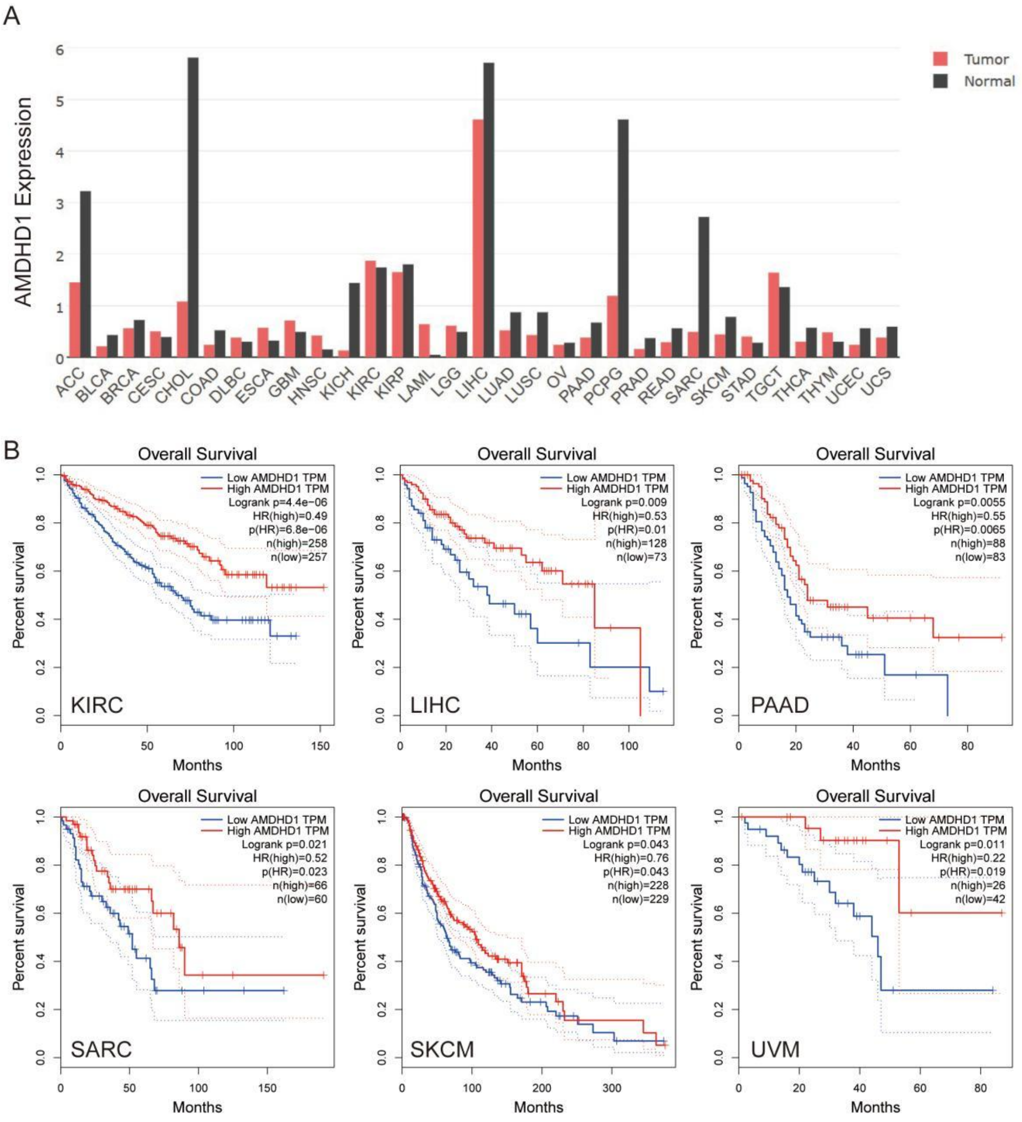

Supplementary Figure 1. A The analyses of AMDHD1 expression in several cancer types in GEPIA database. **B** Kaplan-Meier analyses showed patients with low AMDHD1 expression had inferior overall survival in kidney renal clear cell carcinoma (KIRC), liver hepatocellular carcinoma (LIHC), pancreatic adenocarcinoma (PAAD), sarcoma (SARC), skin cutaneous melanoma (SKCM) and uveal melanoma (UVM). Kaplan-Meier analyses and log-rank tests were performed in B.

Supplementary Figure 2. The AMDHD1 expressions and morphology of the used cell lines.

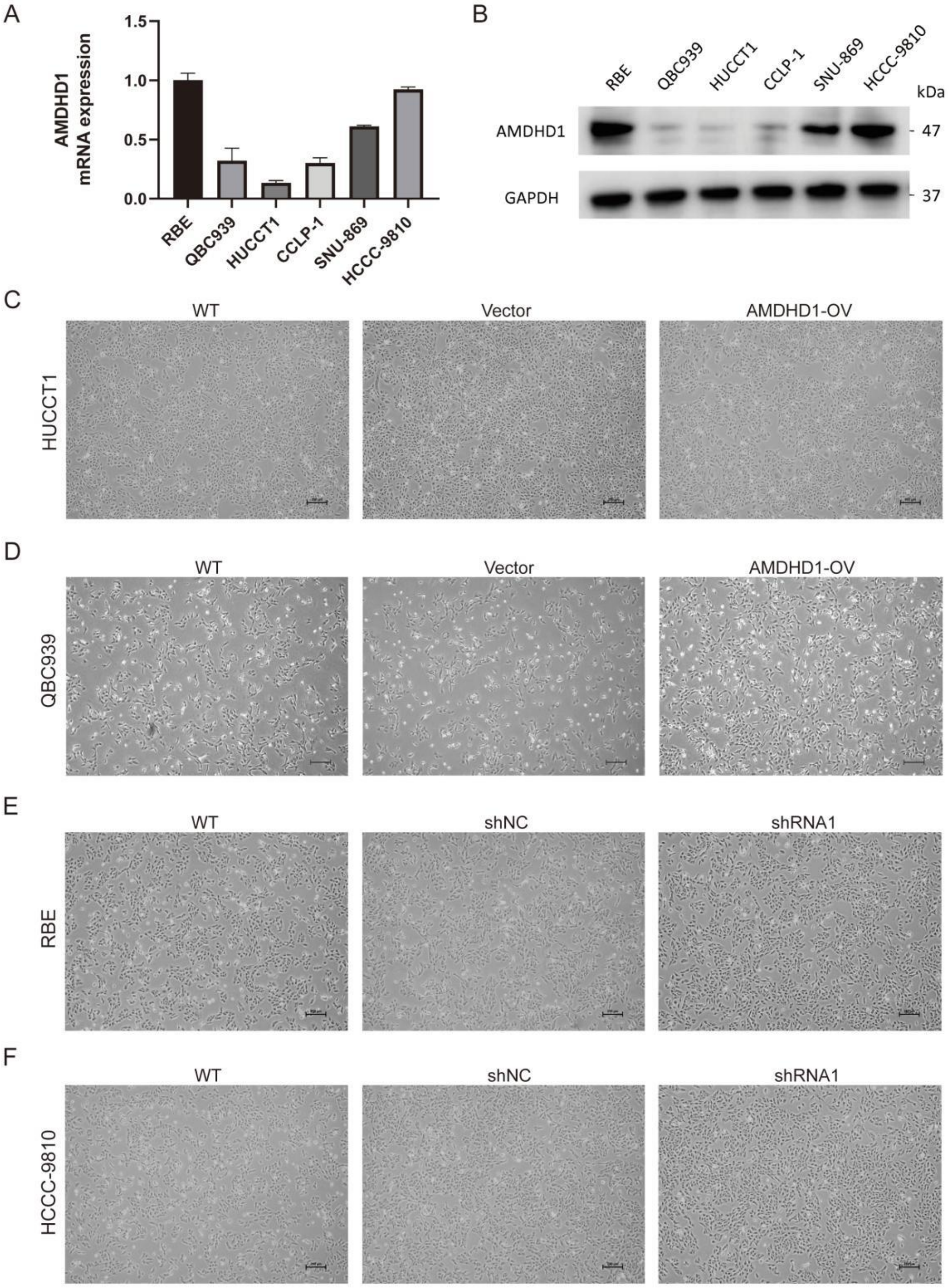

Supplementary Figure 2. A-B Real-time quantitative polymerase chain reaction (**A**) and western blot (**B**) were used to detect the mRNA and protein expressions of AMDHD1 among CCA cell lines. **C-F** The cell morphology of HUCCT1 (**C**), QBC939 (**D**), RBE (**E**) and HCCC-9810 (**F**) cells before and after transfection. Scale bars: 250 μm.

Supplementary Figure 3. AMDHD1 inhibits the proliferation and migration of cholangiocarcinoma (CCA) cells.

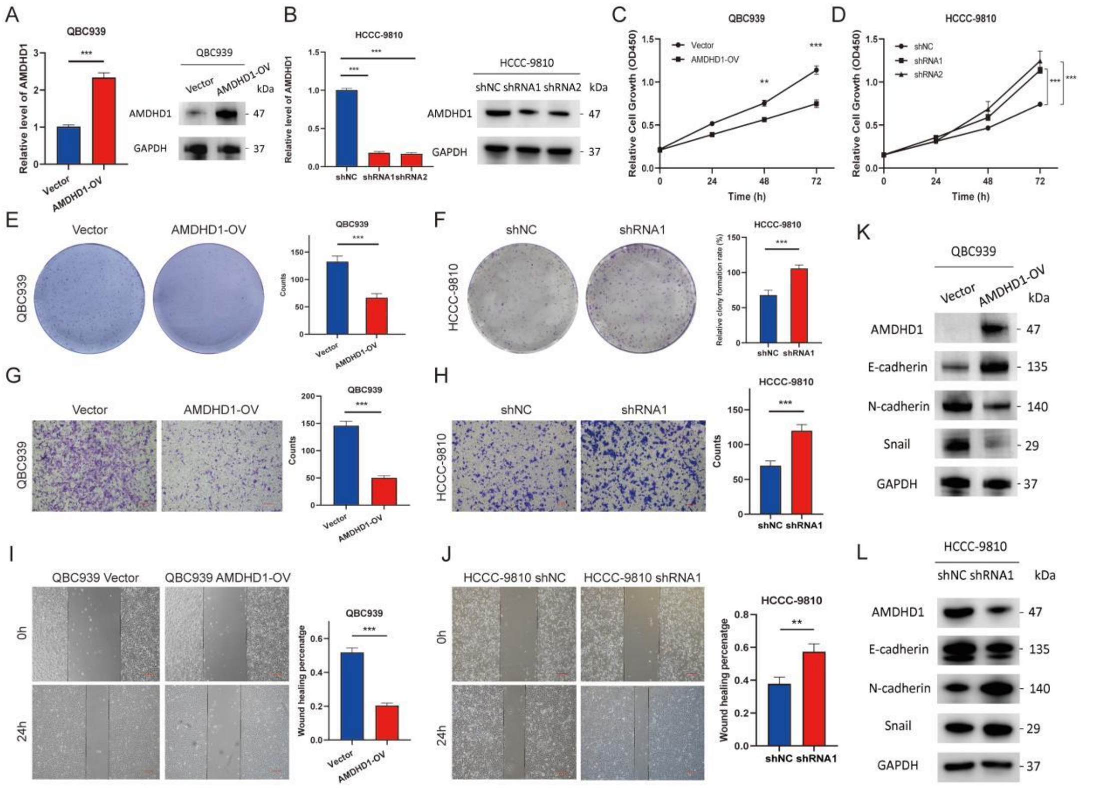

Supplementary Figure 3. A-B RT-qPCR and western blot were used to detect the over-expression or knockdown of AMDHD1 in QBC939 (**A**) and HCCC-9810 cells (**B**). **C-D** CCK-8 assays were used to detect the proliferation of QBC939/AMDHD1-OV (**C**) and HCCC-9810/AMDHD1-KD cells (**D**). **E-F** Colony formation assays were used to detect the proliferation of QBC939/AMDHD1-OV (**E**) and HCCC-9810/AMDHD1-KD cells (**F**). **G-H** Transwell assays were used to detect the migration of QBC939/AMDHD1-OV (**G**) and HCCC-9810/AMDHD1-KD cells (**H**). **I-J** Wound-healing assays were used to detect the migration of QBC939/AMDHD1-OV (**I**) and HCCC-9810/AMDHD1-KD cells (**J**). **K-L** Western blot analysis of protein levels of AMDHD1, E-cadherin, N-cadherin and Snail in QBC939/AMDHD1-OV (**K**) and HCCC-9810/AMDHD1-KD cells (**L**). All ns not significant, ** P-value <0.01, *** P-value <0.001. Scale bars in G and H: 100 μm. Scale bars in I-J: 250 μm. P-values were assessed using two-tailed t-tests and ANOVA followed by Dunnett's tests for multiple comparison in A-J.

Supplementary Figure 4. AMDHD1 promotes apoptosis and arrests cell cycle of cholangiocarcinoma (CCA) cells.

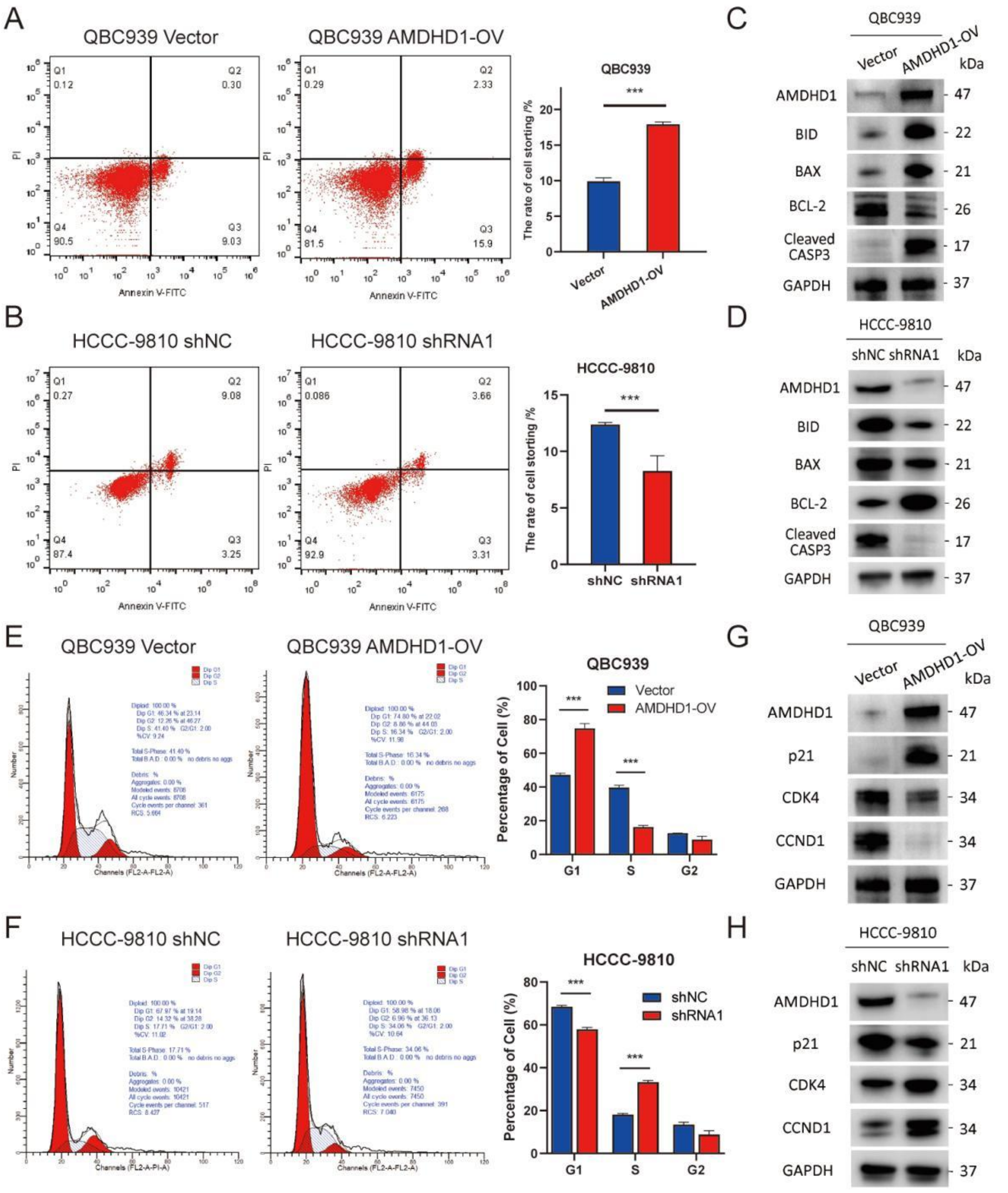

Supplementary Figure 4. A-B Flow cytometry was used to detect the percentage of apoptotic cells in QBC939/AMDHD1-OV (**A**) and HCCC-9810/AMDHD1-KD cells (**B**). **C-D** Western blot analysis of protein levels of AMDHD1, BAX, cleaved CASP3, BID and BCL-2 in QBC939/AMDHD1-OV (**C**) and HCCC-9810/AMDHD1-KD cells (**D**). **E-F** Flow cytometry was used to detect the cell cycle of QBC939/AMDHD1-OV (**E**) and HCCC-9810/AMDHD1-KD cells (**F**). **G-H** Western blot analysis of protein levels of AMDHD1, p21, CDK4 and CCND1 in

QBC939/AMDHD1-OV (**G**) and HCCC-9810/AMDHD1-KD cells (**H**). All *** P-value <0.001. P-values were assessed using two-tailed t-tests in A, B, E and F.

Supplementary Figure 5. Identification of AMDHD1 downstream effectors in TGF-β pathway.

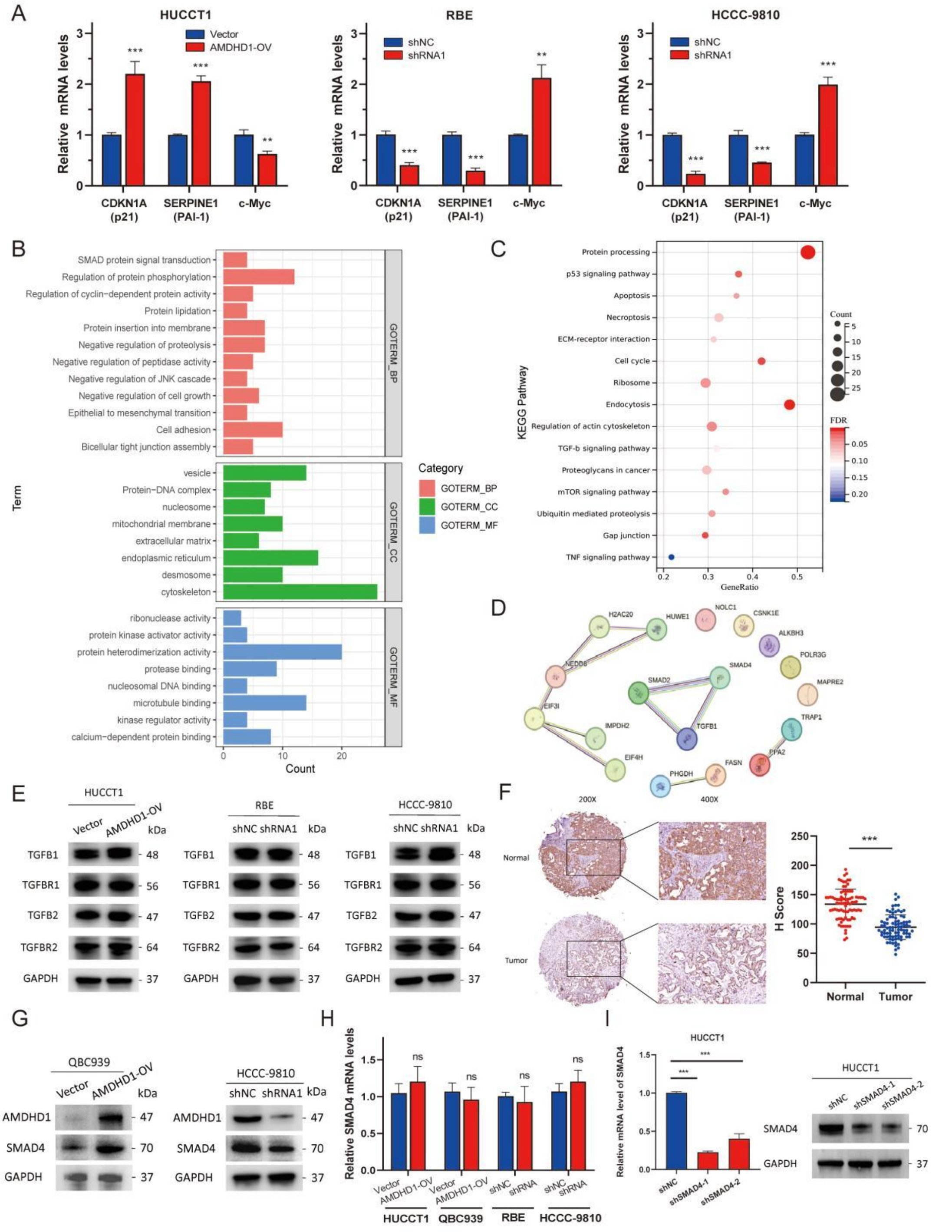

Supplementary Figure 5. A Real-time quantitative polymerase chain reaction (RT-qPCR) were used to detect the mRNA expressions of CDKN1A (p21), SERPINE1 (PAI-1) and c-Myc in AMDHD1-OV and AMDHD1-KD cells. **B-C** Gene Ontology (**B**) and Kyoto Encyclopedia of Genes and Genomes pathway (**C**)

enrichment analyses of AMDHD1-bound proteins based on immunoprecipitation mass spectrometry. **D** STRING functional enrichment analysis on 17 overlapping proteins. **E** Western blot analysis of protein levels of TGFB1, TGFB2, TGFBR1 and TGFBR2 in AMDHD1-OV and AMDHD1-KD cells. **F** Representative images of SMAD4 staining in 96 CCA specimens and normal bile duct tissues and immunohistochemistry staining score showed the down-regulation of SMAD4 protein levels in CCA tissues. **G** Western blot were used to detect the protein expressions of SMAD4 in AMDHD1-OV and AMDHD1-KD cells. **H** RT-qPCR were used to detect the mRNA expressions of SMAD4 in AMDHD1-OV and AMDHD1-KD cells. **I** RT-qPCR and western blot were used to detect the knockdown of SMAD4 in HUCCT1 cells. All ns not significant, ** P-value <0.01, *** P-value <0.001. P-values were assessed using two-tailed t-tests in A, F, H and I.

Supplementary Figure 6. SMAD4 is essential for the regulation of AMDHD1 on the malignant phenotype of cholangiocarcinoma (CCA) cells.

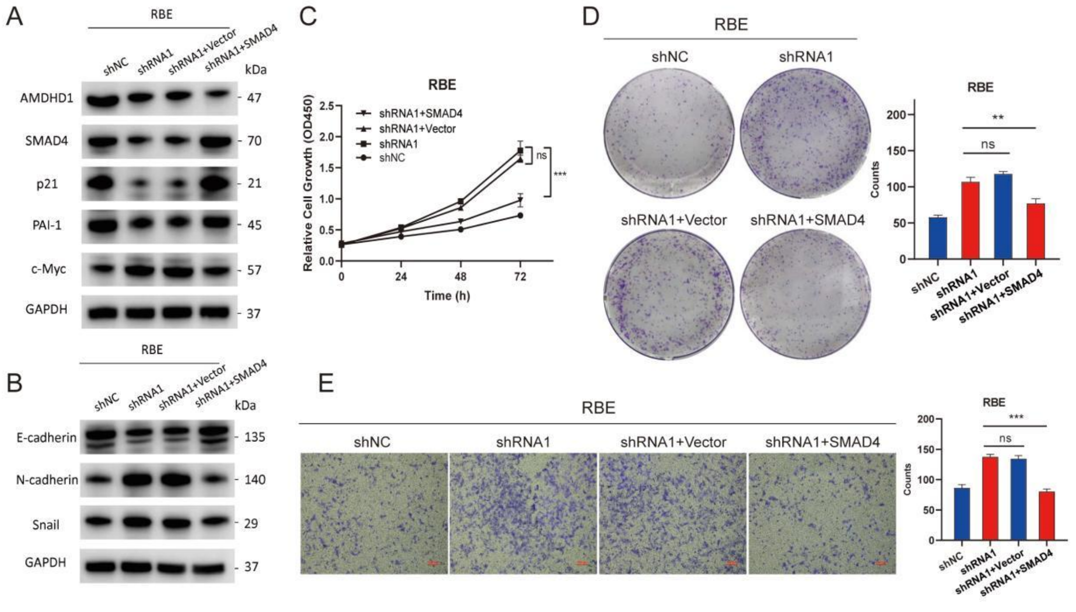

Supplementary Figure 6. A-B RBE/AMDHD1-KD cells were transfected with SMAD4 vector and the protein levels of AMDHD1, p21, PAI-1, c-Myc, E-cadherin, N-cadherin and Snail were analyzed by immunoblotting. **C-E** CCK-8 assays (**C**), colony formation assays (**D**) and transwell assays (**E**) were performed in RBE/AMDHD1-KD cells transfected with SMAD4 vector. All ns not significant, ** P-value <0.01, *** P-value <0.001. Scale bars: 100 μm. P-values were assessed using two-tailed t-tests in C, D and E.
