# Supplementary material for: AMDHD1 acts as a tumor suppressor and contributes to activation of TGF-β signaling pathway in cholangiocarcinoma
Source: Cell Death Differ. 2024 Aug 14;32(1):162–76. doi: 10.1038/s41418-024-01361-y (PMC11742690; doi:10.1038/s41418-024-01361-y)
Supplement: Supplementary file 2 — Western Blot Raw Data [file 41418_2024_1361_MOESM2_ESM.pdf]

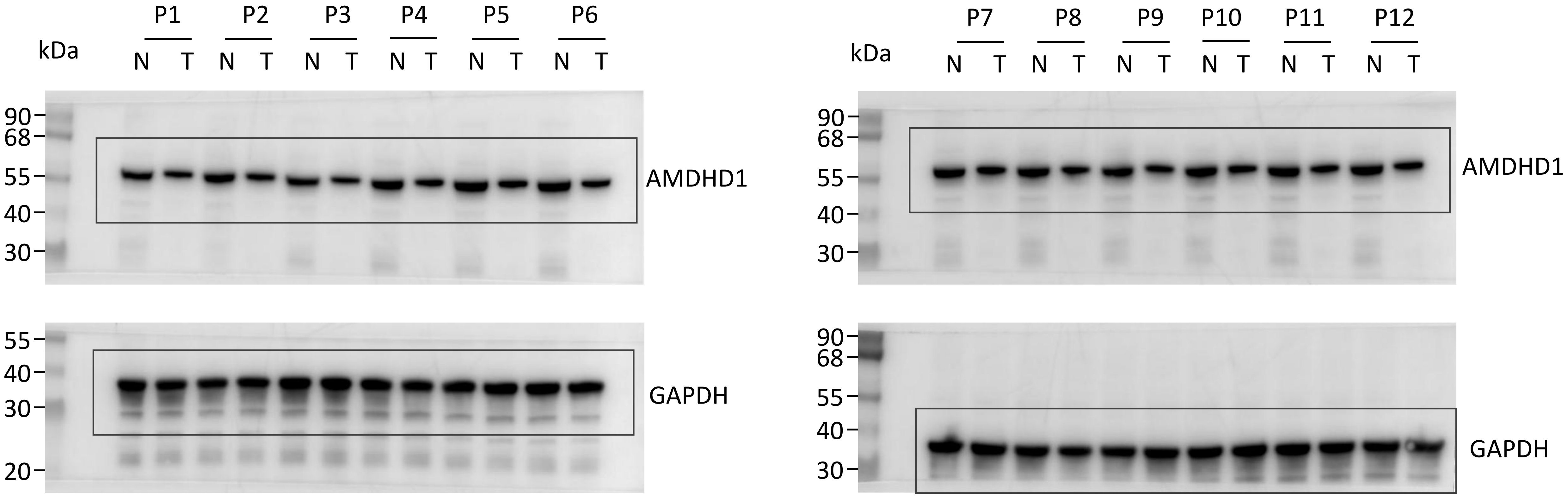
P1
P2
P3
P4
P5
P6
kDa
N T N T N T N T N T N T
90
68
55
40
30
AMDHD1
55
40
30
20
GAPDH
P7
P8
P9
P10
P11
P12
kDa
N T N T N T N T N T N T
90
68
55
40
30
AMDHD1
90
68
55
40
30
GAPDH

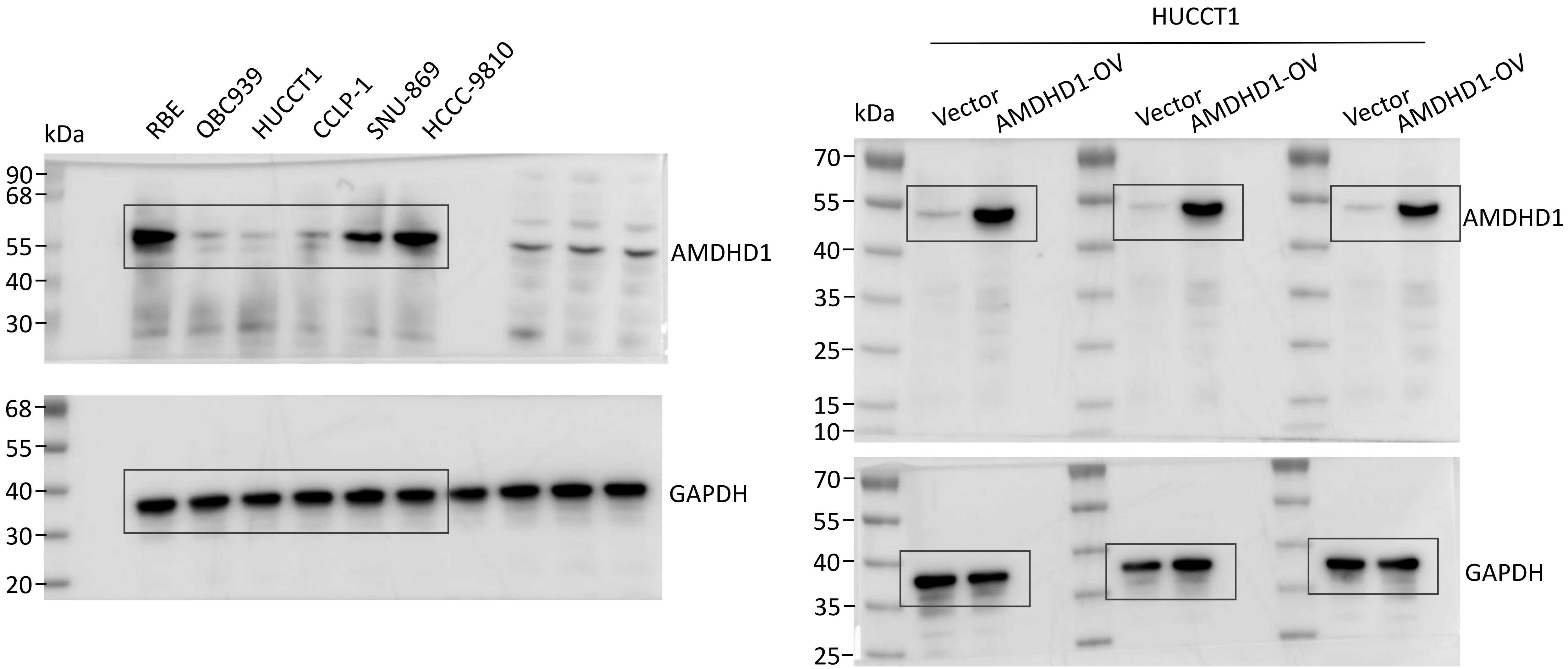
kDa
RBE
QBC939
HUCCT1
CCLP-1
SNU-869
HCCC-9810
90
68
55
40
30
AMDHD1
68
55
40
30
20
GAPDH
HUCCT1
kDa
Vector
AMDHD1-OV
Vector
AMDHD1-OV
Vector
AMDHD1-OV
70
55
40
35
25
15
10
AMDHD1
70
55
40
35
25
GAPDH

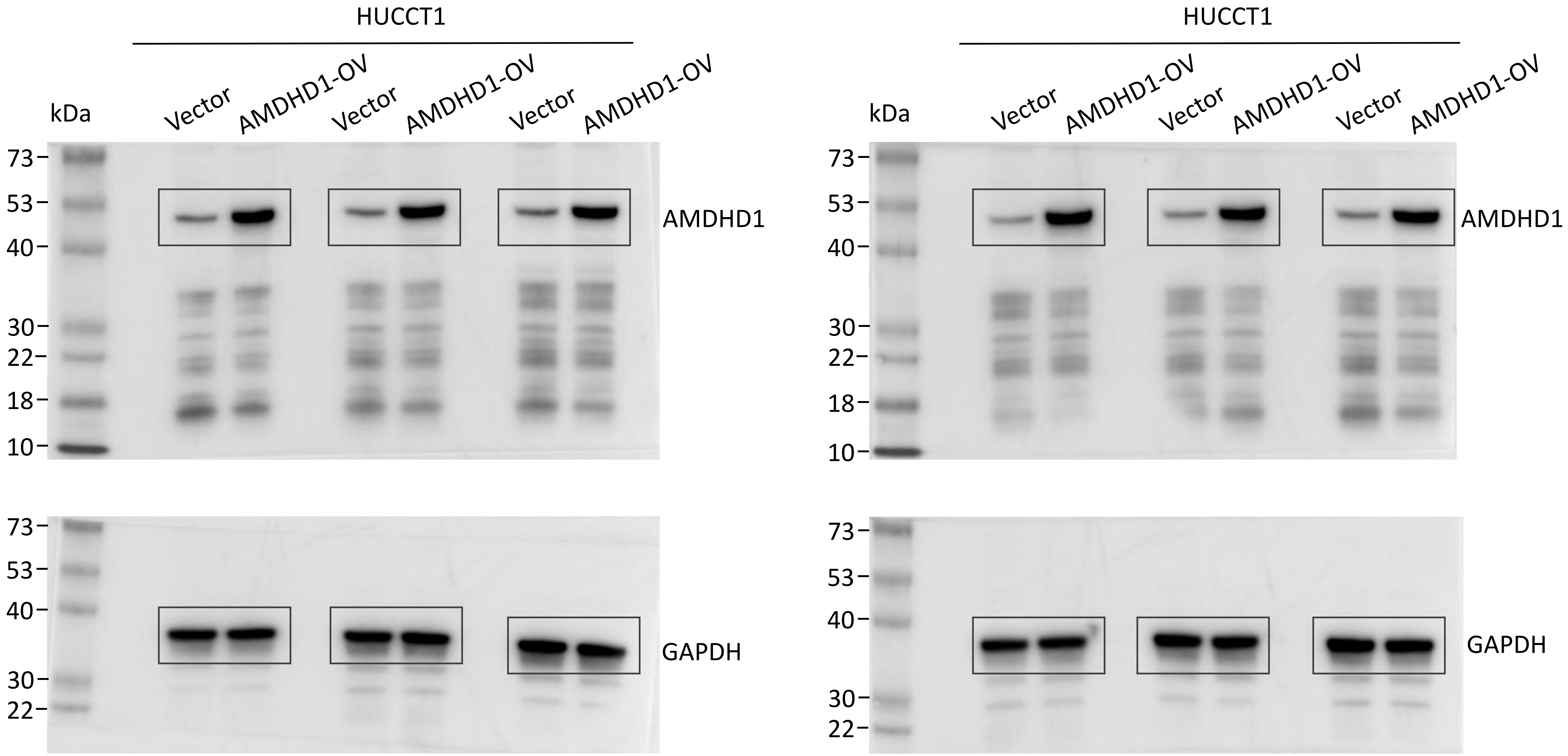
HUCCT1
kDa
Vector
AMDHD1-OV
Vector
AMDHD1-OV
Vector
AMDHD1-OV
73
53
40
30
22
18
10
AMDHD1
GAPDH
HUCCT1
kDa
Vector
AMDHD1-OV
Vector
AMDHD1-OV
Vector
AMDHD1-OV
73
53
40
30
22
18
10
AMDHD1
GAPDH

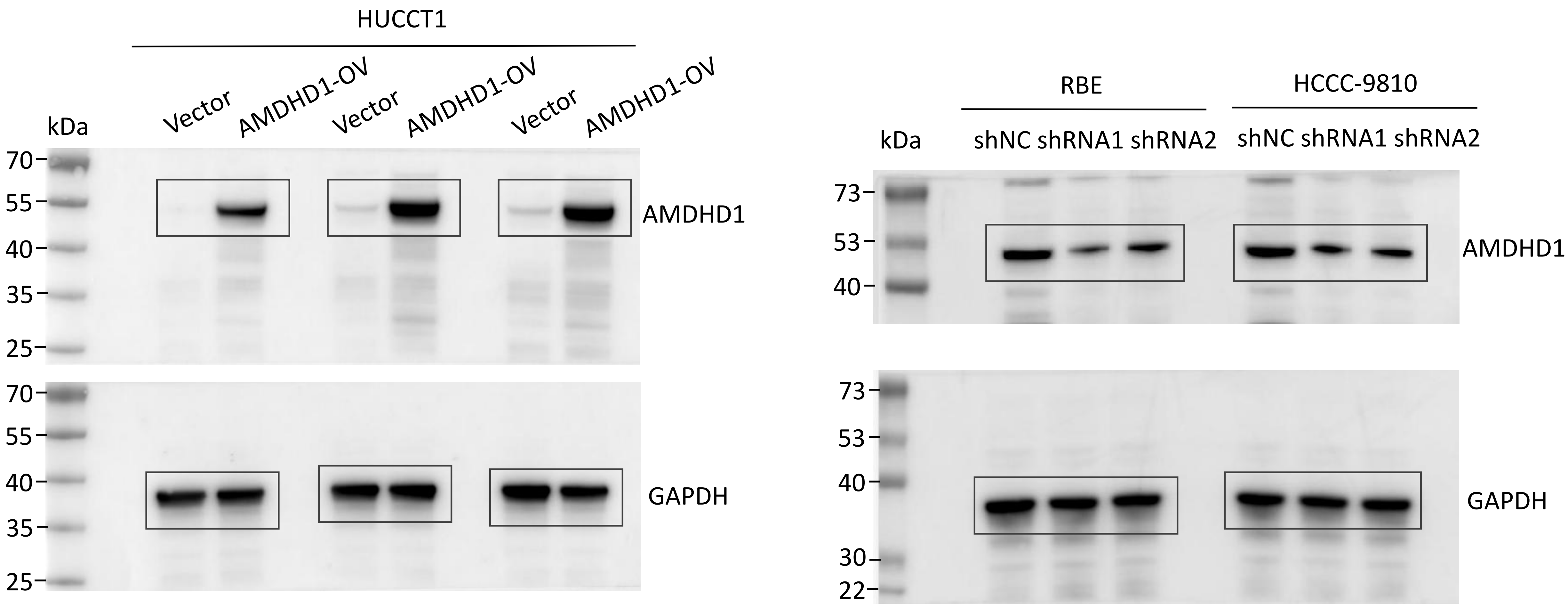
HUCCT1
kDa
Vector
AMDHD1-OV
Vector
AMDHD1-OV
Vector
AMDHD1-OV
70
55
40
35
25
AMDHD1
70
55
40
35
25
GAPDH
RBE
HCCC-9810
kDa
shNC
shRNA1
shRNA2
shNC
shRNA1
shRNA2
73
53
40
AMDHD1
73
53
40
30
22
GAPDH

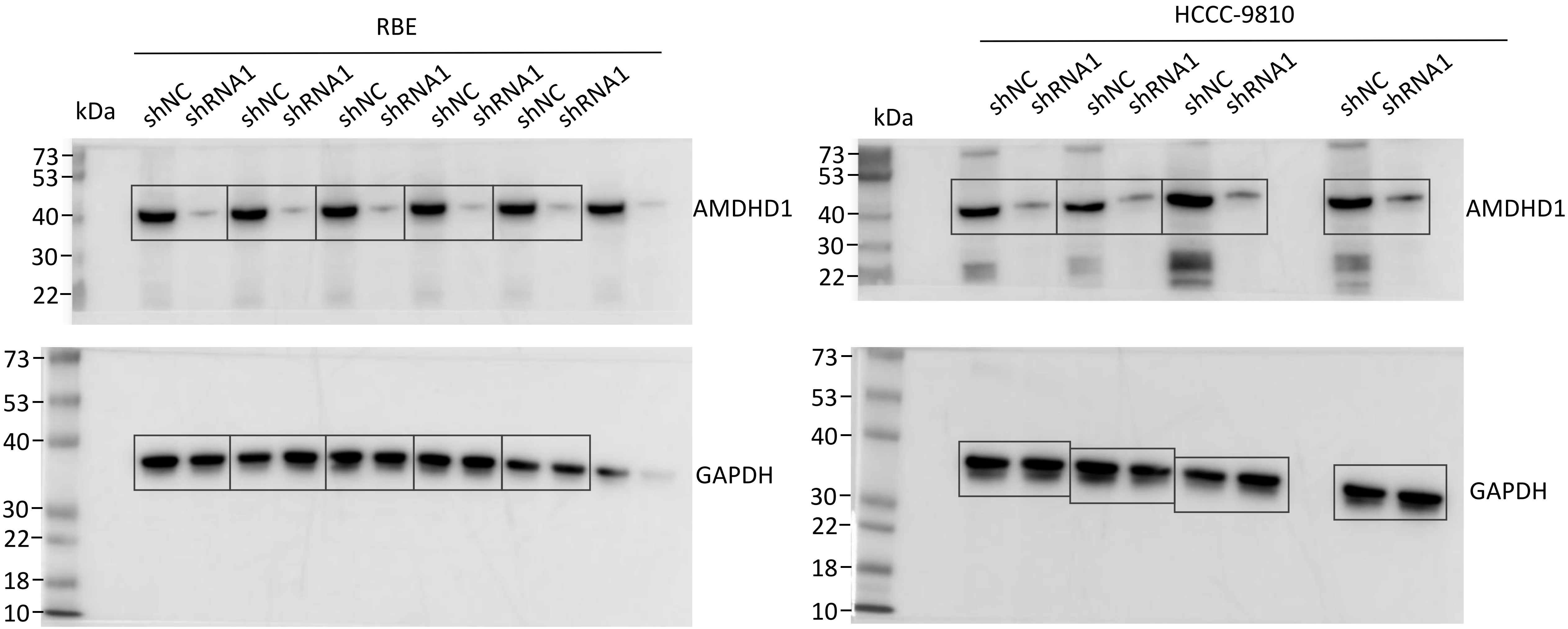

RBE
kDa
shNC
shRNA1
shNC
shRNA1
shNC
shRNA1
shNC
shRNA1
shNC
shRNA1
73
53
40
30
22
AMDHD1
73
53
40
30
22
18
10
GAPDH
HCCC-9810
kDa
shNC
shRNA1
shNC
shRNA1
shNC
shRNA1
shNC
shRNA1
73
53
40
30
22
AMDHD1
73
53
40
30
22
18
10
GAPDH

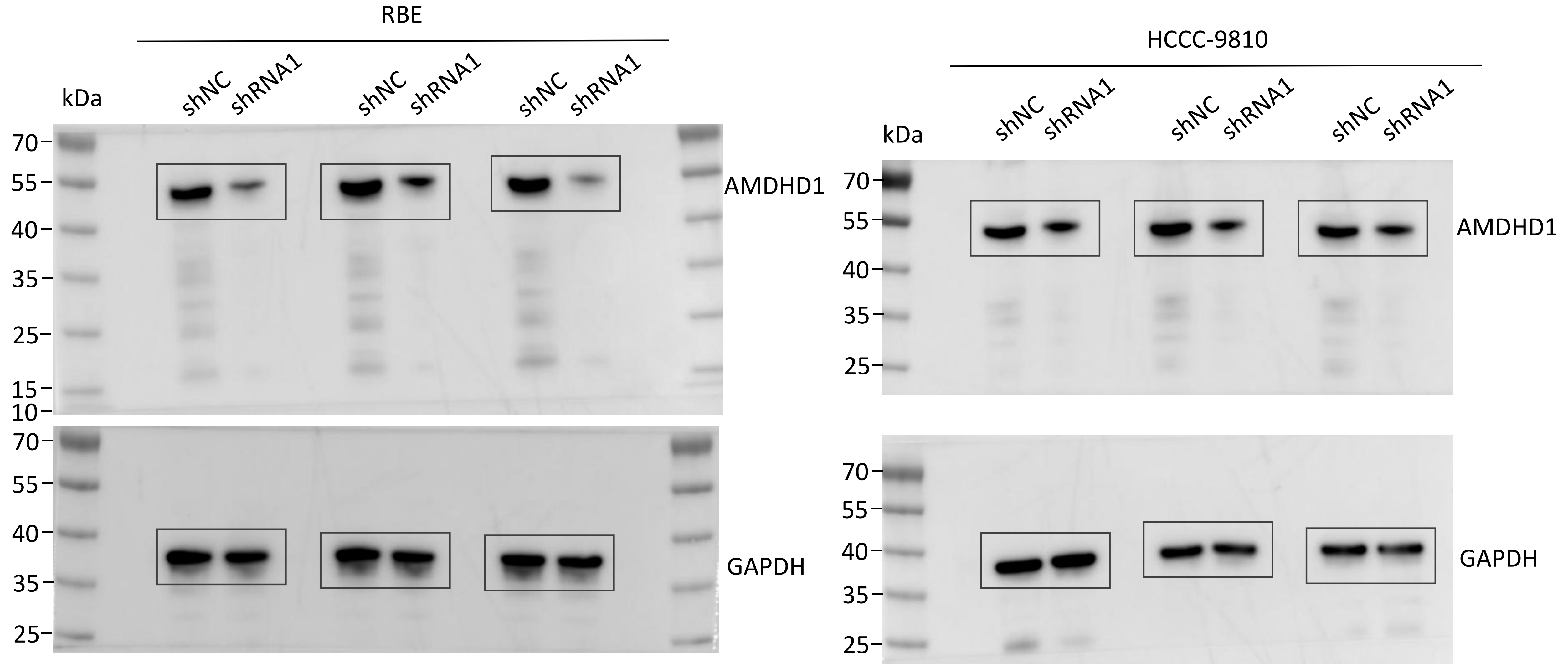
RBE
kDa
shNC
shRNA1
shNC
shRNA1
shNC
shRNA1
70
55
40
35
25
15
10
AMDHD1
70
55
40
35
25
GAPDH
HCCC-9810
kDa
shNC
shRNA1
shNC
shRNA1
shNC
shRNA1
70
55
40
35
25
AMDHD1
70
55
40
35
25
GAPDH

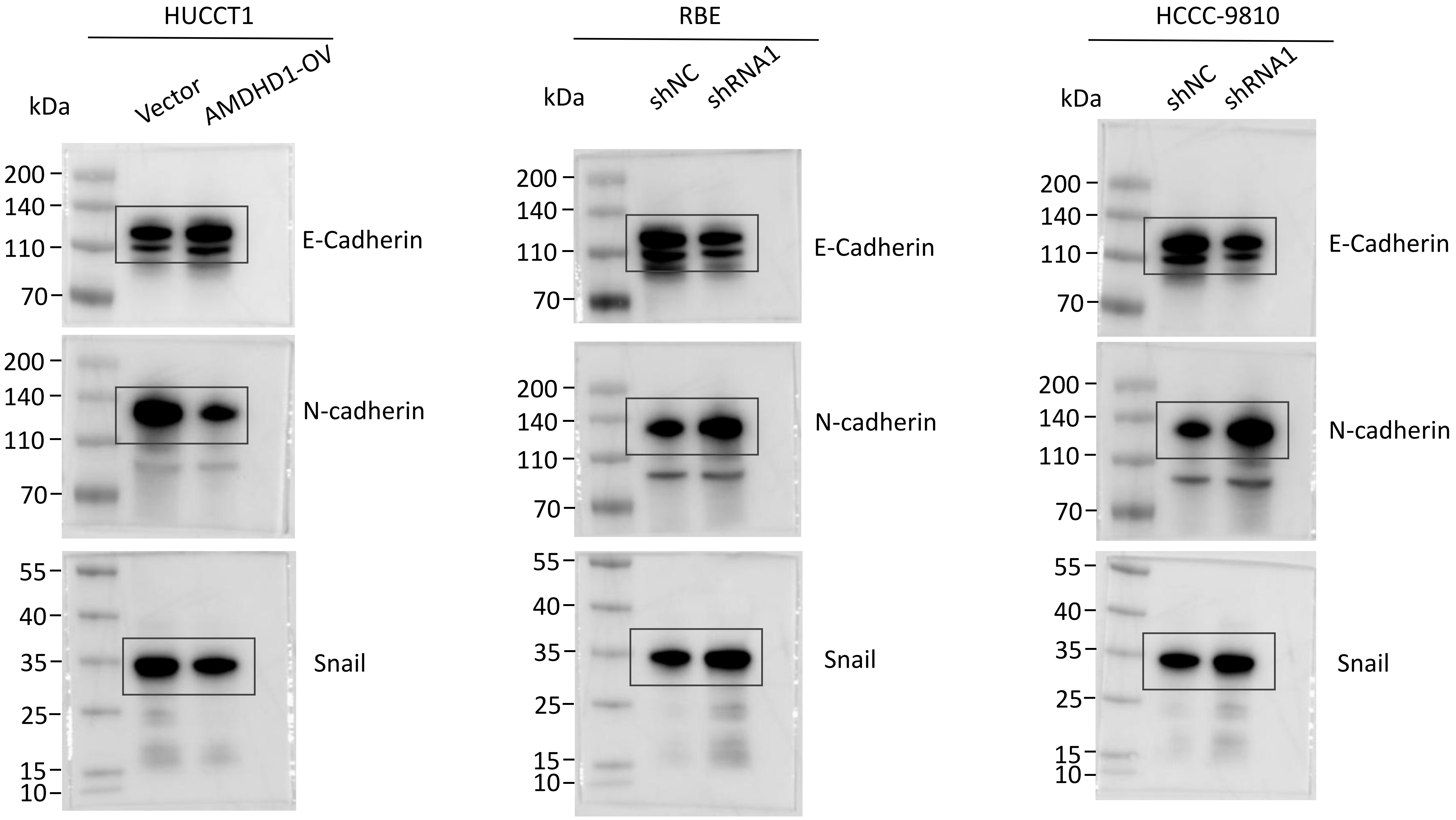
HUCCT1
kDa
Vector
AMDHD1-OV
200
140
110
70
E-Cadherin
N-cadherin
55
40
35
25
15
10
Snail
RBE
shNC
shRNA1
HCCC-9810

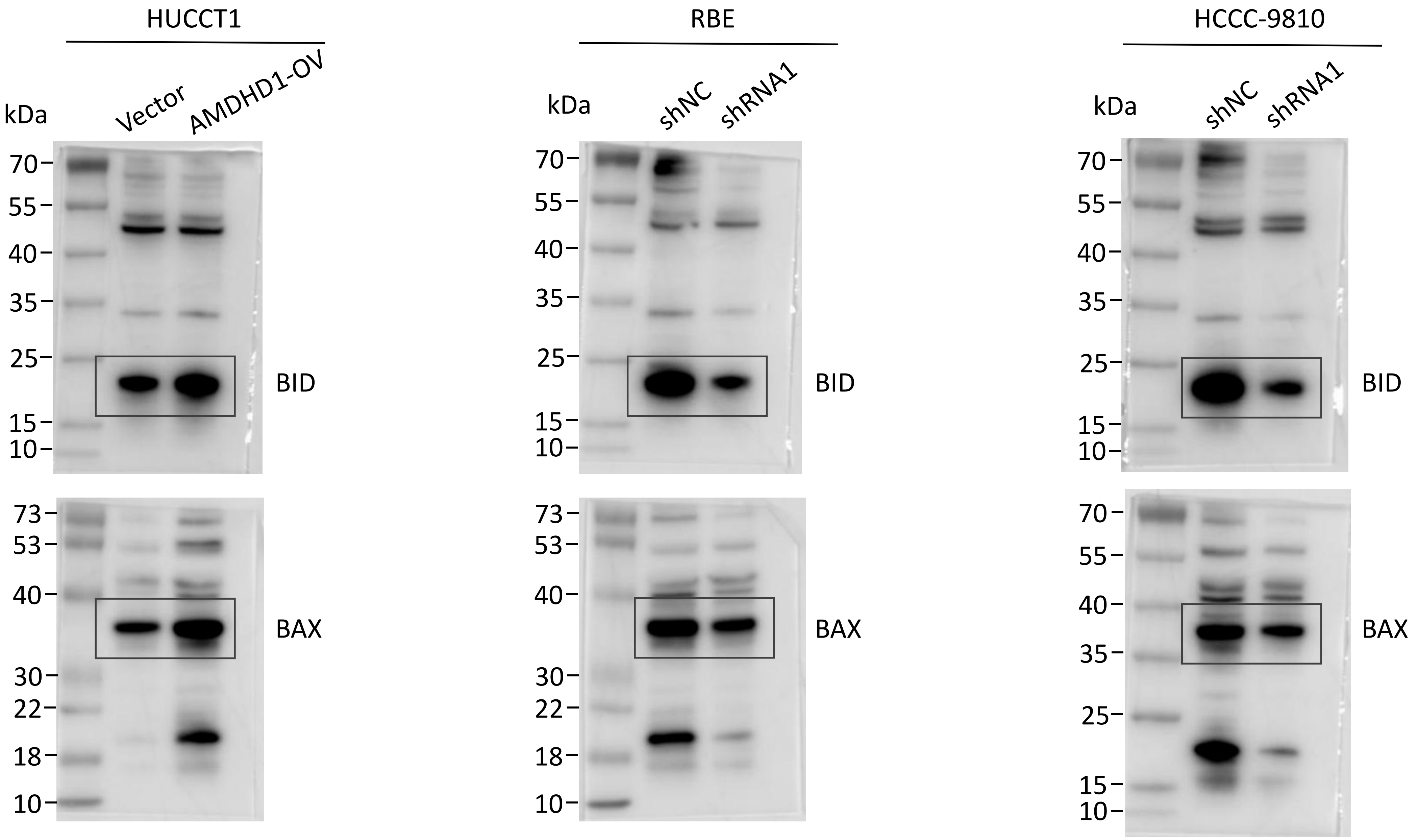

HUCCT1
kDa
Vector
AMDHD1-OV
70
55
40
35
25
15
10
BID
73
53
40
30
22
18
10
BAX
RBE
kDa
shNC
shRNA1
70
55
40
35
25
15
10
BID
73
53
40
30
22
18
10
BAX
HCCC-9810
kDa
shNC
shRNA1
70
55
40
35
25
15
10
BID
70
55
40
35
25
15
10
BAX

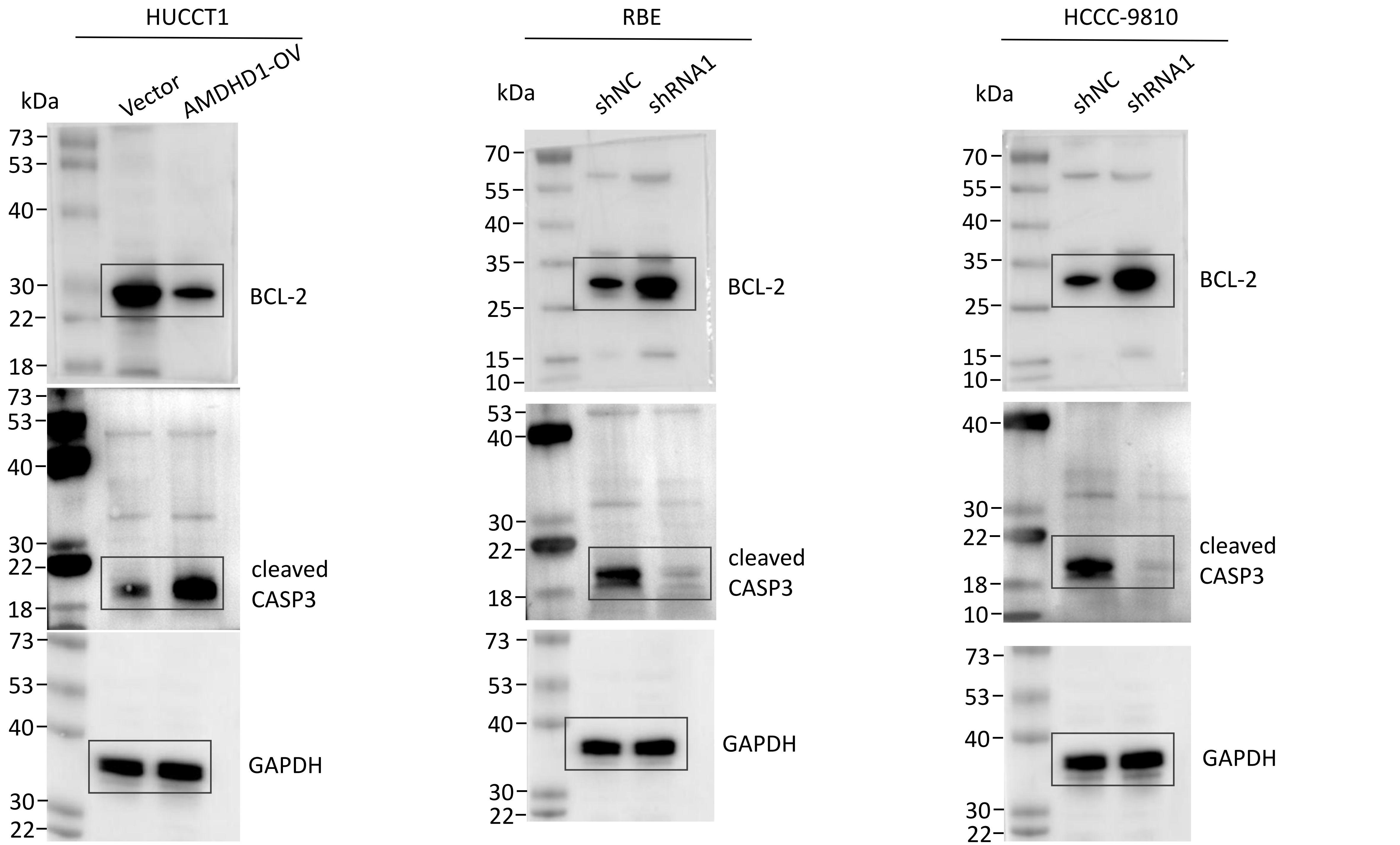
HUCCT1
kDa
Vector
AMDHD1-OV
73
53
40
30
22
18
BCL-2
73
53
40
30
22
18
cleaved
CASP3
73
53
40
30
22
GAPDH
RBE
kDa
shNC
shRNA1
70
55
40
35
25
15
10
BCL-2
53
40
30
22
18
cleaved
CASP3
73
53
40
30
22
GAPDH
HCCC-9810
kDa
shNC
shRNA1
70
55
40
35
25
15
10
BCL-2
40
30
22
18
10
cleaved
CASP3
73
53
40
30
22
GAPDH

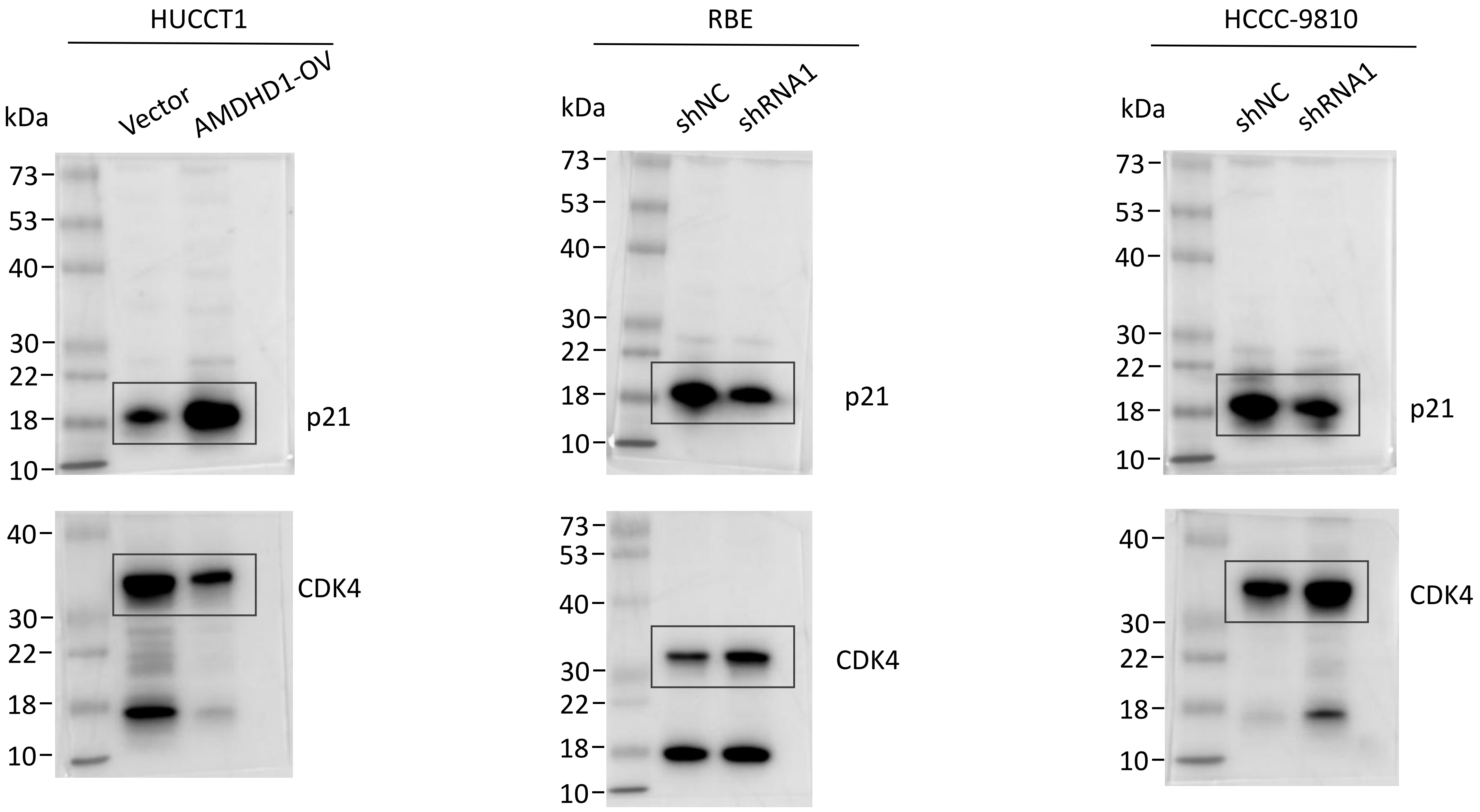

HUCCT1
RBE
HCCC-9810
kDa
Vector
AMDHD1-OV
shNC
shRNA1
73
53
40
30
22
18
10
p21
CDK4

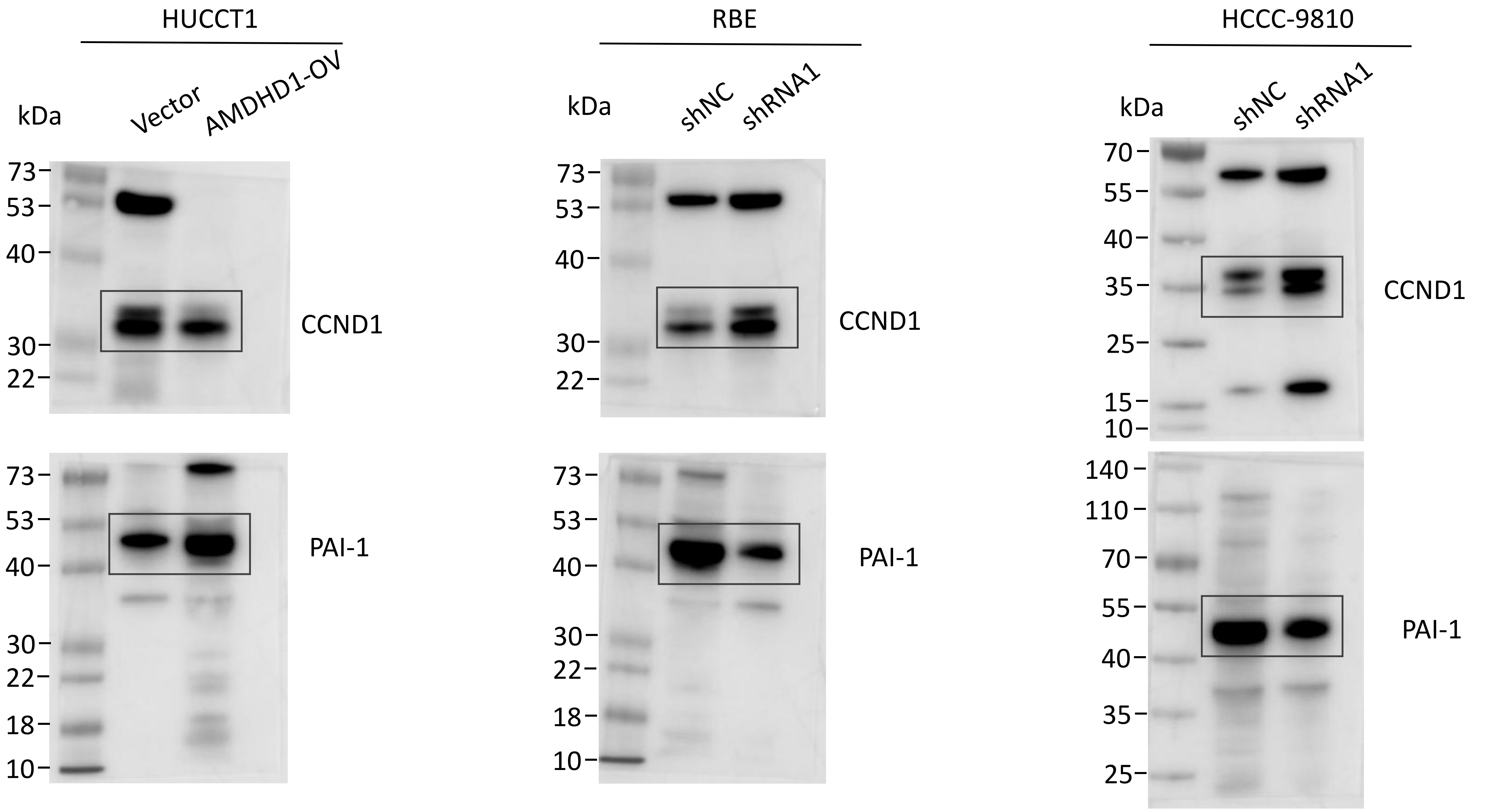

HUCCT1
kDa
Vector
AMDHD1-OV
73
53
40
30
22
CCND1
73
53
40
30
22
18
10
PAI-1
RBE
kDa
shNC
shRNA1
73
53
40
30
22
CCND1
73
53
40
30
22
18
10
PAI-1
HCCC-9810
kDa
shNC
shRNA1
70
55
40
35
25
15
10
CCND1
140
110
70
55
40
35
25
PAI-1

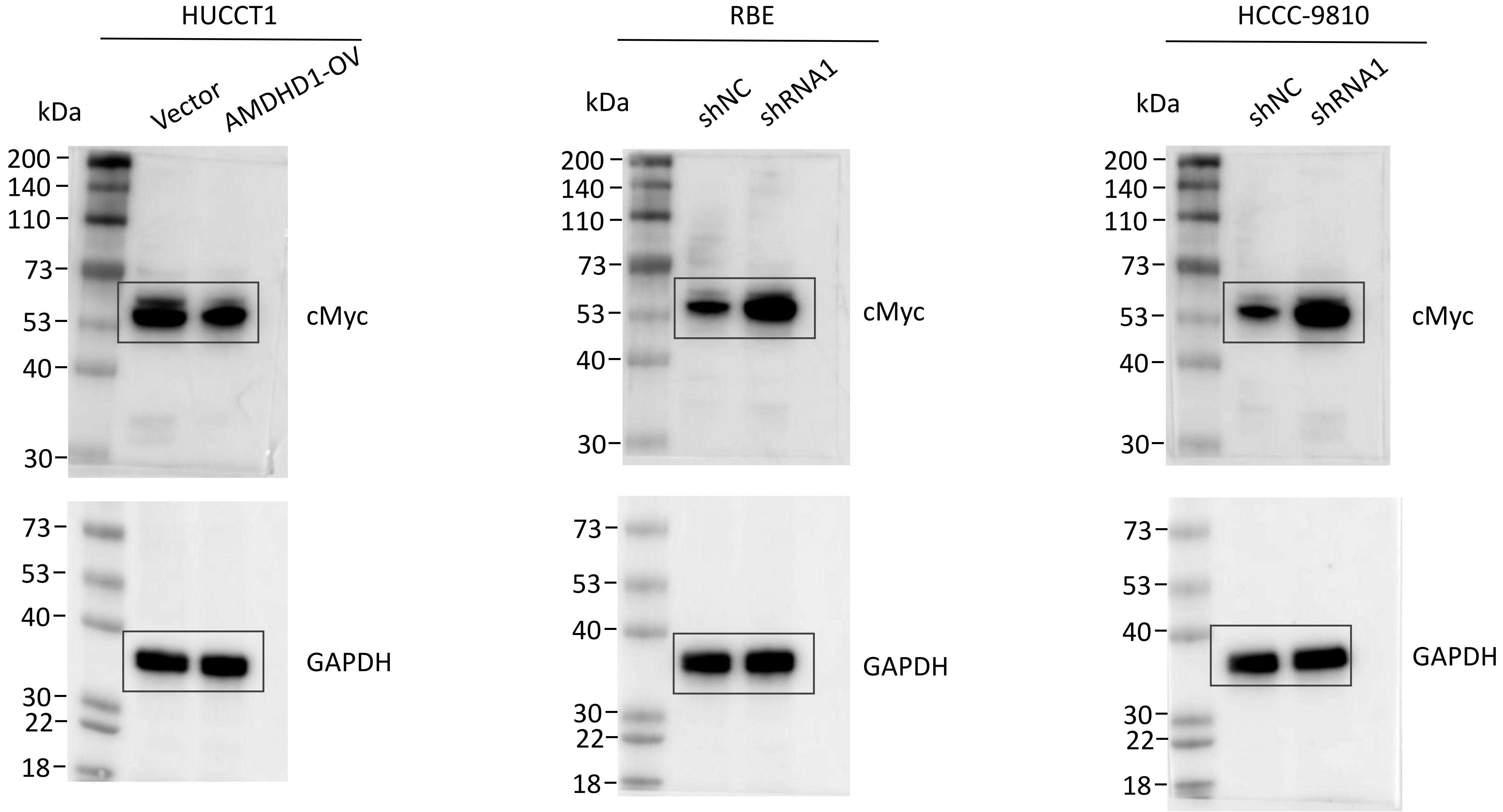

HUCCT1
kDa
Vector
AMDHD1-OV
200
140
110
73
53
40
30
cMyc
73
53
40
30
22
18
GAPDH
RBE
kDa
shNC
shRNA1
200
140
110
73
53
40
30
cMyc
73
53
40
30
22
18
GAPDH
HCCC-9810
kDa
shNC
shRNA1
200
140
110
73
53
40
30
cMyc
73
53
40
30
22
18
GAPDH

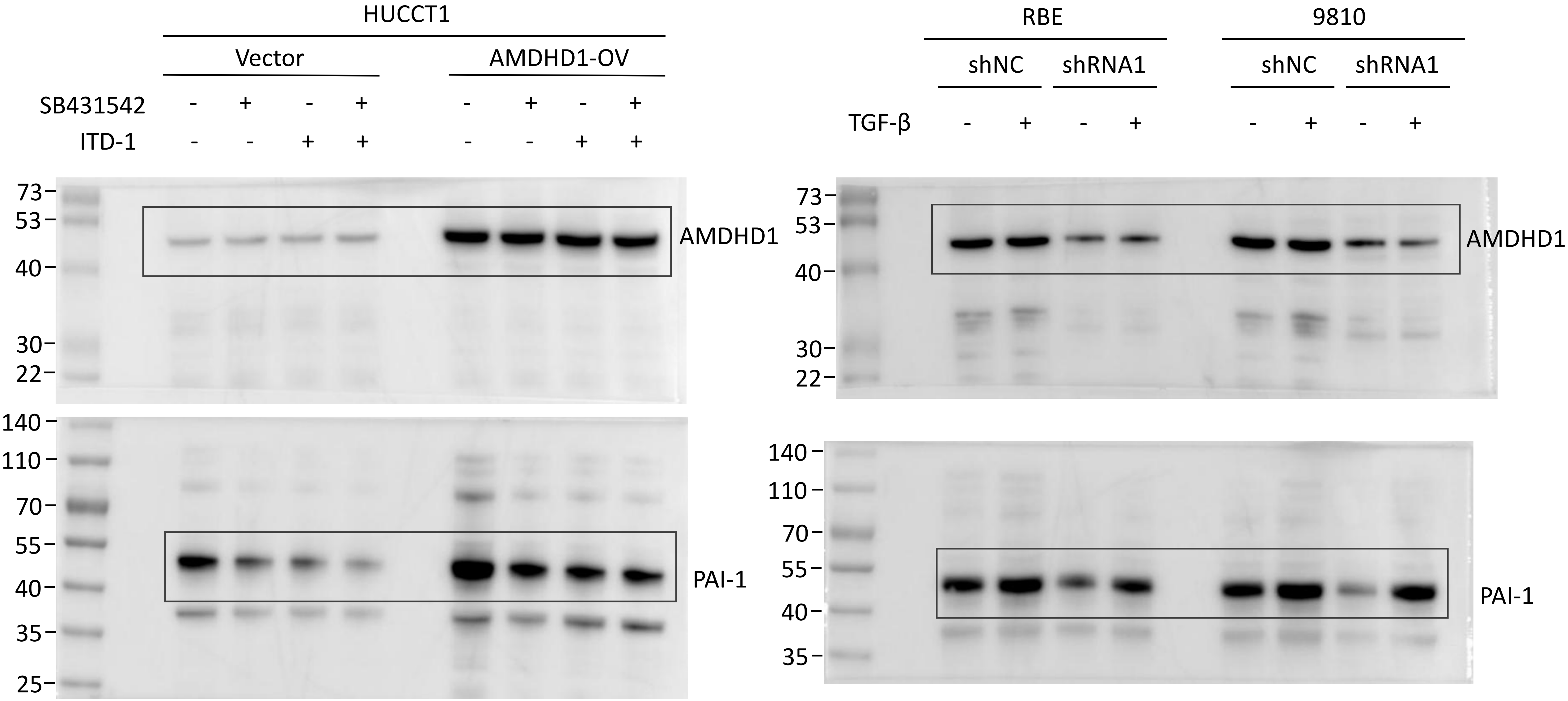
HUCCT1
Vector
AMDHD1-OV
SB431542 - + - + - + - +
ITD-1 - - + + - - + +
73
53
40
30
22
AMDHD1
140
110
70
55
40
35
25
PAI-1
RBE
9810
shNC
shRNA1
shNC
shRNA1
TGF-β - + - + - + - +
73
53
40
30
22
AMDHD1
140
110
70
55
40
35
PAI-1

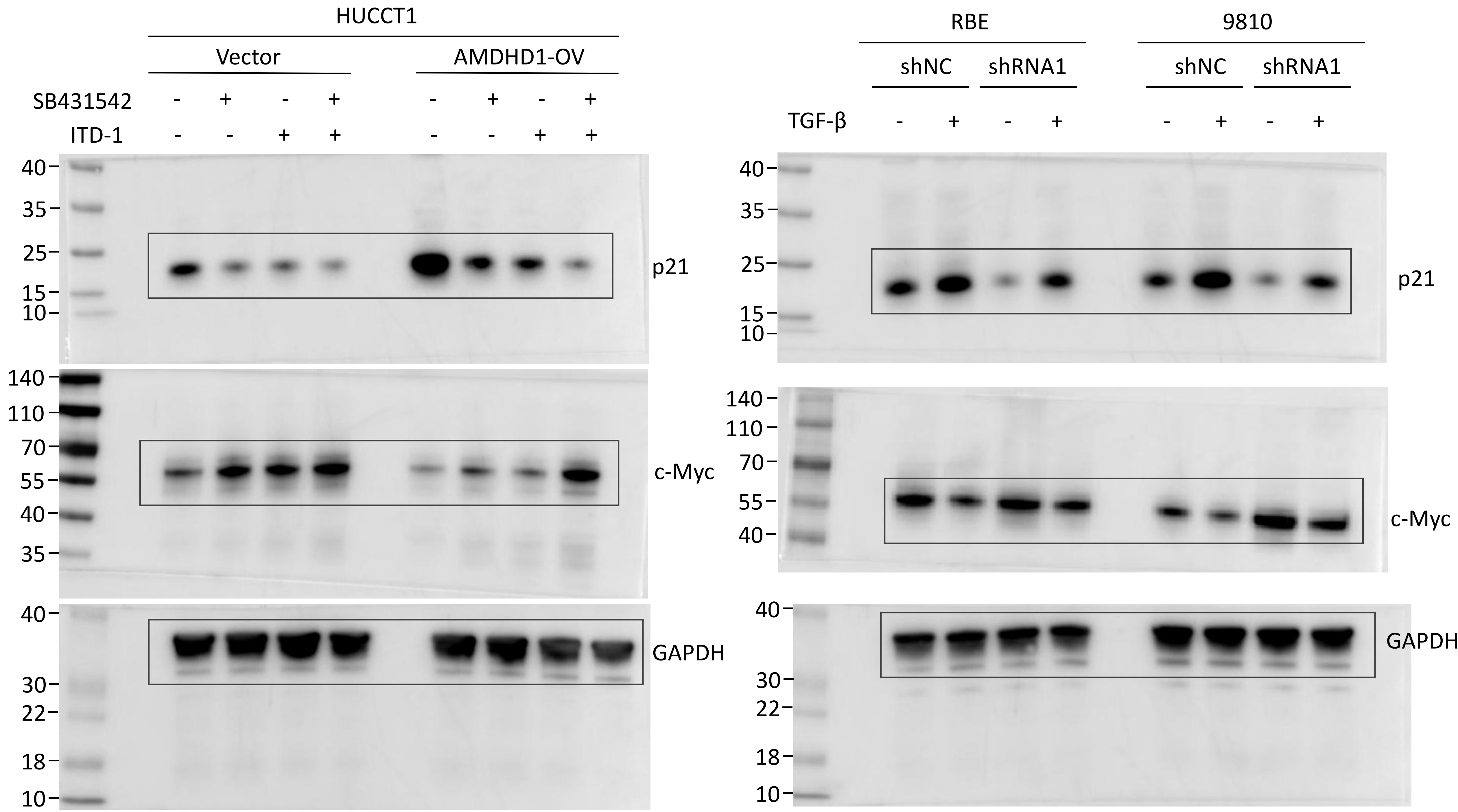
HUCCT1
Vector
AMDHD1-OV
SB431542
ITD-1
- + - + - + - +
- - + + - - + +
RBE
9810
shNC
shRNA1
shNC
shRNA1
TGF-β
- + - + - + - +
40
35
25
15
10
p21
140
110
70
55
40
35
c-Myc
40
30
22
18
10
GAPDH

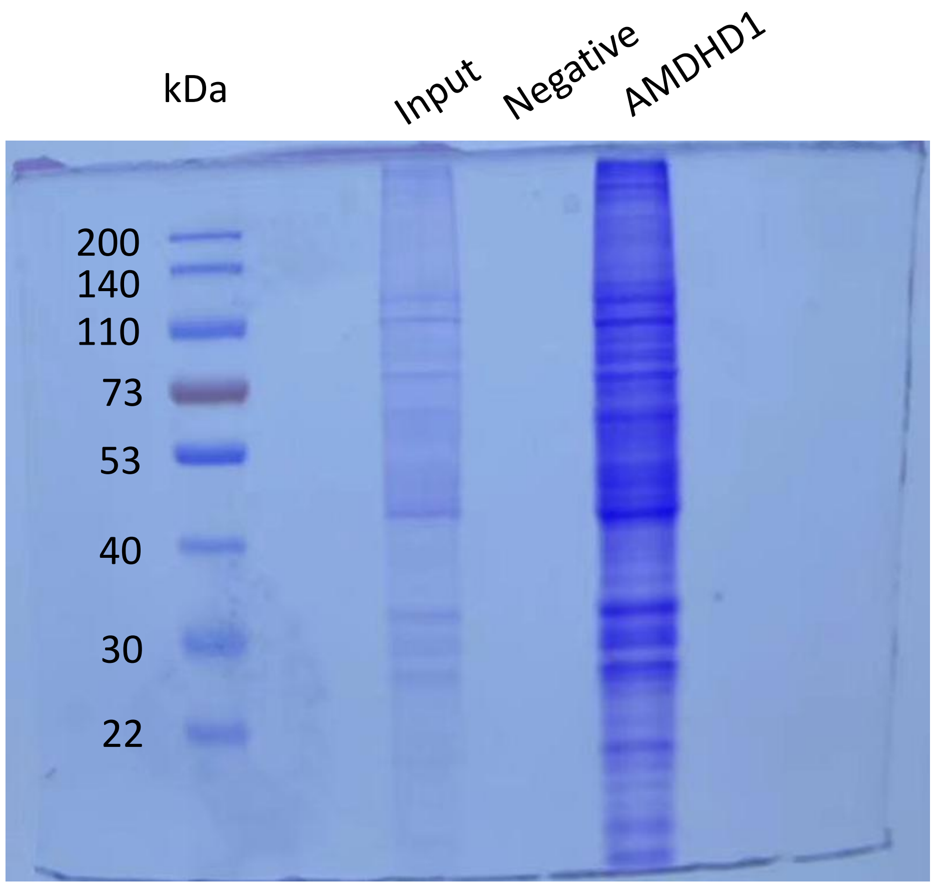
kDa
Input
Negative
AMDHD1
200
140
110
73
53
40
30
22

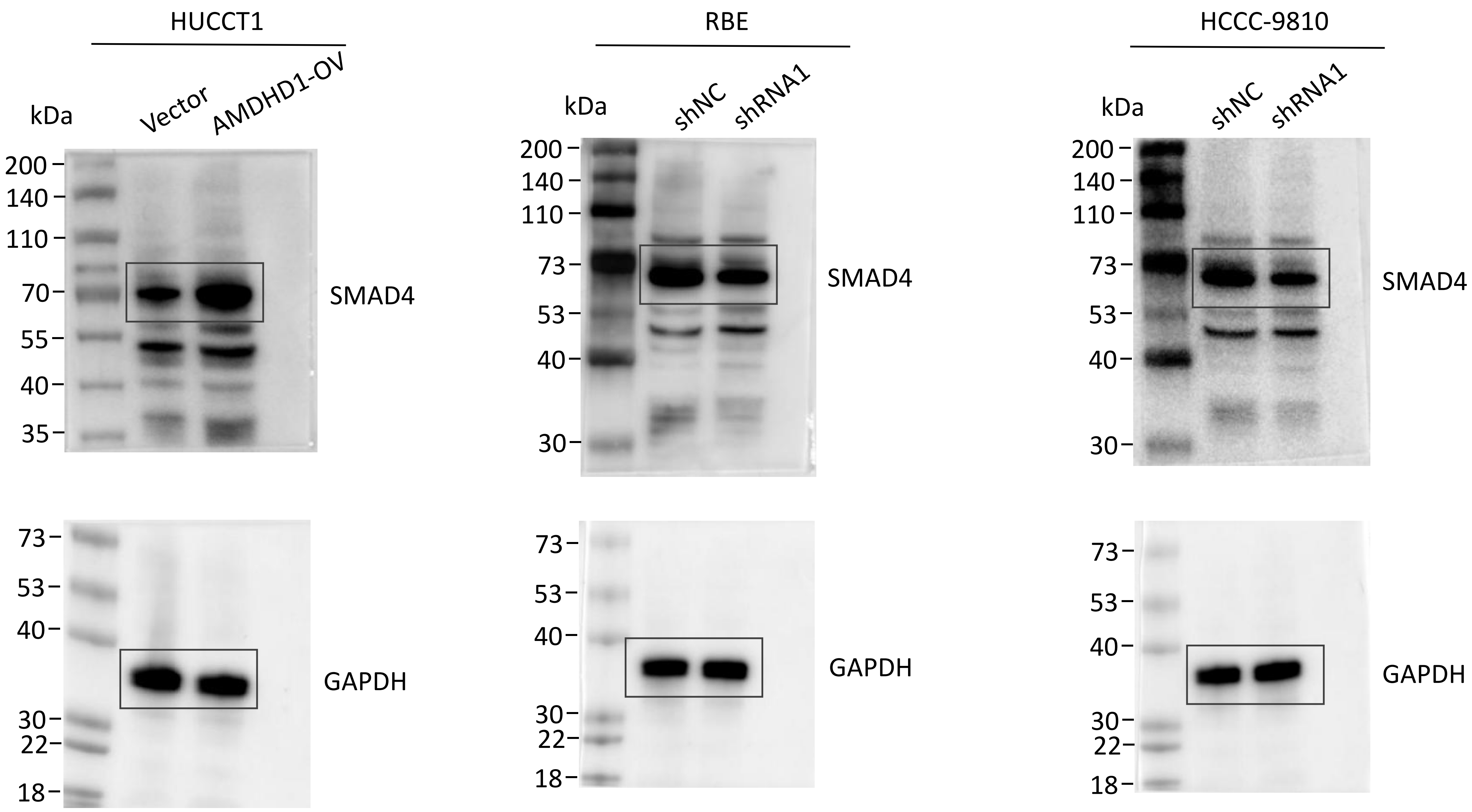
HUCCT1
kDa
Vector
AMDHD1-OV
200
140
110
70
55
40
35
SMAD4
73
53
40
30
22
18
GAPDH
RBE
kDa
shNC
shRNA1
200
140
110
73
53
40
30
SMAD4
73
53
40
30
22
18
GAPDH
HCCC-9810
kDa
shNC
shRNA1
200
140
110
73
53
40
30
SMAD4
73
53
40
30
22
18
GAPDH

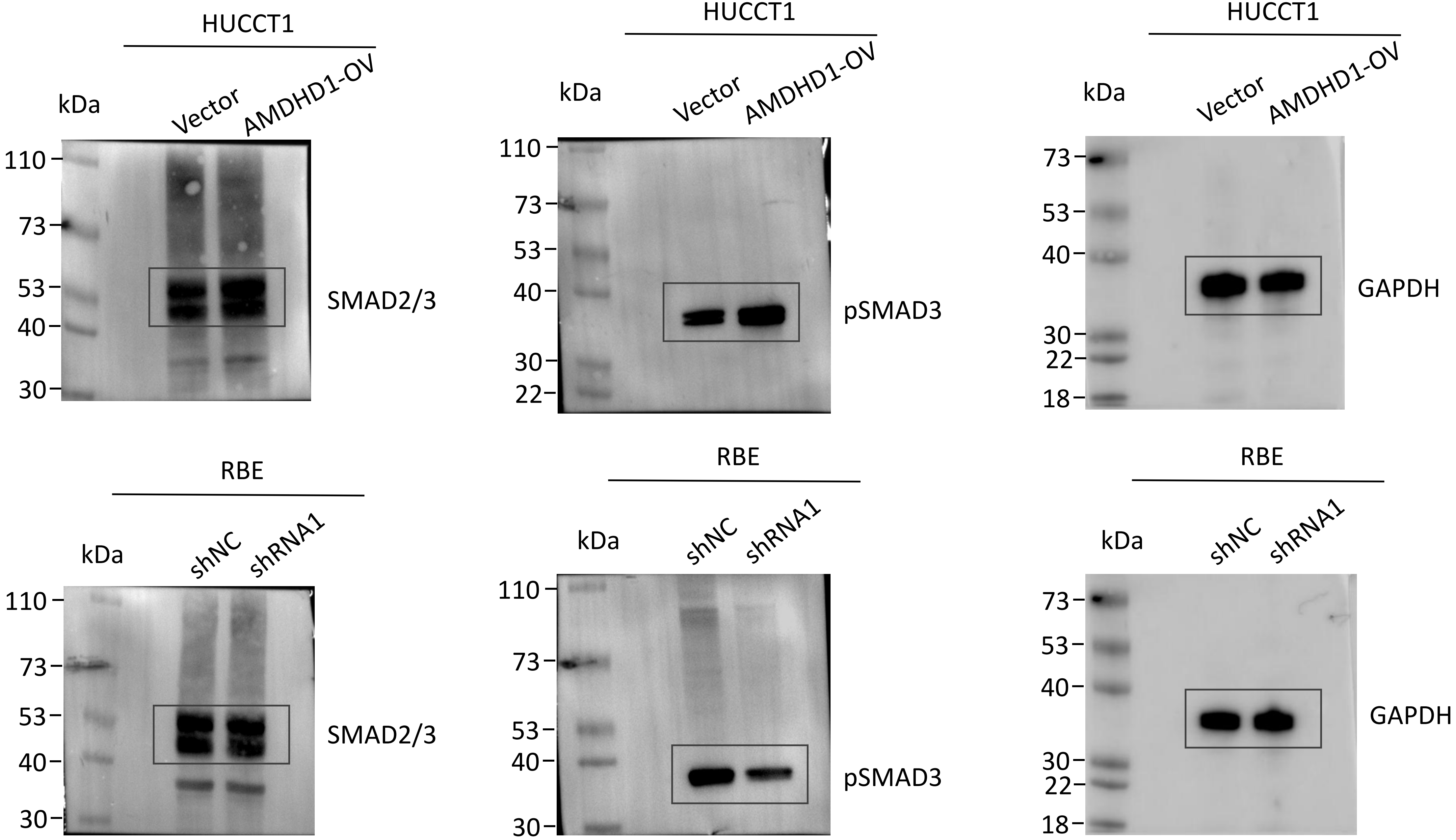
HUCCT1
kDa
Vector
AMDHD1-OV
110
73
53
40
30
SMAD2/3
HUCCT1
kDa
Vector
AMDHD1-OV
110
73
53
40
30
22
pSMAD3
HUCCT1
kDa
Vector
AMDHD1-OV
73
53
40
30
22
18
GAPDH
RBE
kDa
shNC
shRNA1
110
73
53
40
30
SMAD2/3
RBE
kDa
shNC
shRNA1
110
73
53
40
30
pSMAD3
RBE
kDa
shNC
shRNA1
73
53
40
30
22
18
GAPDH

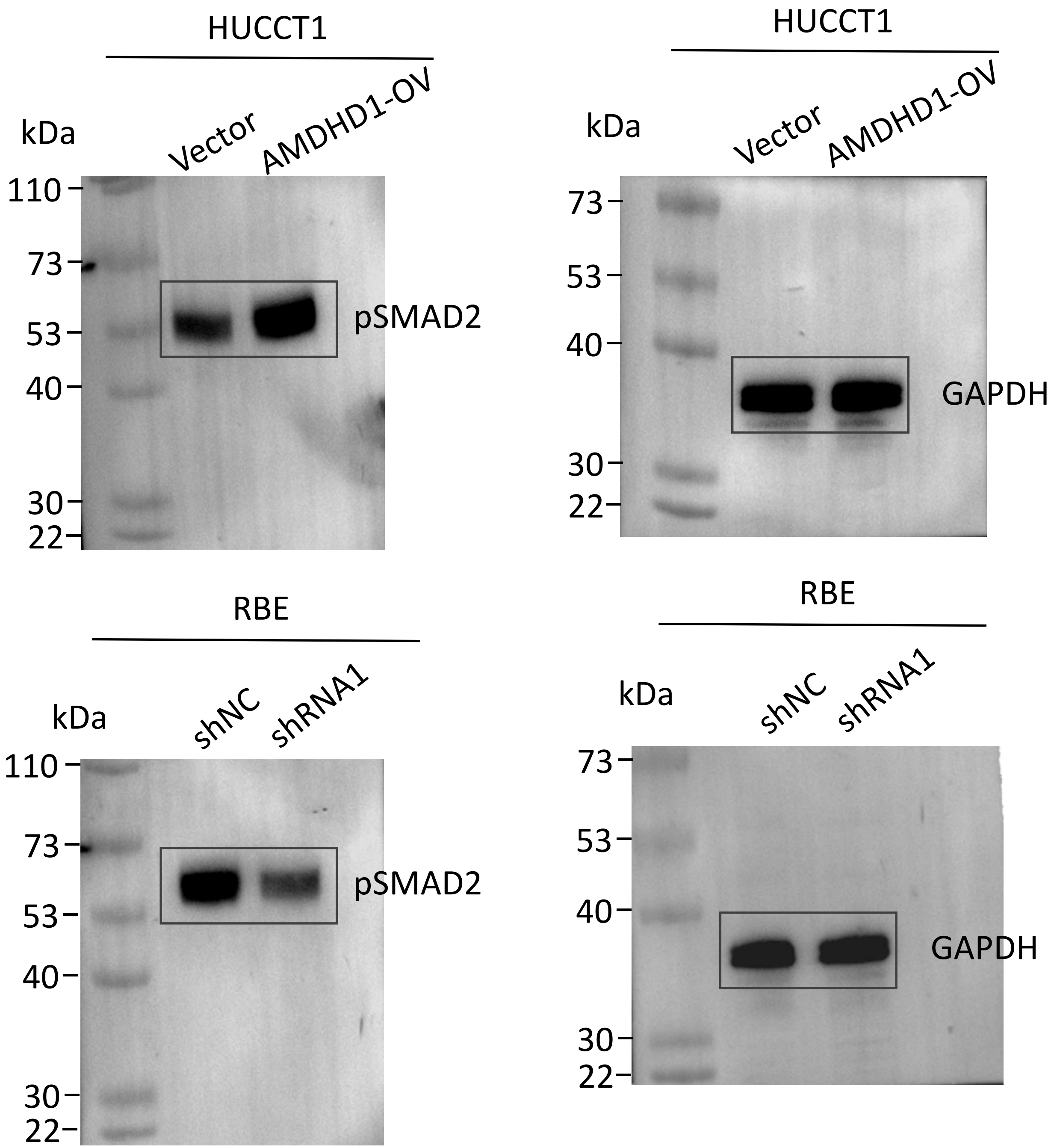
HUCCT1
kDa
Vector
AMDHD1-OV
110
73
53
40
30
22
pSMAD2
HUCCT1
kDa
Vector
AMDHD1-OV
73
53
40
30
22
GAPDH
RBE
kDa
shNC
shRNA1
110
73
53
40
30
22
pSMAD2
RBE
kDa
shNC
shRNA1
73
53
40
30
22
GAPDH

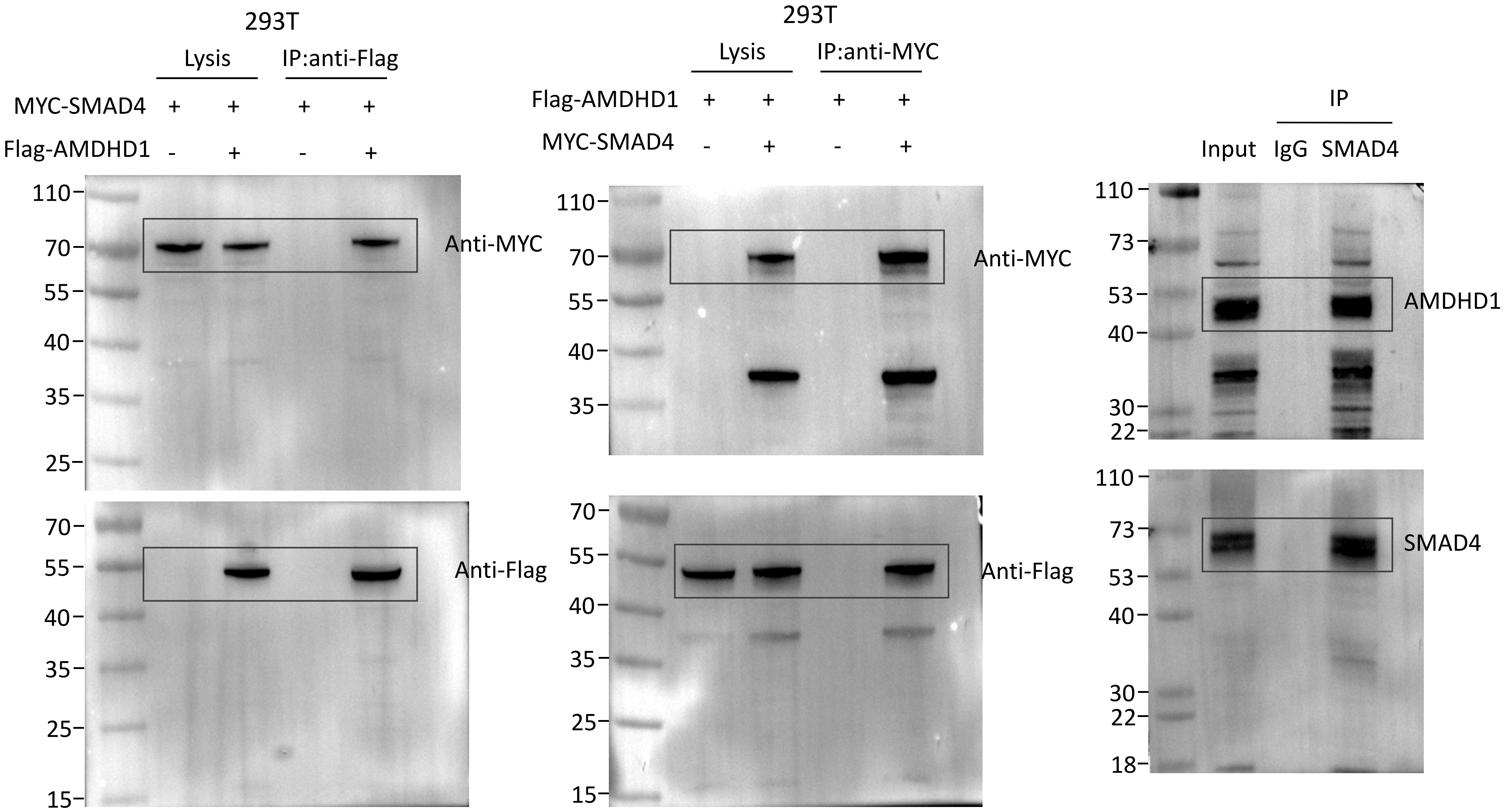
293T
Lysis
IP:anti-Flag
MYC-SMAD4 + + + +
Flag-AMDHD1 - + - +
110
70
55
40
35
25
Anti-MYC
70
55
40
35
25
15
Anti-Flag
293T
Lysis
IP:anti-MYC
Flag-AMDHD1 + + + +
MYC-SMAD4 - + - +
110
70
55
40
35
Anti-MYC
70
55
40
35
25
15
Anti-Flag
IP
Input
IgG
SMAD4
110
73
53
40
30
22
AMDHD1
110
73
53
40
30
22
18
SMAD4

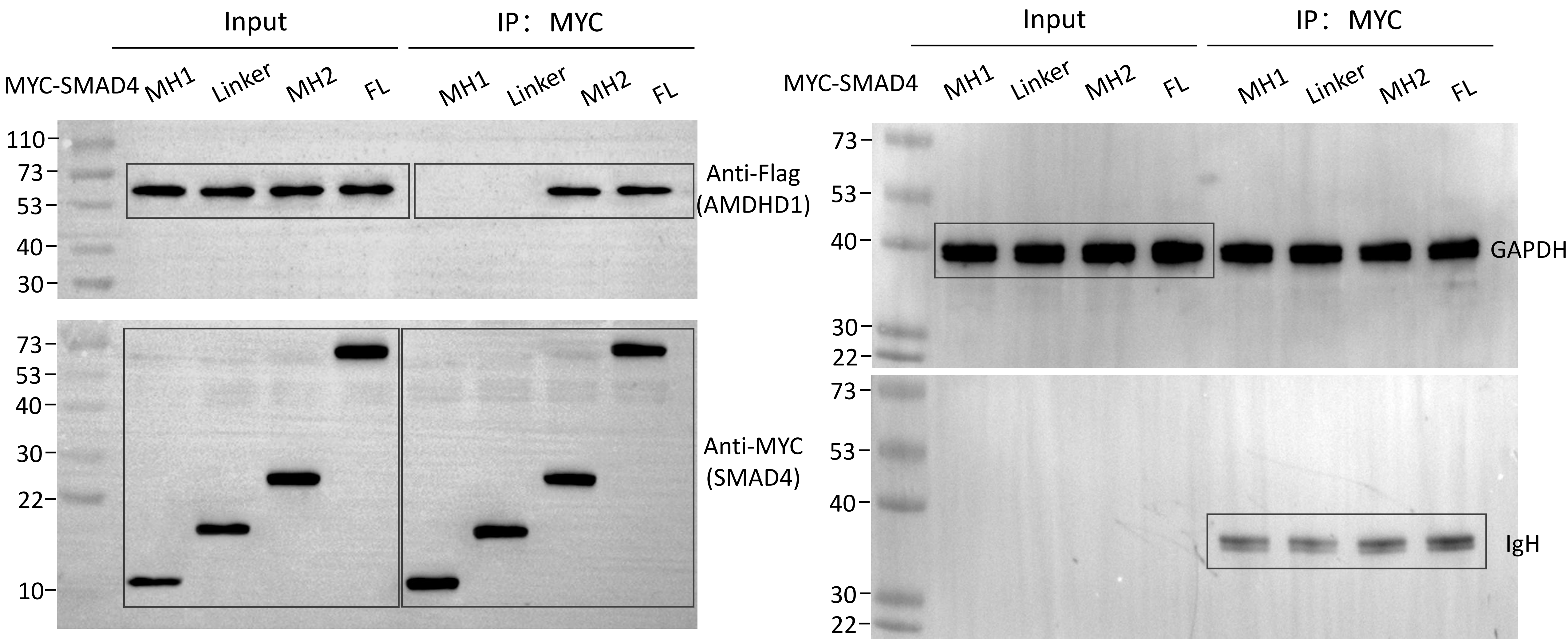

Input
IP： MYC
MYC-SMAD4
MH1
Linker
MH2
FL
110
73
53
40
30
22
10
Anti-Flag
(AMDHD1)
Anti-MYC
(SMAD4)
GAPDH
IgH

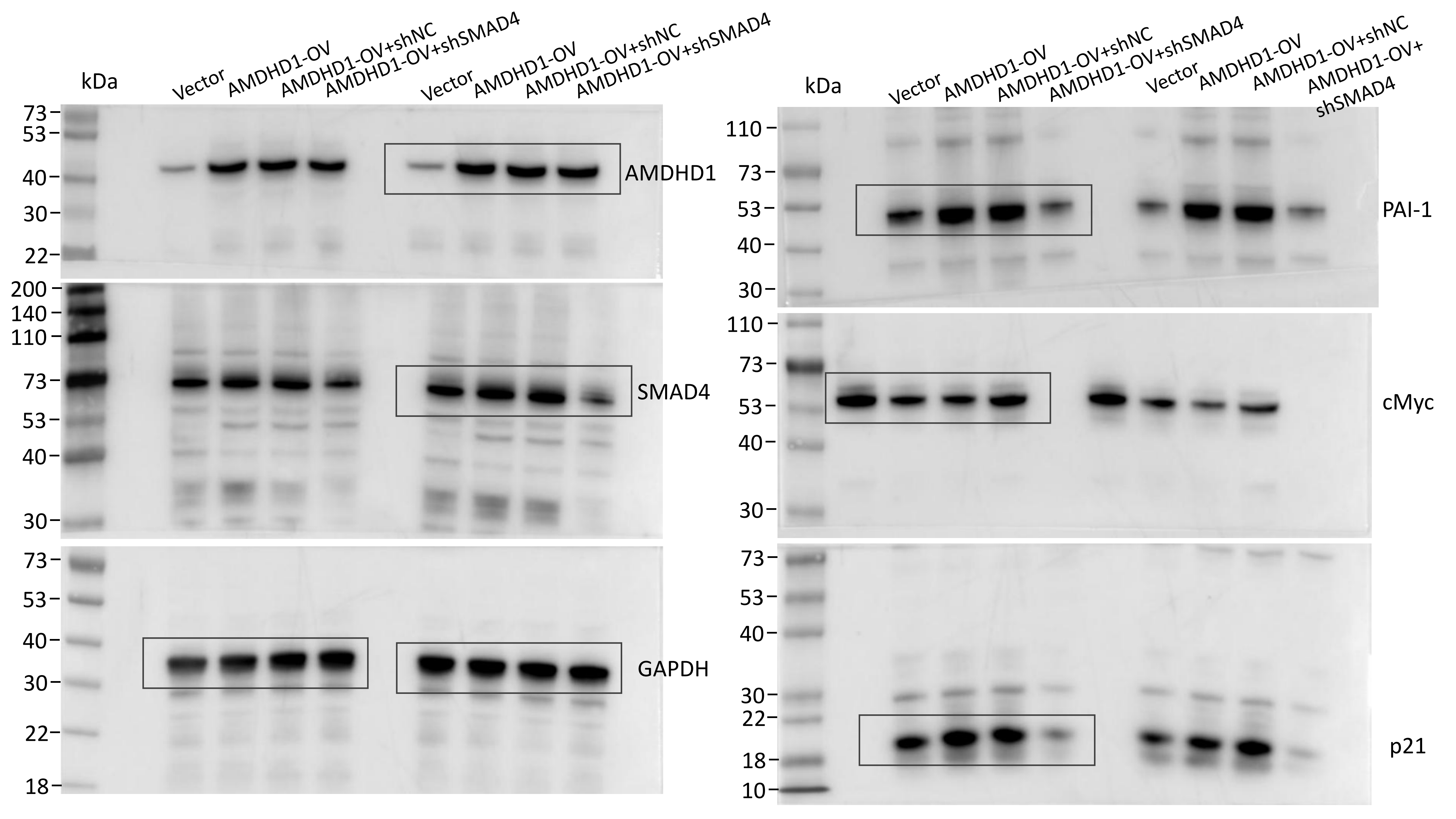
kDa
Vector
AMDHD1-OV
AMDHD1-OV+shNC
AMDHD1-OV+shSMAD4
Vector
AMDHD1-OV
AMDHD1-OV+shNC
AMDHD1-OV+shSMAD4
73
53
40
30
22
AMDHD1
200
140
110
73
53
40
30
SMAD4
73
53
40
30
22
18
GAPDH
kDa
Vector
AMDHD1-OV
AMDHD1-OV+shNC
AMDHD1-OV+shSMAD4
Vector
AMDHD1-OV
AMDHD1-OV+shNC
AMDHD1-OV+
shSMAD4
110
73
53
40
30
PAI-1
110
73
53
40
30
cMyc
73
53
40
30
22
18
10
p21

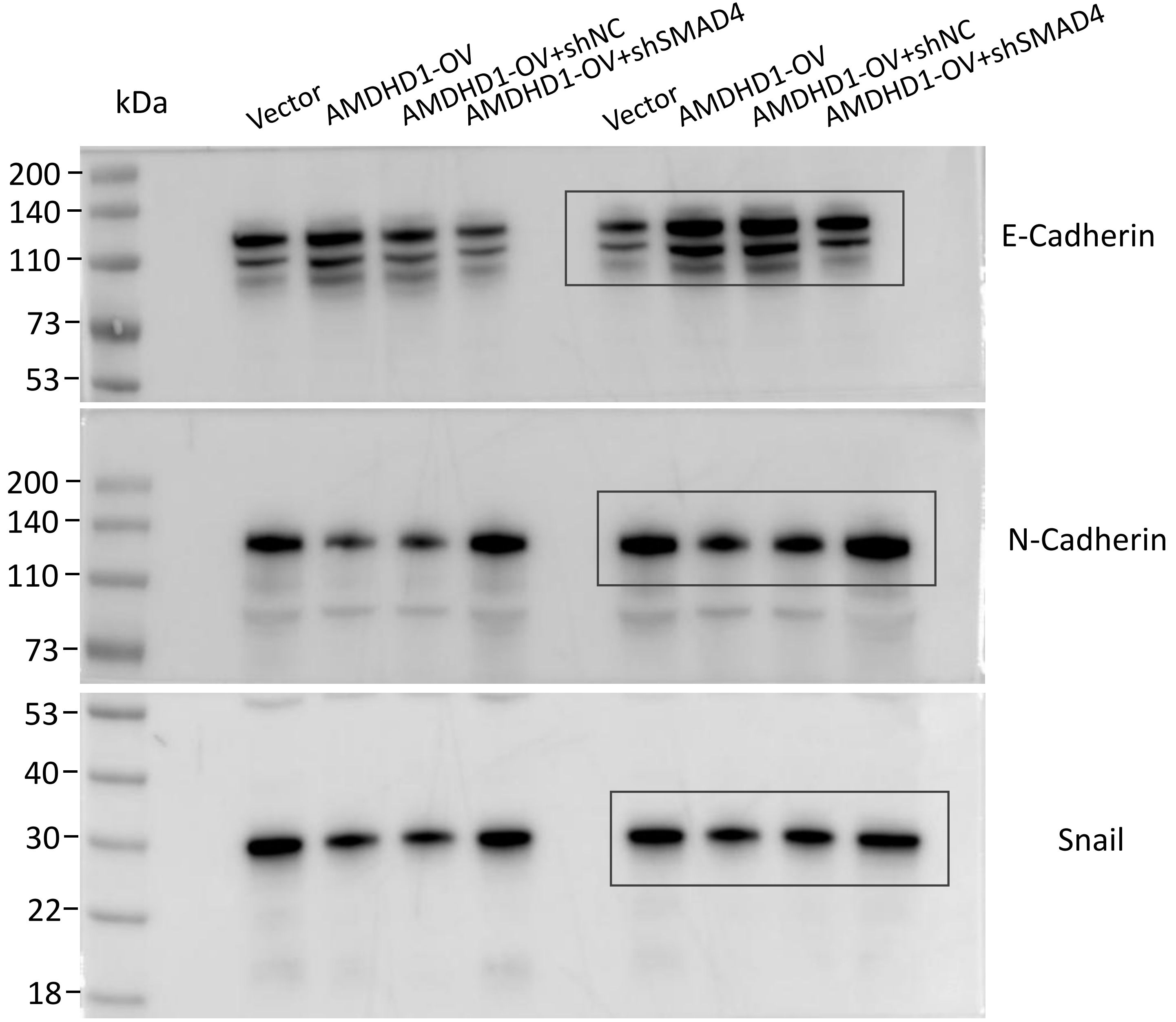

kDa
Vector
AMDHD1-OV
AMDHD1-OV+shNC
AMDHD1-OV+shSMAD4
Vector
AMDHD1-OV
AMDHD1-OV+shNC
AMDHD1-OV+shSMAD4
200
140
110
73
53
E-Cadherin
200
140
110
73
N-Cadherin
53
40
30
22
18
Snail

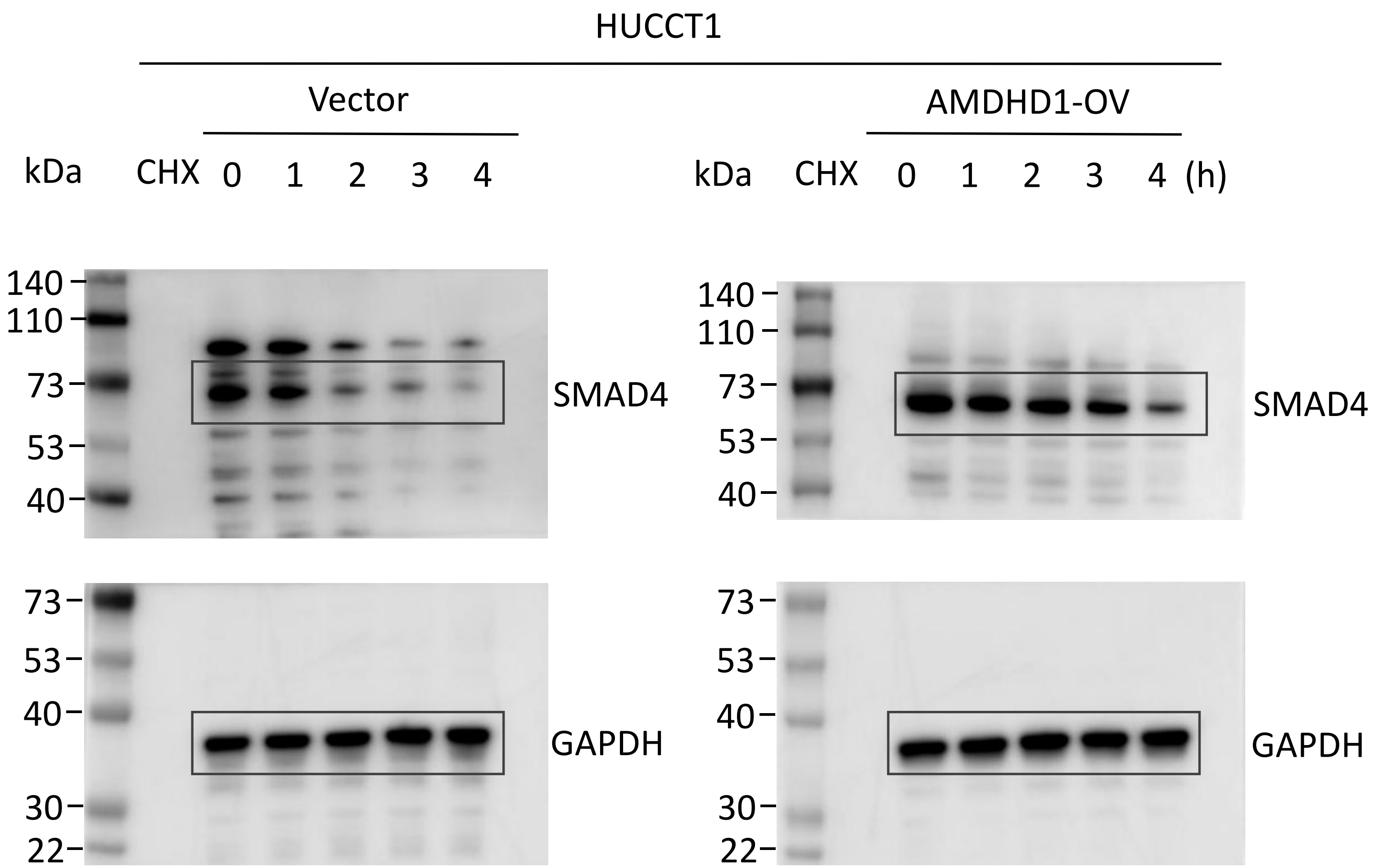
HUCCT1
Vector
AMDHD1-OV
kDa
CHX
0
1
2
3
4
(h)
140
110
73
53
40
30
22
SMAD4
GAPDH

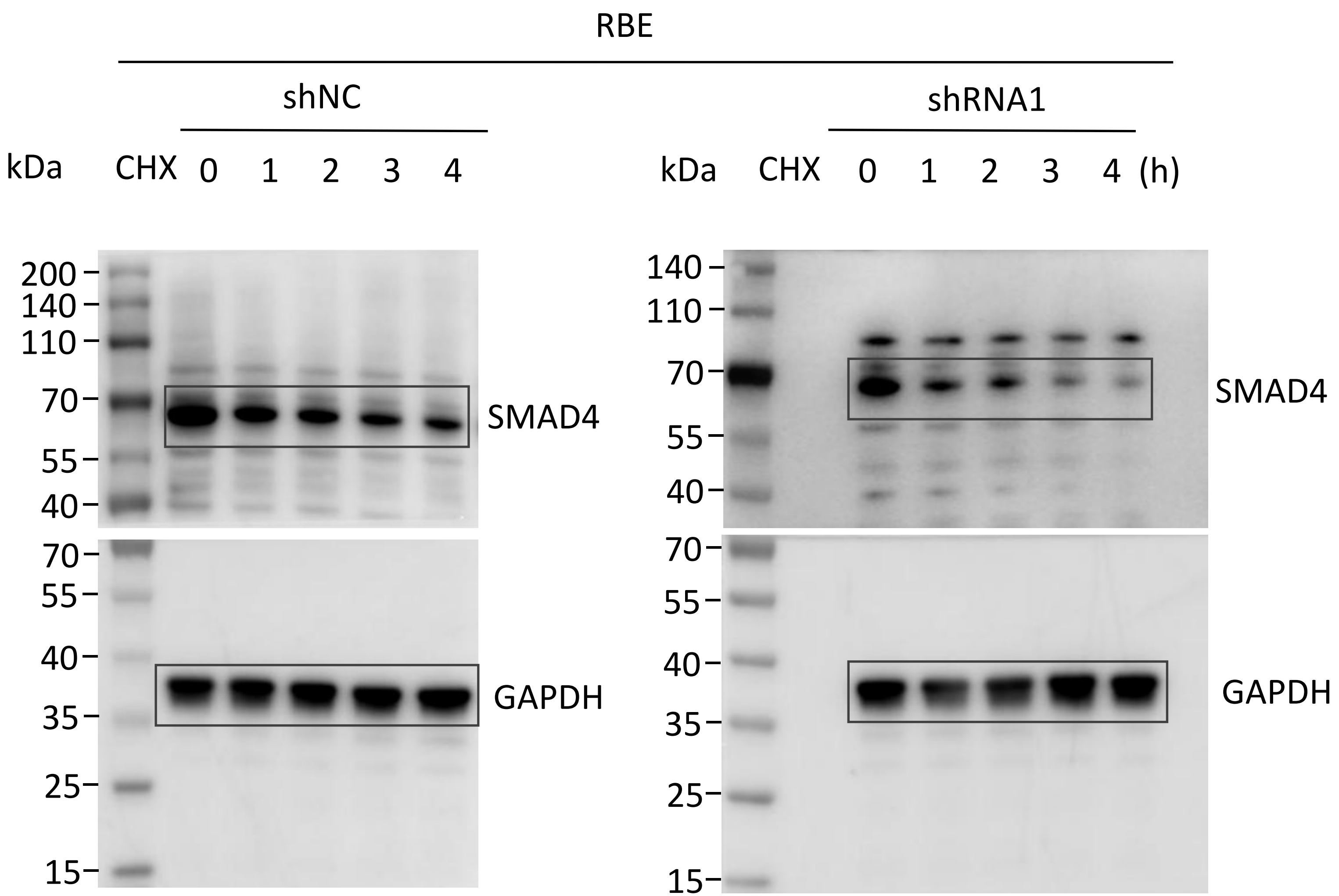
RBE
shNC
shRNA1
kDa
CHX
0
1
2
3
4
(h)
200
140
110
70
55
40
35
25
15
SMAD4
GAPDH

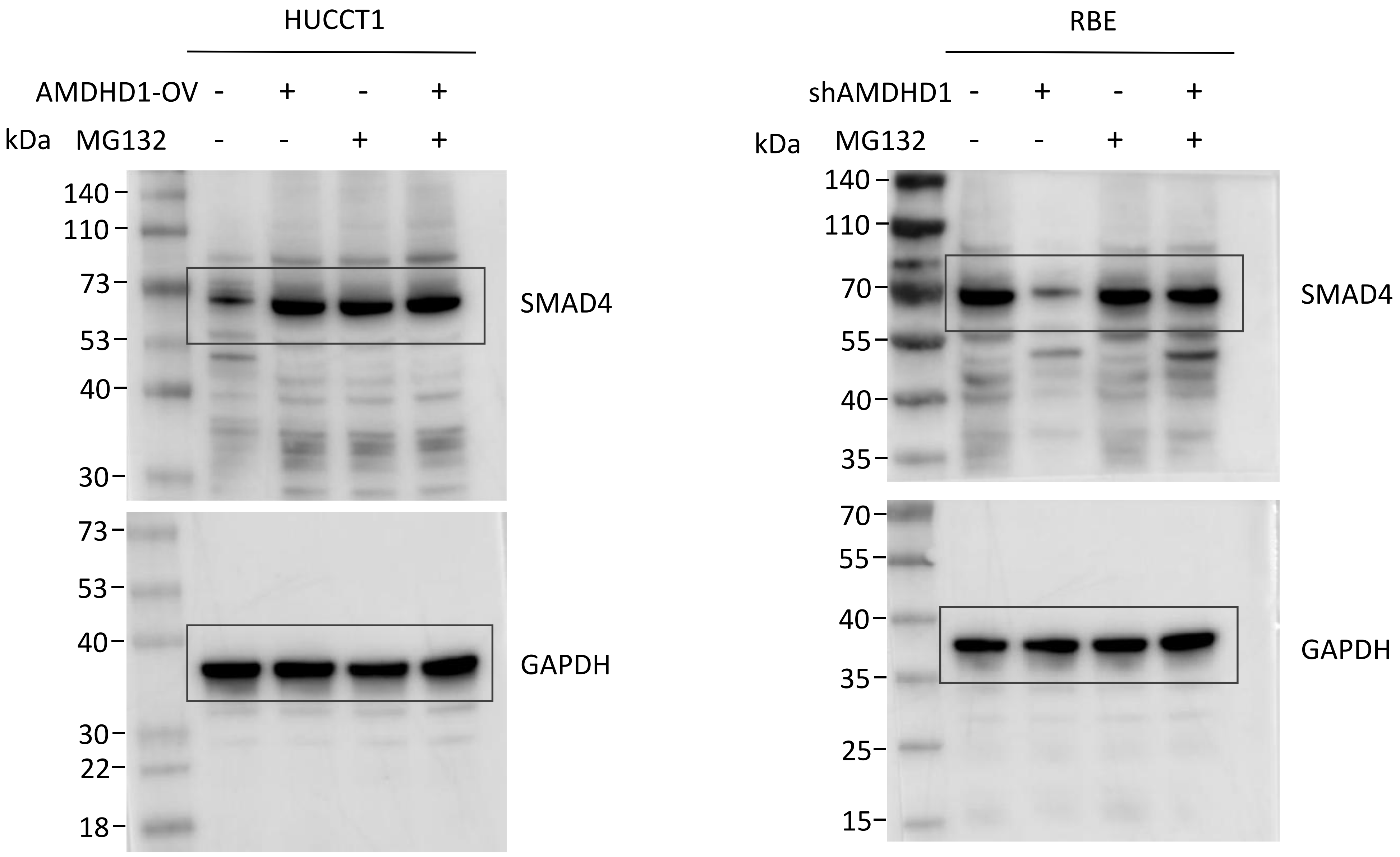
HUCCT1
AMDHD1-OV - + - +
kDa MG132 - - + +
140
110
73
53
40
30
SMAD4
73
53
40
30
22
18
GAPDH
RBE
shAMDHD1 - + - +
kDa MG132 - - + +
140
110
70
55
40
35
SMAD4
70
55
40
35
25
15
GAPDH

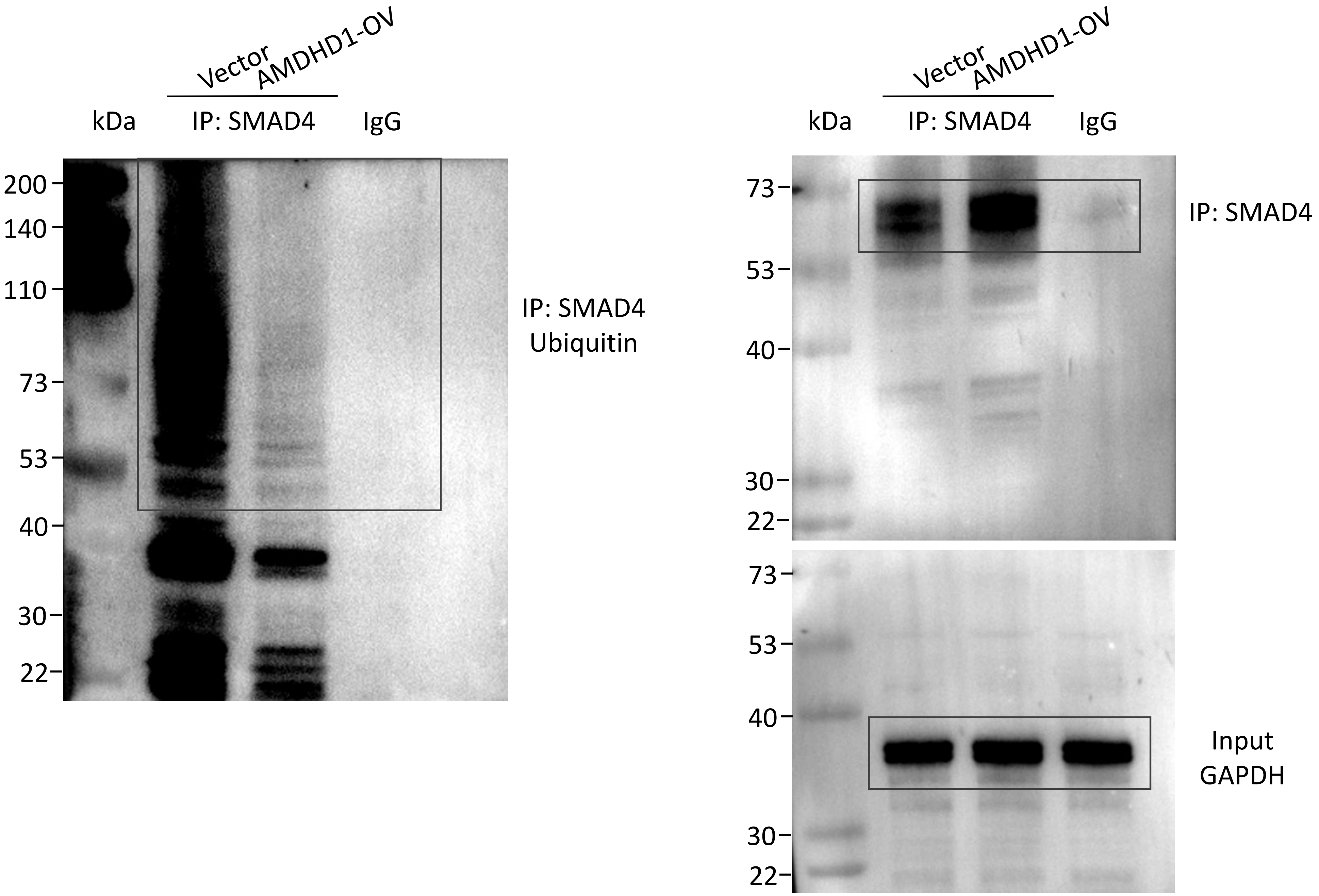
Vector
AMDHD1-OV
kDa
IP: SMAD4
IgG
200
140
110
73
53
40
30
22
IP: SMAD4
Ubiquitin
Vector
AMDHD1-OV
kDa
IP: SMAD4
IgG
73
53
40
30
22
IP: SMAD4
73
53
40
30
22
Input
GAPDH

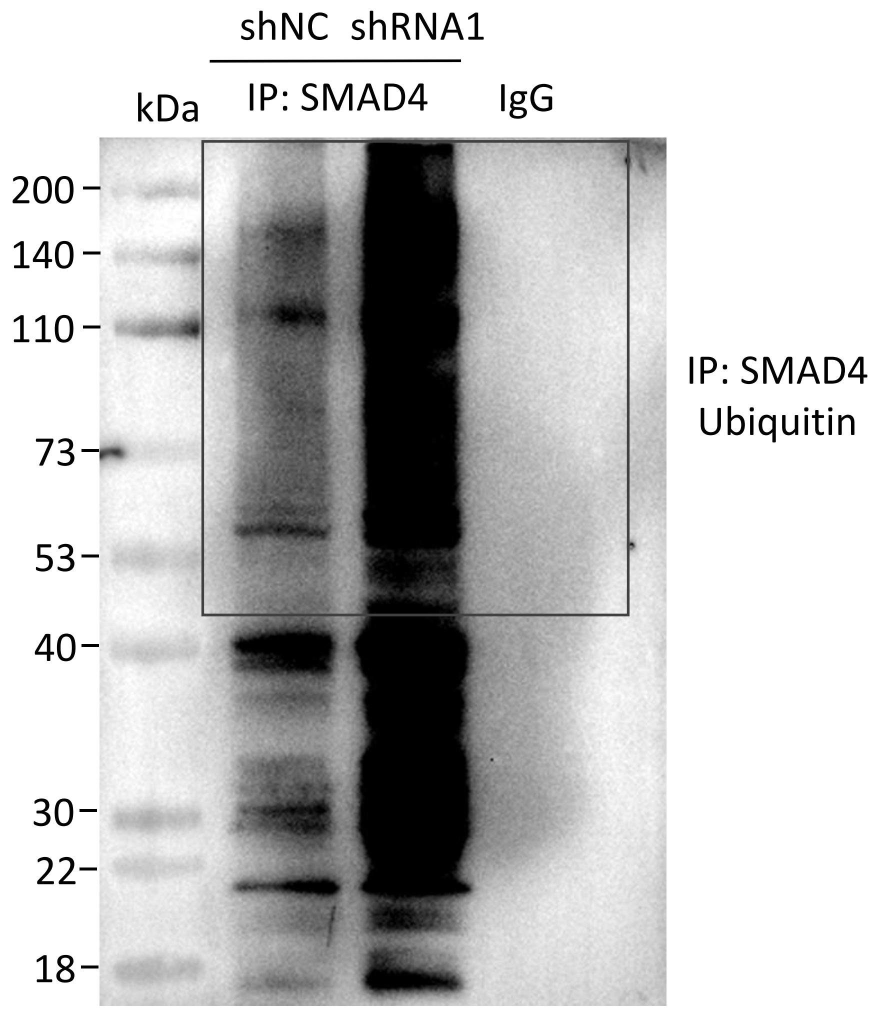
shNC shRNA1
IP: SMAD4
IgG
kDa
200
140
110
73
53
40
30
22
18
IP: SMAD4
Ubiquitin

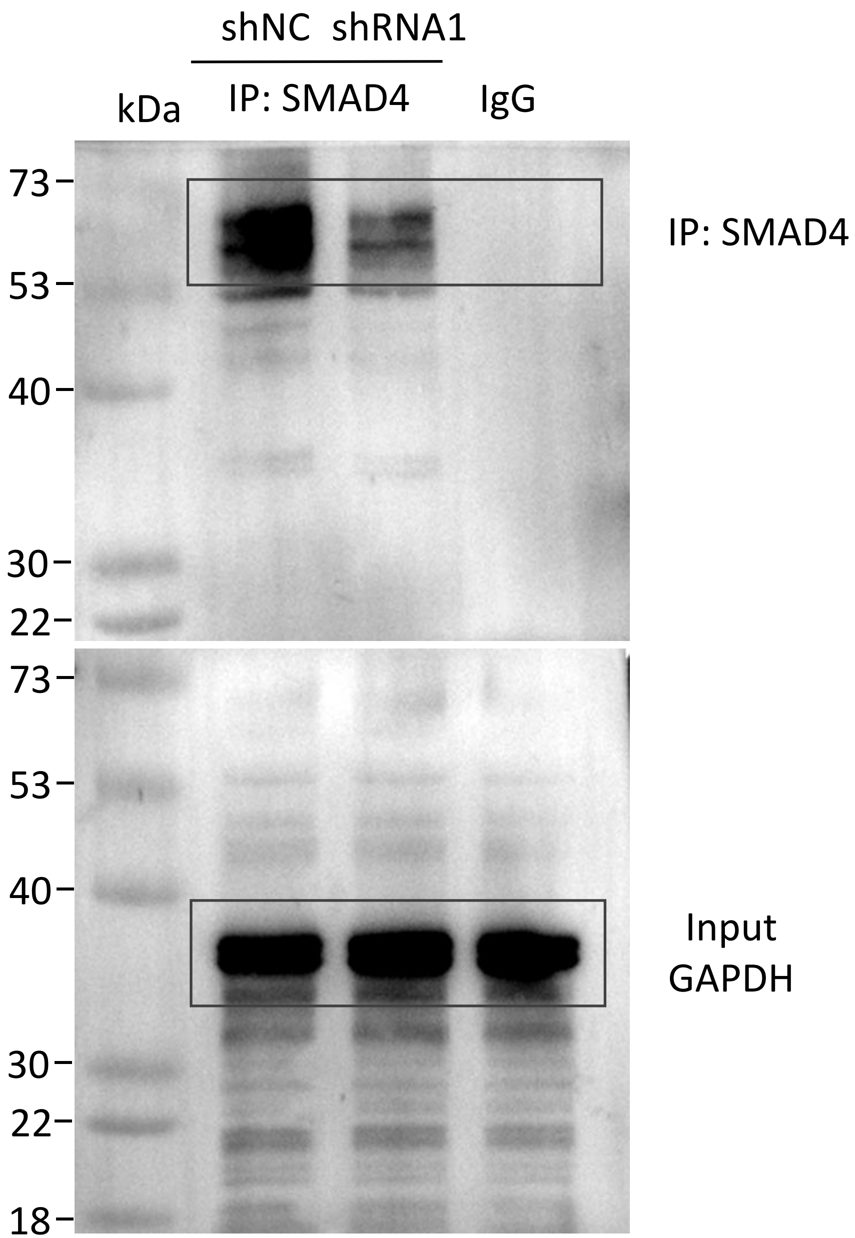
shNC shRNA1
IP: SMAD4
IgG
kDa
73
53
40
30
22
IP: SMAD4
73
53
40
30
22
18
Input
GAPDH

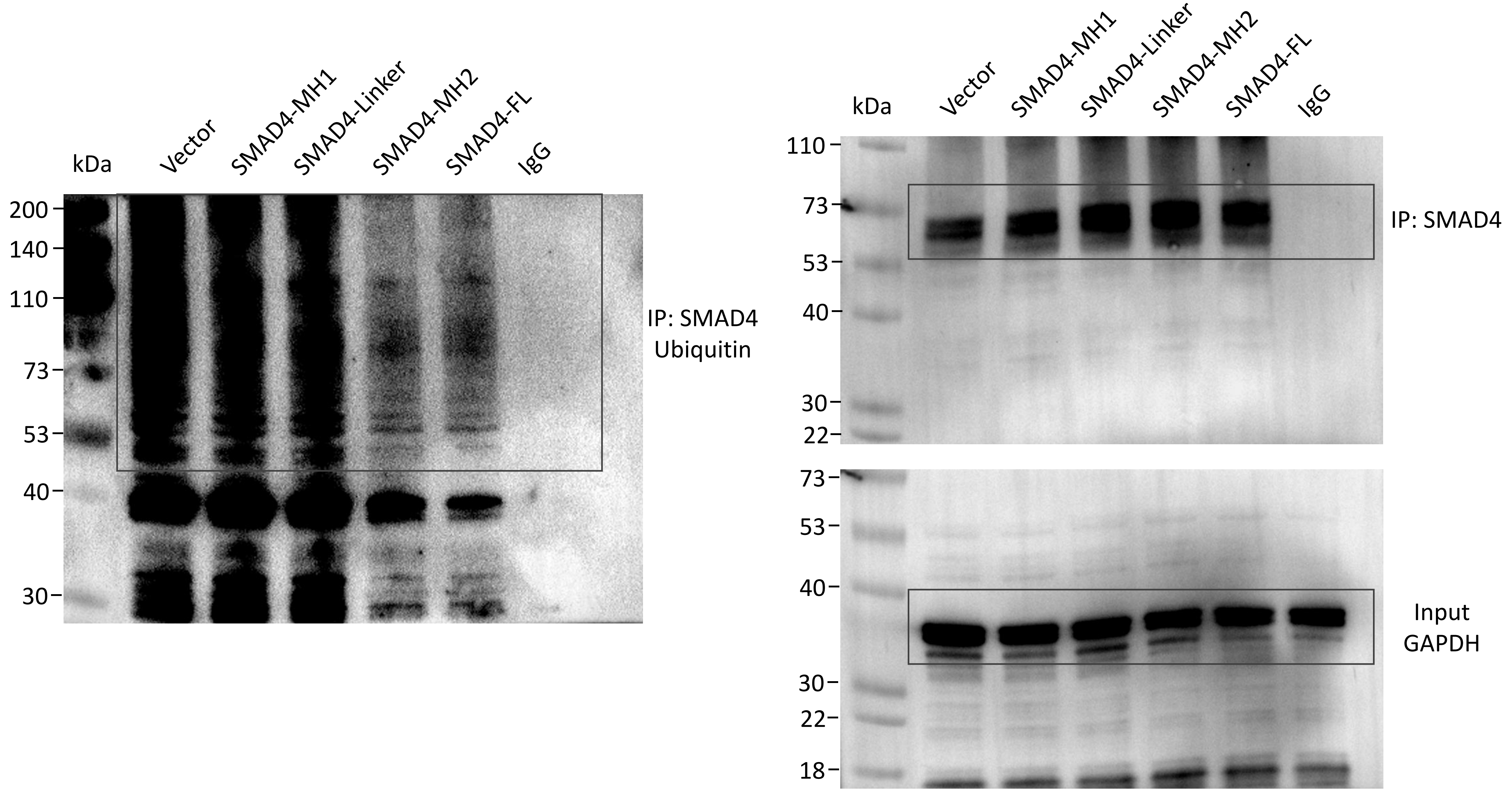
kDa
Vector
SMAD4-MH1
SMAD4-Linker
SMAD4-MH2
SMAD4-FL
IgG
200
140
110
73
53
40
30
IP: SMAD4
Ubiquitin
kDa
Vector
SMAD4-MH1
SMAD4-Linker
SMAD4-MH2
SMAD4-FL
IgG
110
73
53
40
30
22
IP: SMAD4
73
53
40
30
22
18
Input
GAPDH

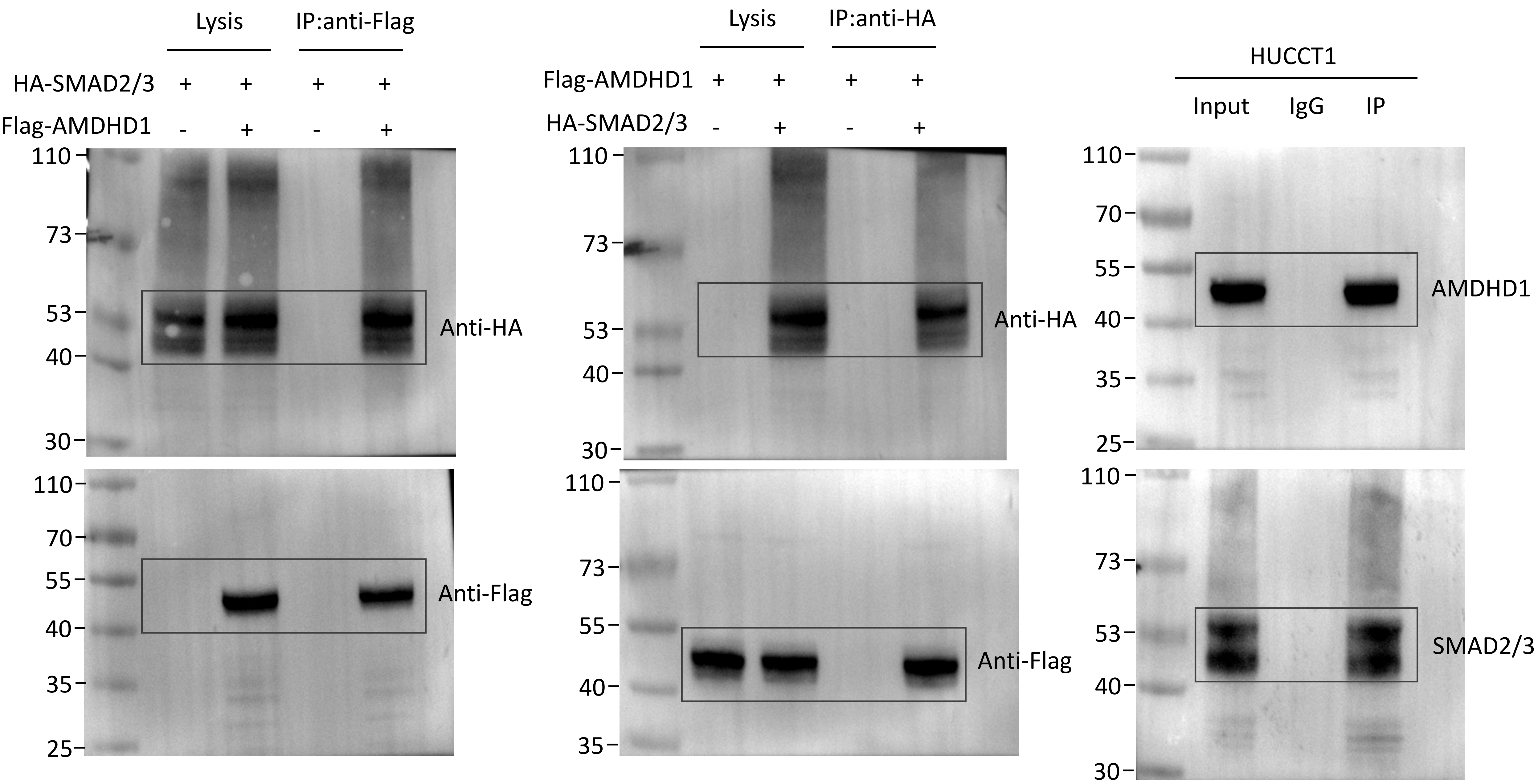
Lysis
IP:anti-Flag
HA-SMAD2/3 + + + +
Flag-AMDHD1 - + - +
110
73
53
40
30
Anti-HA
110
70
55
40
35
25
Anti-Flag
Lysis
IP:anti-HA
Flag-AMDHD1 + + + +
HA-SMAD2/3 - + - +
110
73
53
40
30
Anti-HA
110
73
55
40
35
Anti-Flag
HUCCT1
Input
IgG
IP
110
70
55
40
35
25
AMDHD1
110
73
53
40
30
SMAD2/3

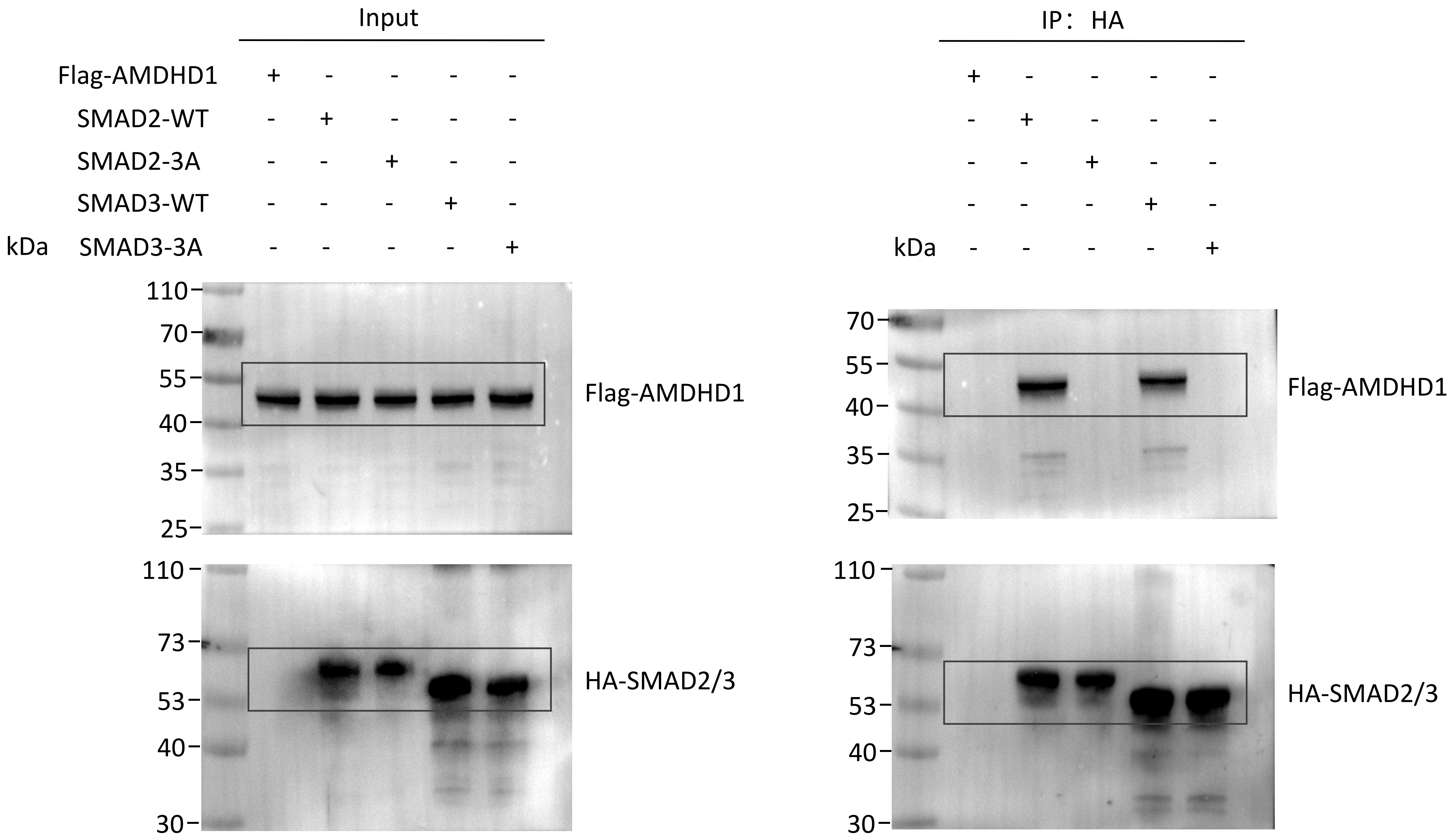
Input
IP: HA
Flag-AMDHD1
SMAD2-WT
SMAD2-3A
SMAD3-WT
SMAD3-3A
kDa
110
70
55
40
35
25
73
53
30
Flag-AMDHD1
HA-SMAD2/3

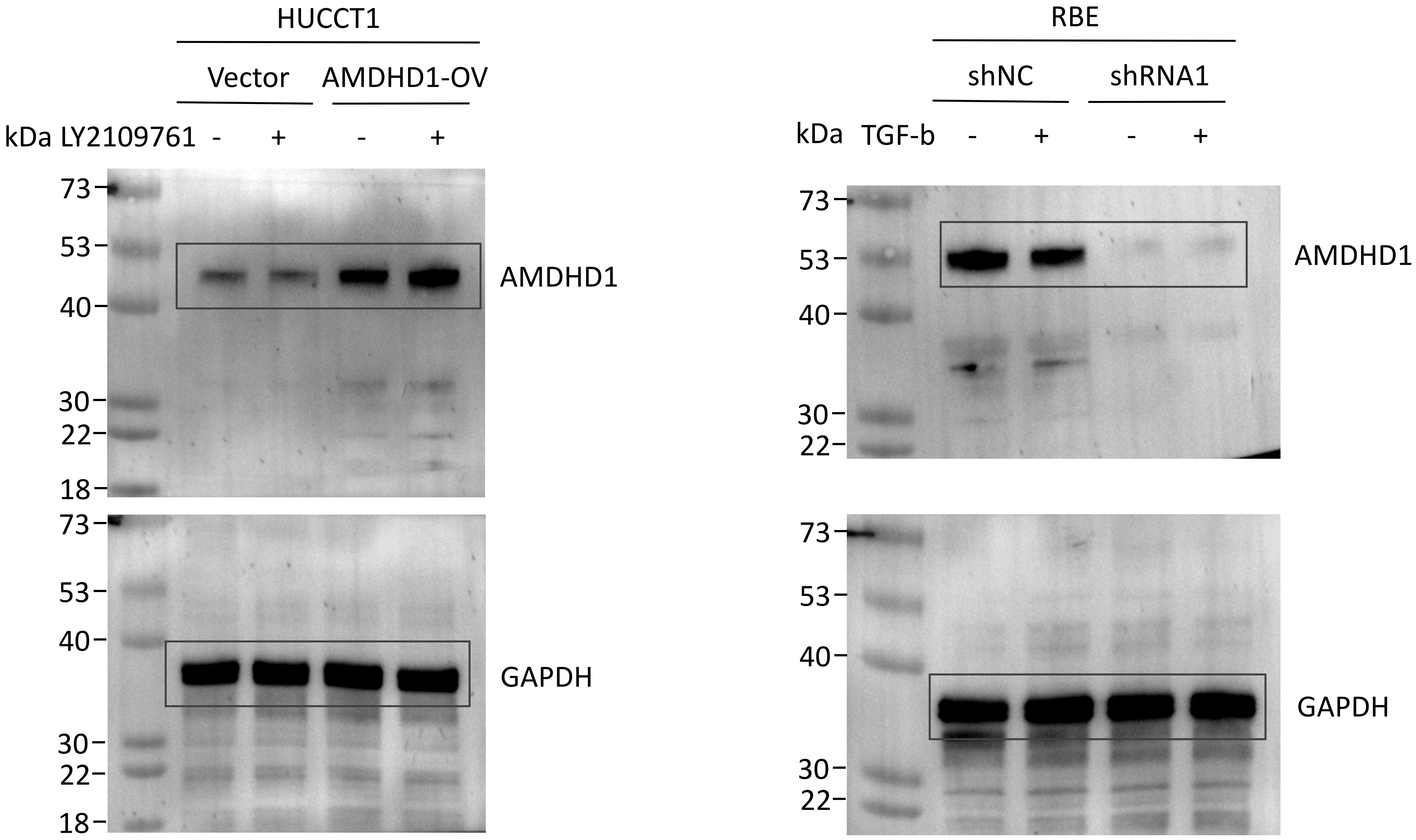
HUCCT1
Vector
AMDHD1-OV
kDa LY2109761 - + - +
73
53
40
30
22
18
AMDHD1
GAPDH
RBE
shNC
shRNA1
kDa TGF-b - + - +
AMDHD1
GAPDH

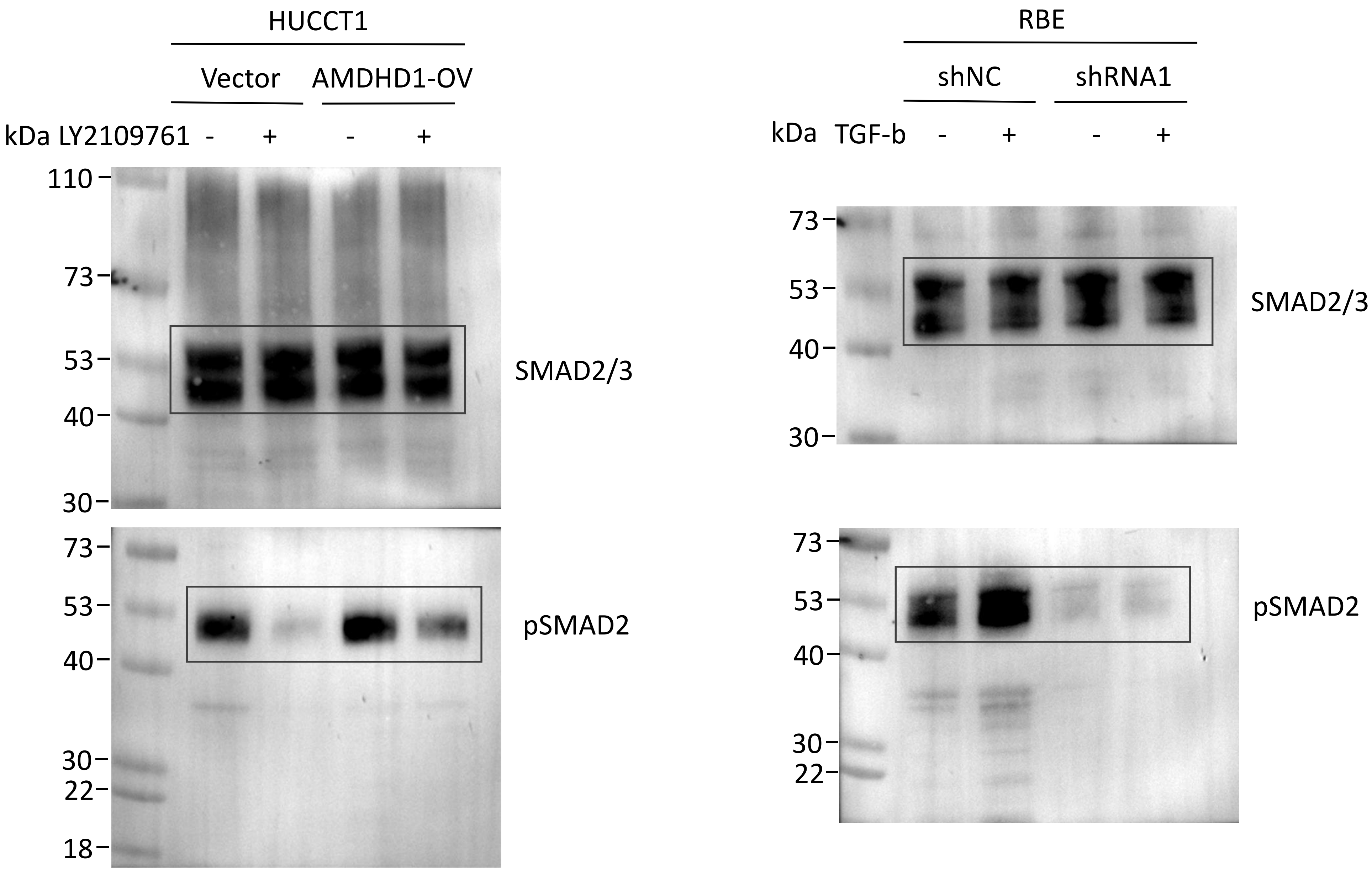
HUCCT1
Vector
AMDHD1-OV
kDa LY2109761 - + - +
110
73
53
40
30
SMAD2/3
73
53
40
30
22
18
pSMAD2
RBE
shNC
shRNA1
kDa TGF-b - + - +
73
53
40
30
SMAD2/3
73
53
40
30
22
pSMAD2

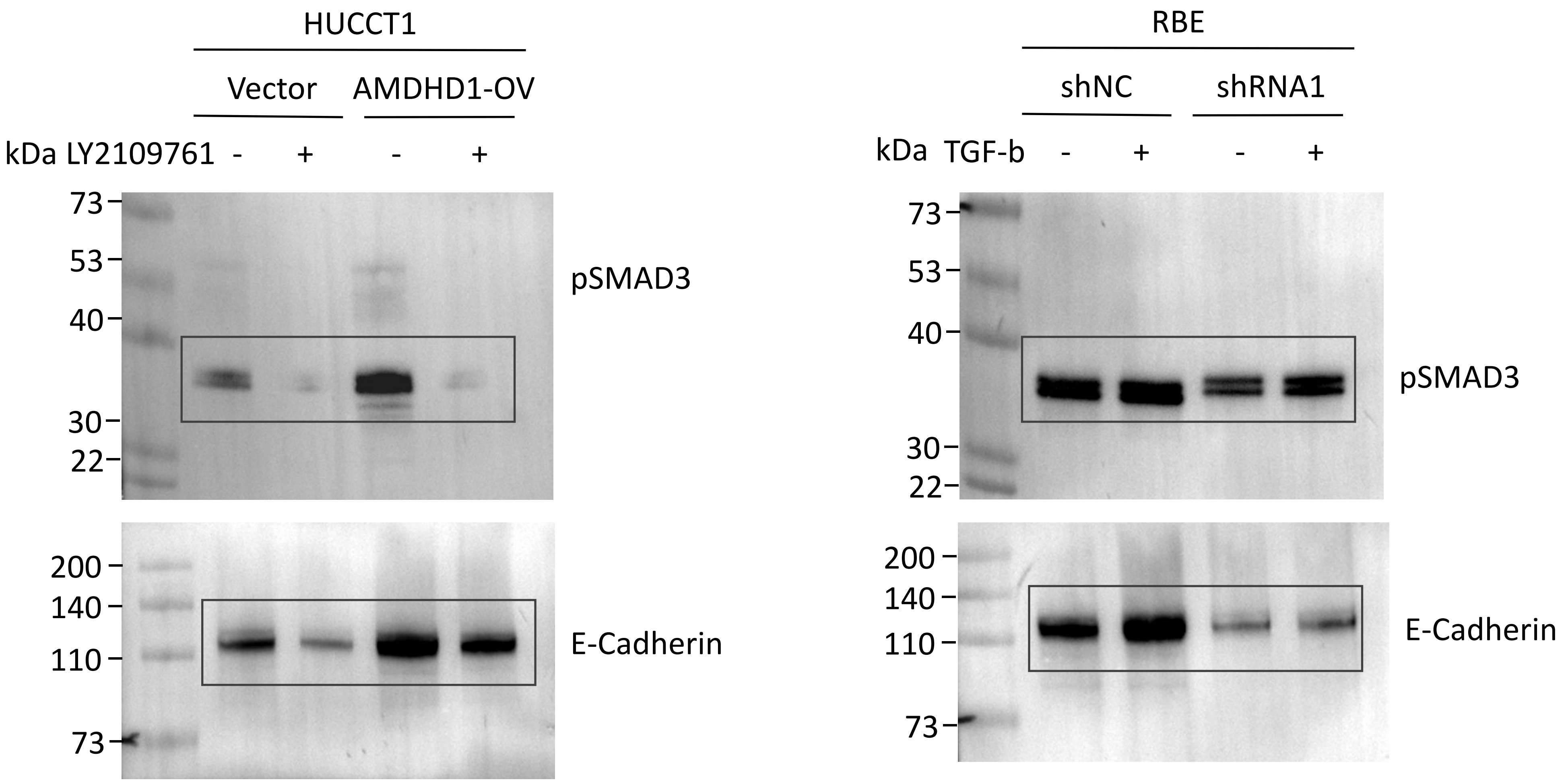
HUCCT1
Vector
AMDHD1-OV
kDa LY2109761 - + - +
73
53
40
30
22
pSMAD3
200
140
110
73
E-Cadherin
RBE
shNC
shRNA1
kDa TGF-b - + - +
73
53
40
30
22
pSMAD3
200
140
110
73
E-Cadherin

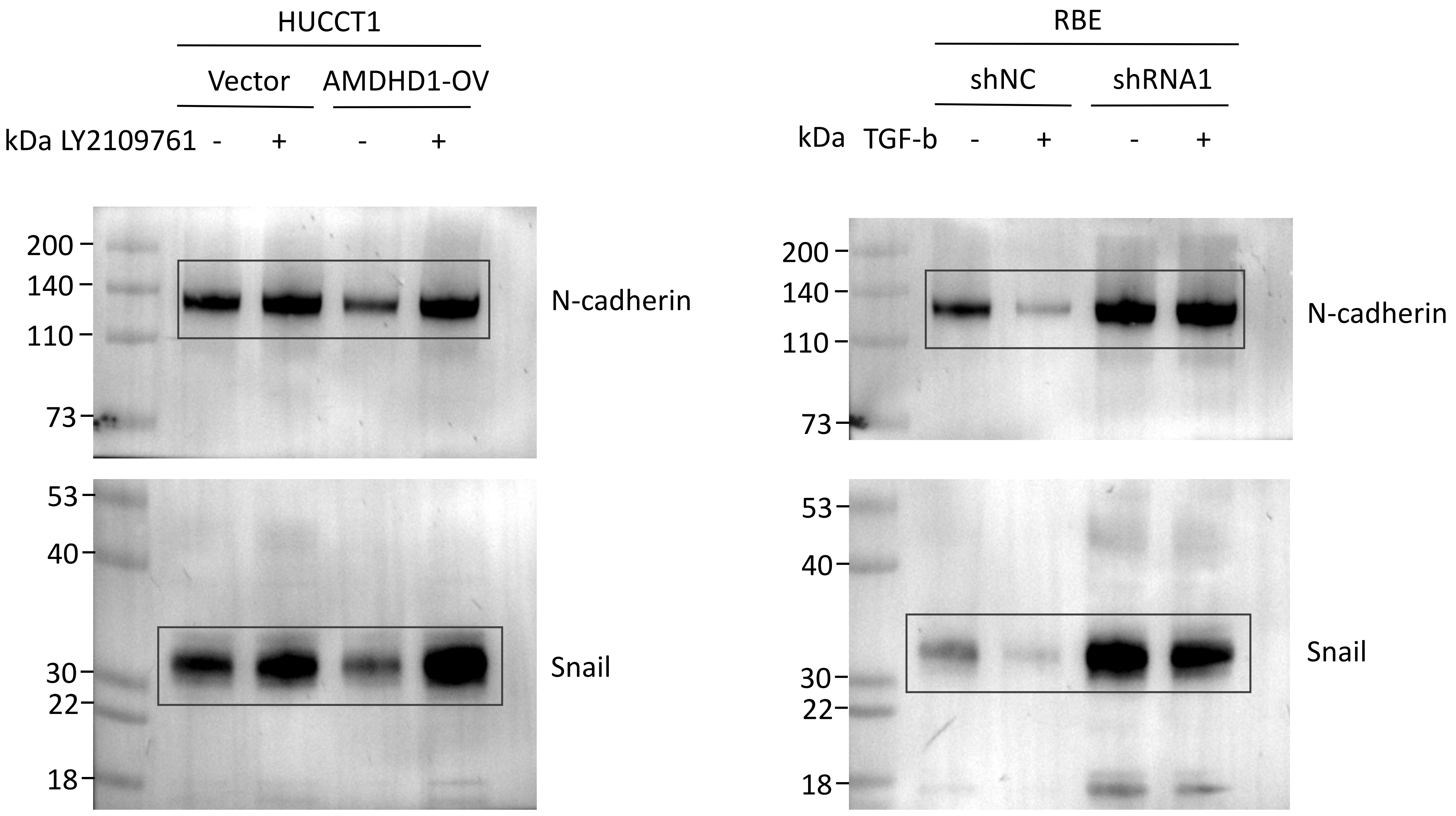
HUCCT1
Vector
AMDHD1-OV
kDa LY2109761 - + - +
200
140
110
73
N-cadherin
53
40
30
22
18
Snail
RBE
shNC
shRNA1
kDa TGF-b - + - +
200
140
110
73
N-cadherin
53
40
30
22
18
Snail

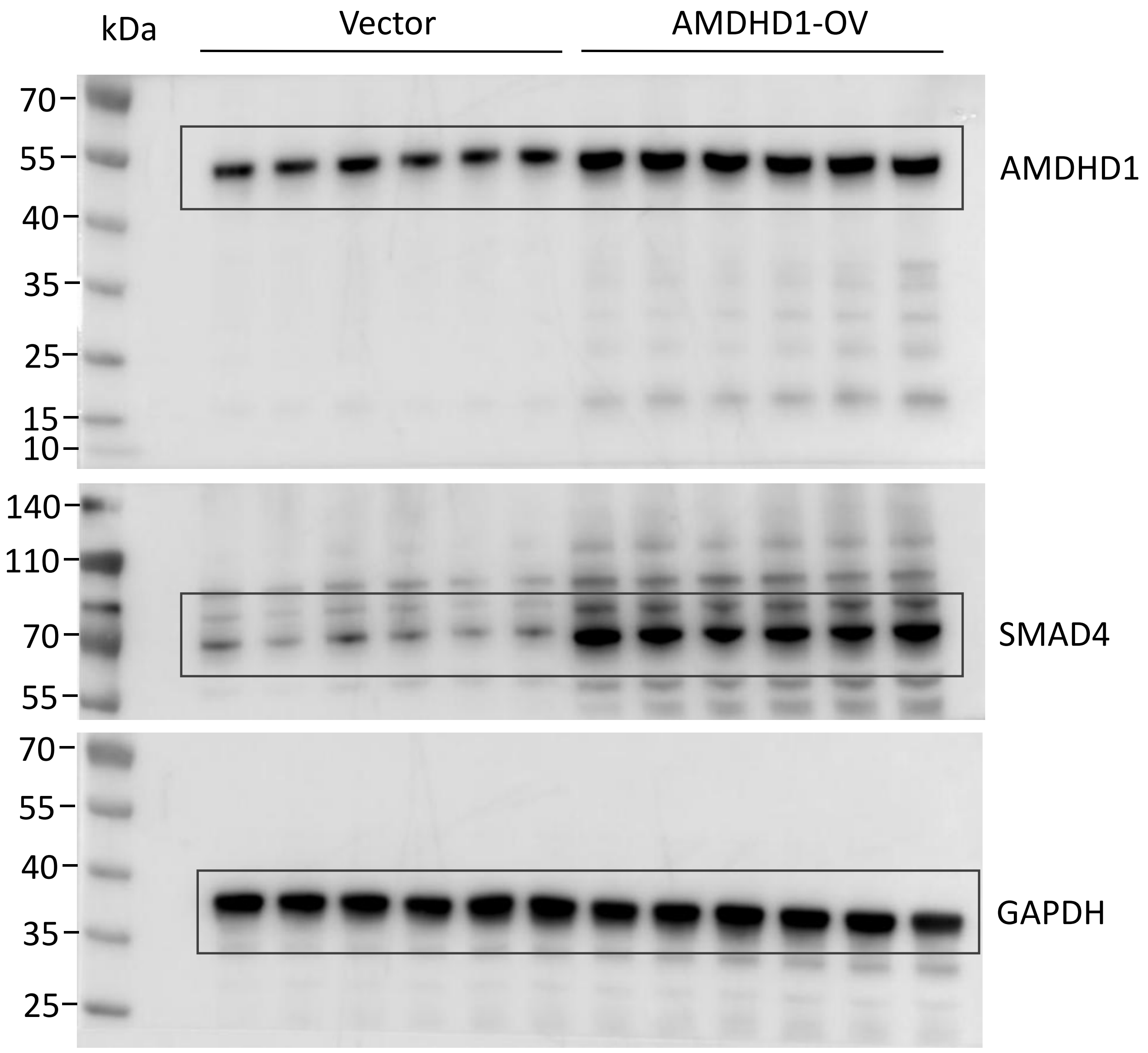
kDa
Vector
AMDHD1-OV
70
55
40
35
25
15
10
AMDHD1
140
110
70
55
SMAD4
70
55
40
35
25
GAPDH

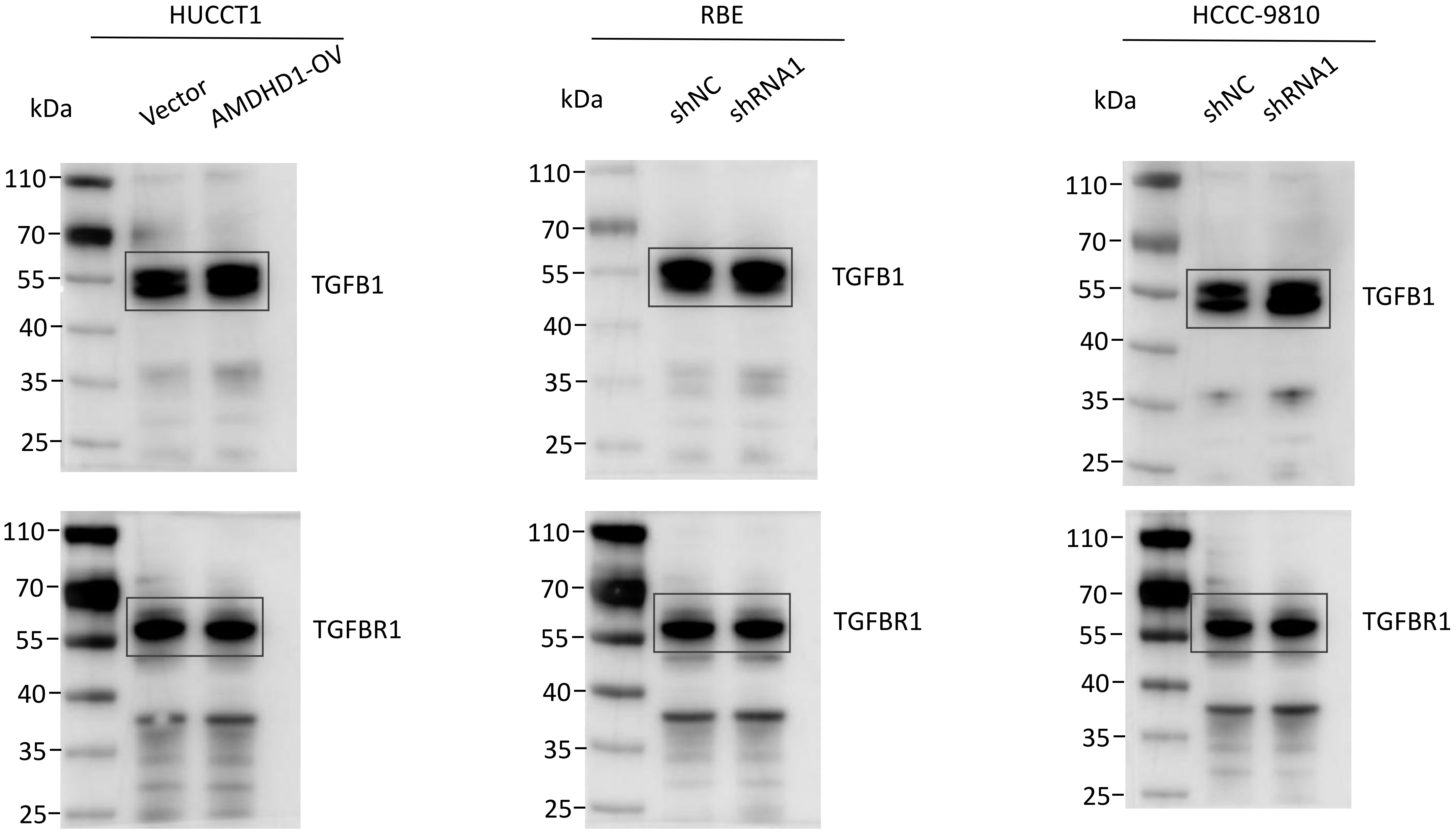

HUCCT1
kDa
Vector
AMDHD1-OV
RBE
shNC
shRNA1
HCCC-9810
110
70
55
40
35
25
TGFB1
TGFBR1

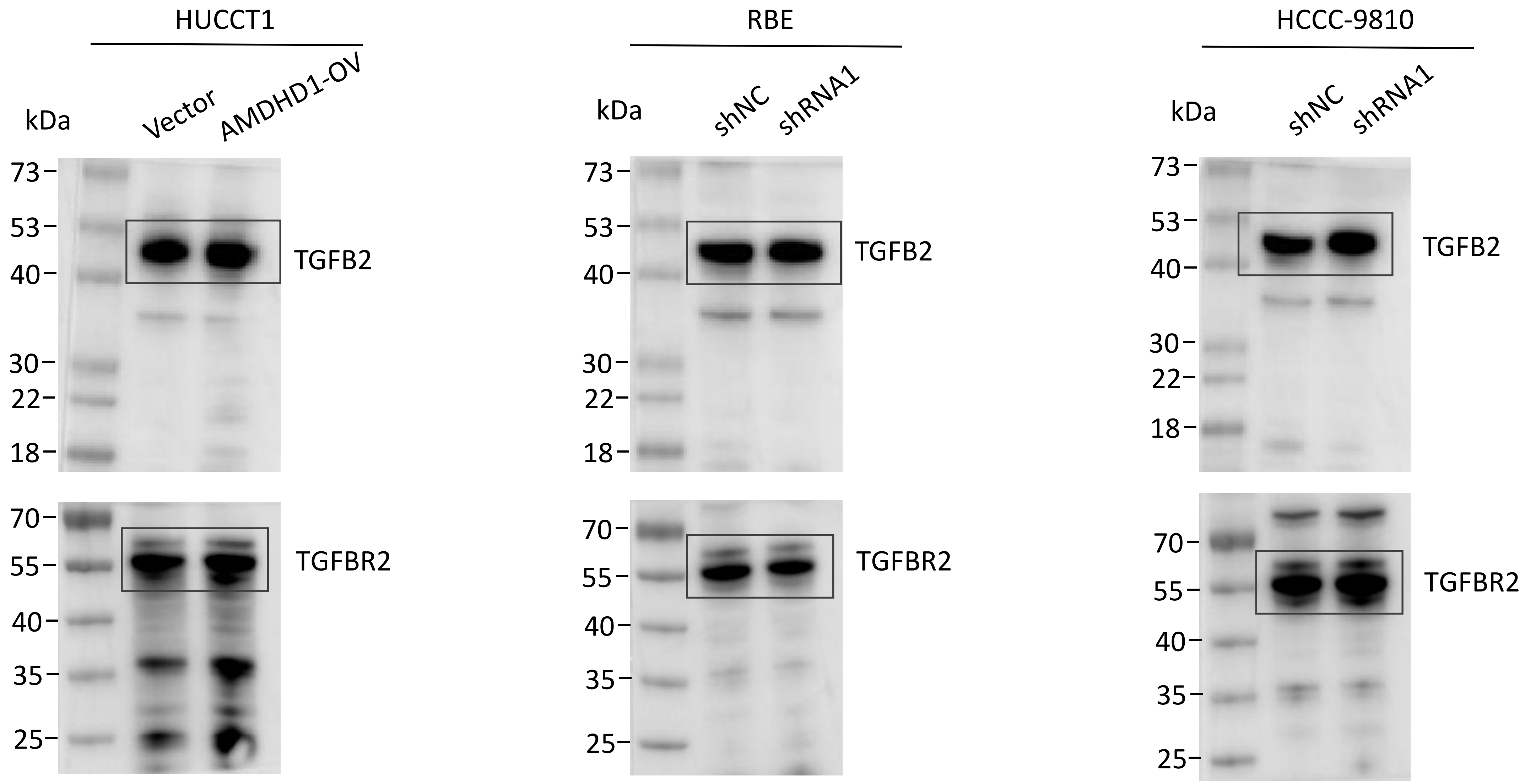
HUCCT1
kDa
Vector
AMDHD1-OV
73
53
40
30
22
18
TGFB2
70
55
40
35
25
TGFBR2
RBE
kDa
shNC
shRNA1
73
53
40
30
22
18
TGFB2
70
55
40
35
25
TGFBR2
HCCC-9810
kDa
shNC
shRNA1
73
53
40
30
22
18
TGFB2
70
55
40
35
25
TGFBR2

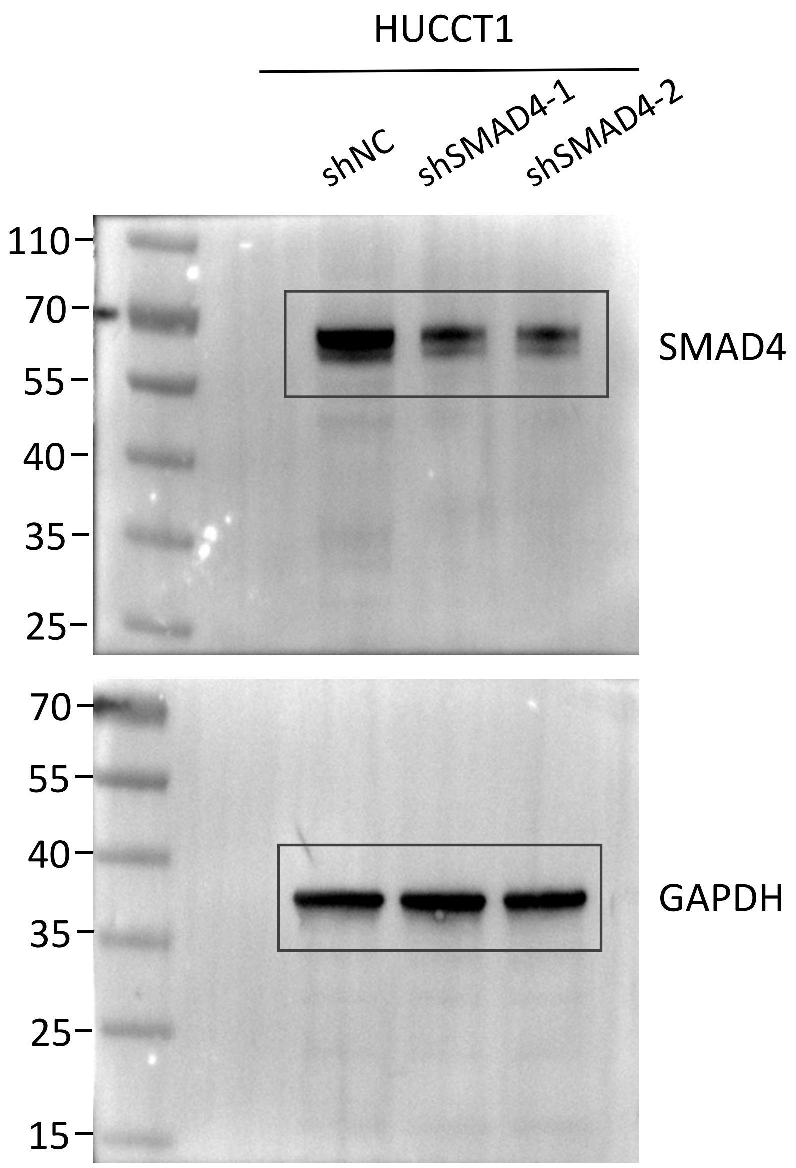

HUCCT1
shNC
shSMAD4-1
shSMAD4-2
110
70
55
40
35
25
SMAD4
70
55
40
35
25
15
GAPDH

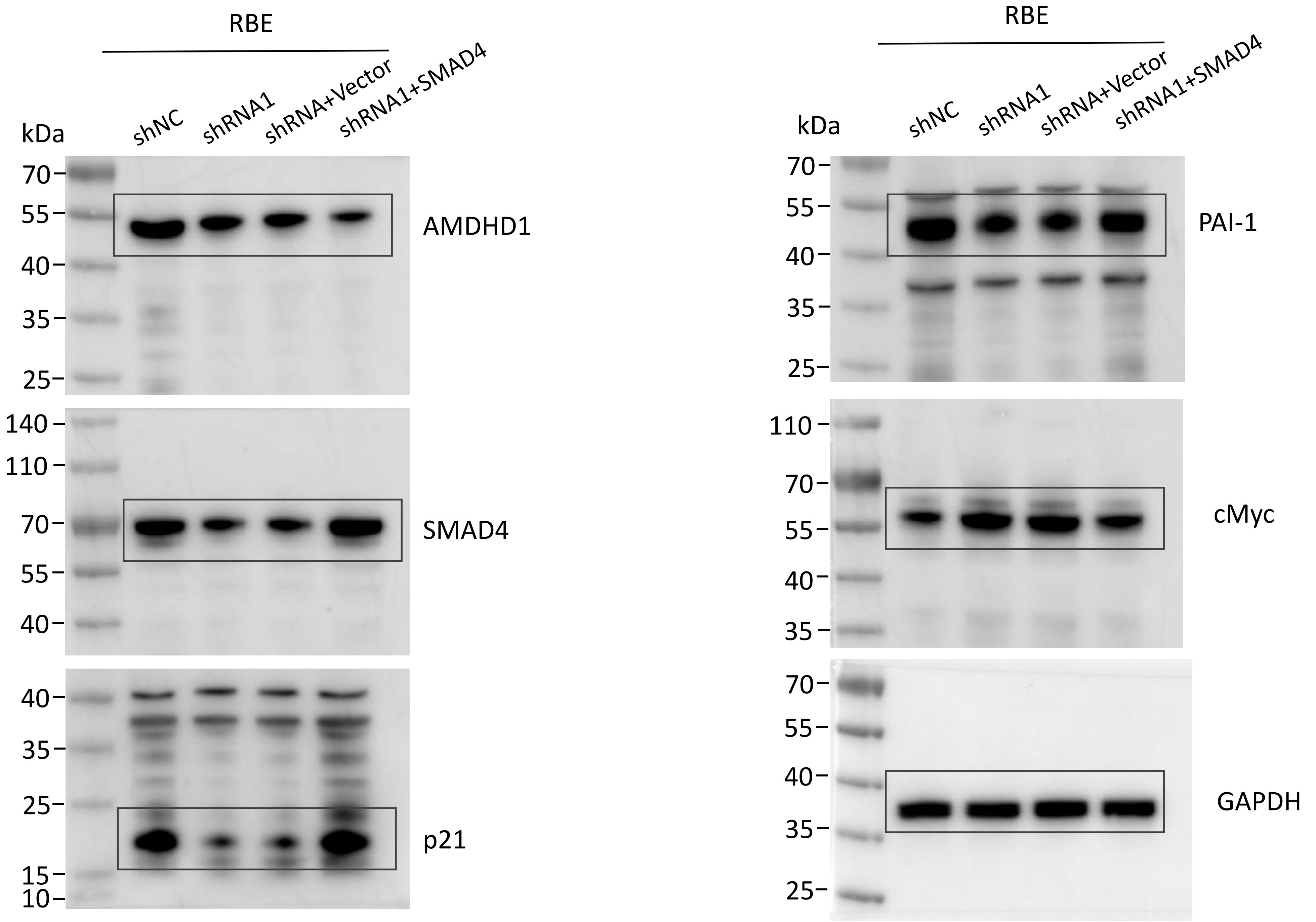

RBE
shNC
shRNA1
shRNA+Vector
shRNA1+SMAD4
kDa
70
55
40
35
25
AMDHD1
140
110
70
55
40
SMAD4
40
35
25
15
10
p21
RBE
shNC
shRNA1
shRNA+Vector
shRNA1+SMAD4
kDa
70
55
40
35
25
PAI-1
110
70
55
40
35
cMyc
70
55
40
35
25
GAPDH

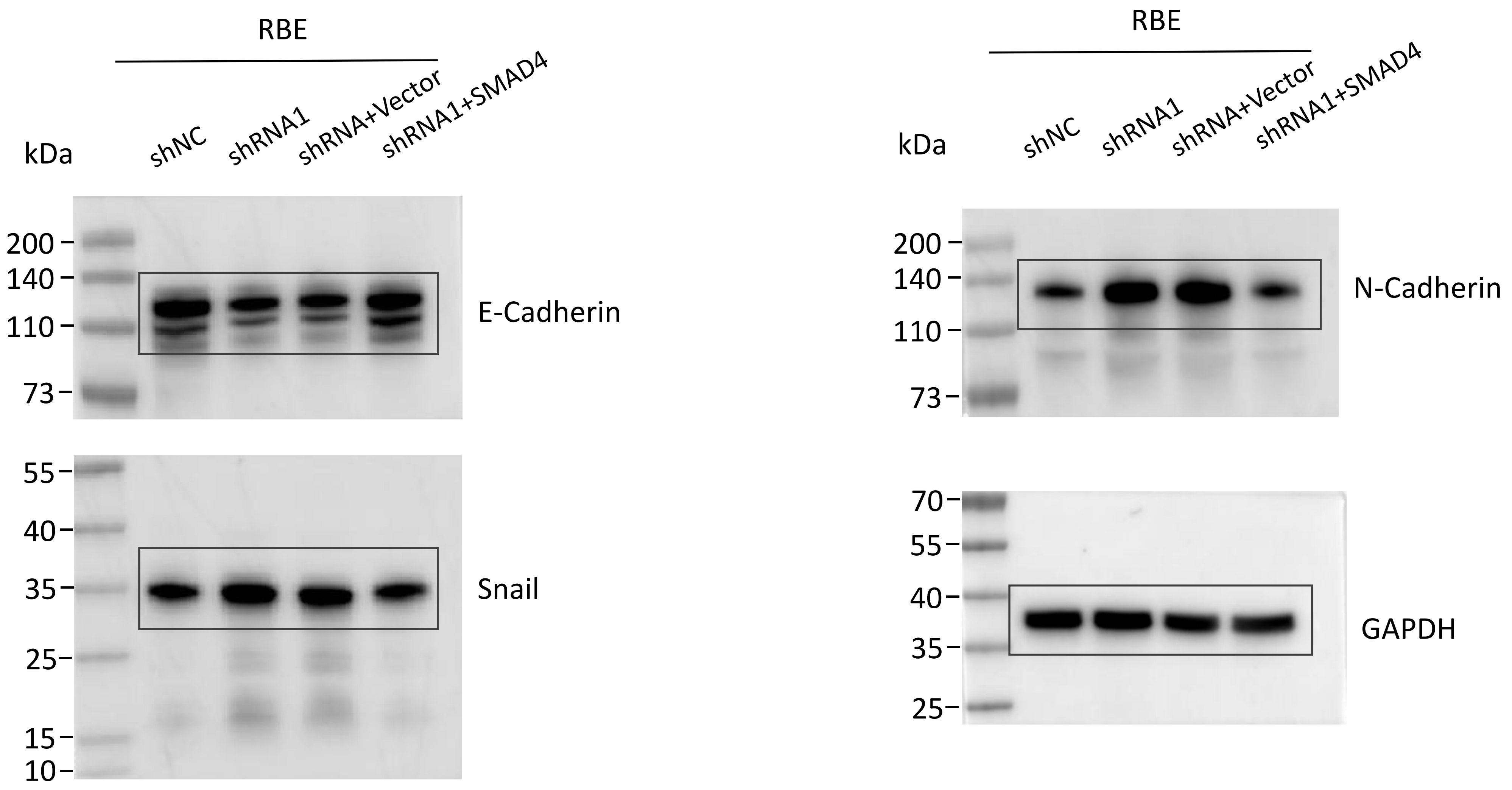
RBE
shNC
shRNA1
shRNA+Vector
shRNA1+SMAD4
kDa
200
140
110
73
E-Cadherin
55
40
35
25
15
10
Snail
RBE
shNC
shRNA1
shRNA+Vector
shRNA1+SMAD4
kDa
200
140
110
73
N-Cadherin
70
55
40
35
25
GAPDH

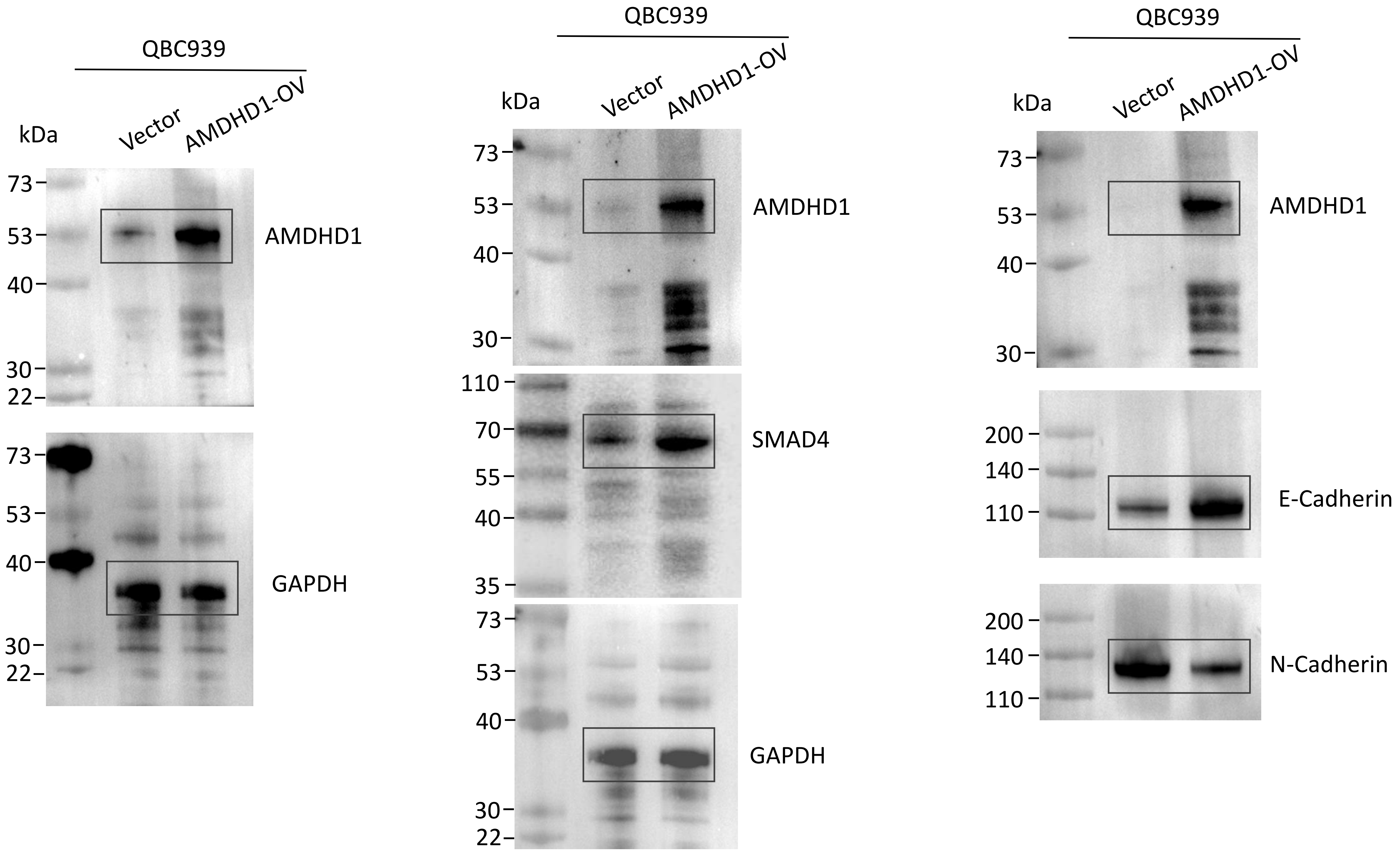
QBC939
kDa
Vector
AMDHD1-OV
73
53
40
30
22
AMDHD1
GAPDH
110
70
55
35
SMAD4
200
140
E-Cadherin
N-Cadherin

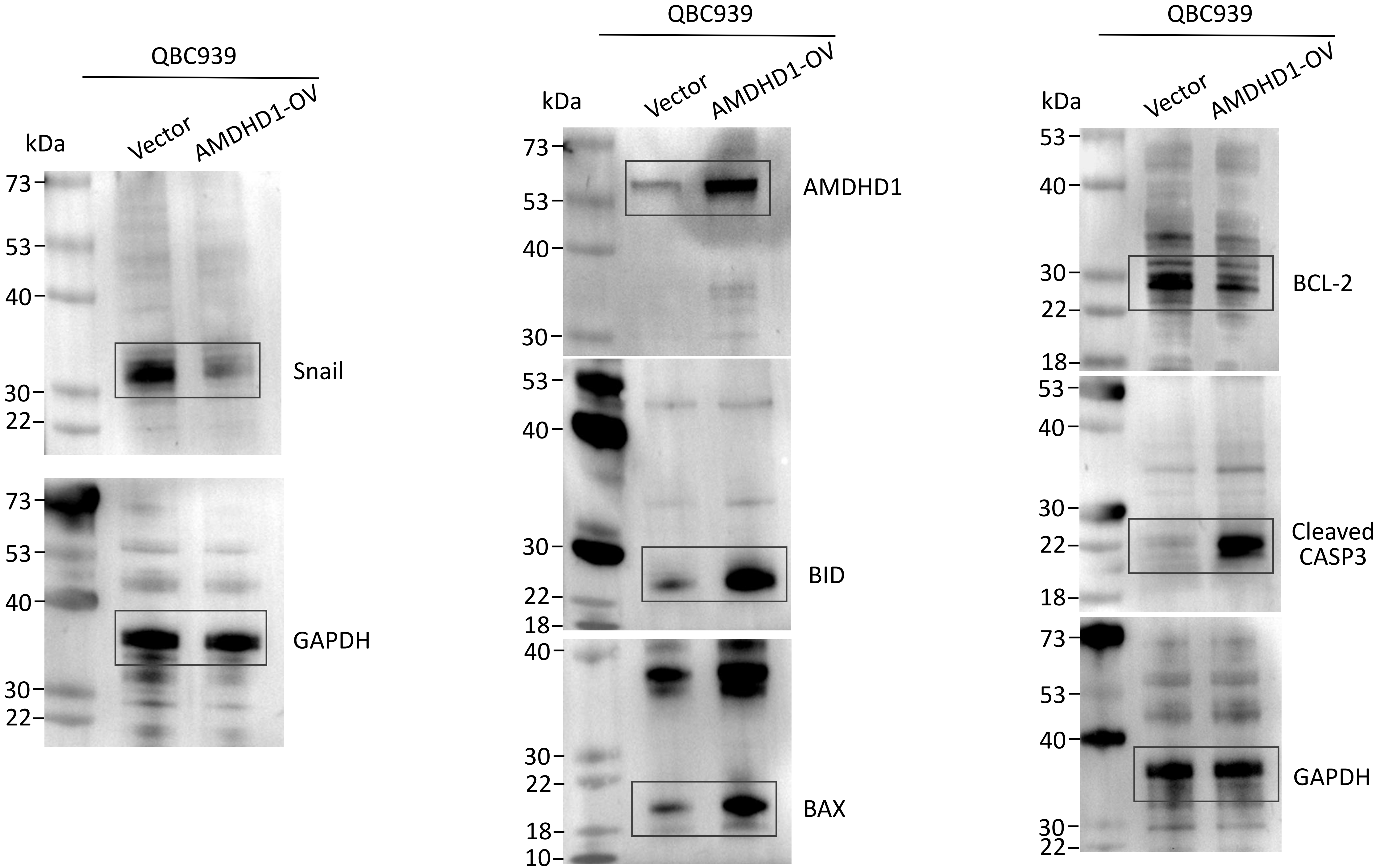

QBC939
kDa
Vector
AMDHD1-OV
73
53
40
30
22
Snail
GAPDH
QBC939
kDa
Vector
AMDHD1-OV
73
53
40
30
AMDHD1
BID
18
BAX
10
QBC939
kDa
Vector
AMDHD1-OV
BCL-2
Cleaved CASP3
GAPDH

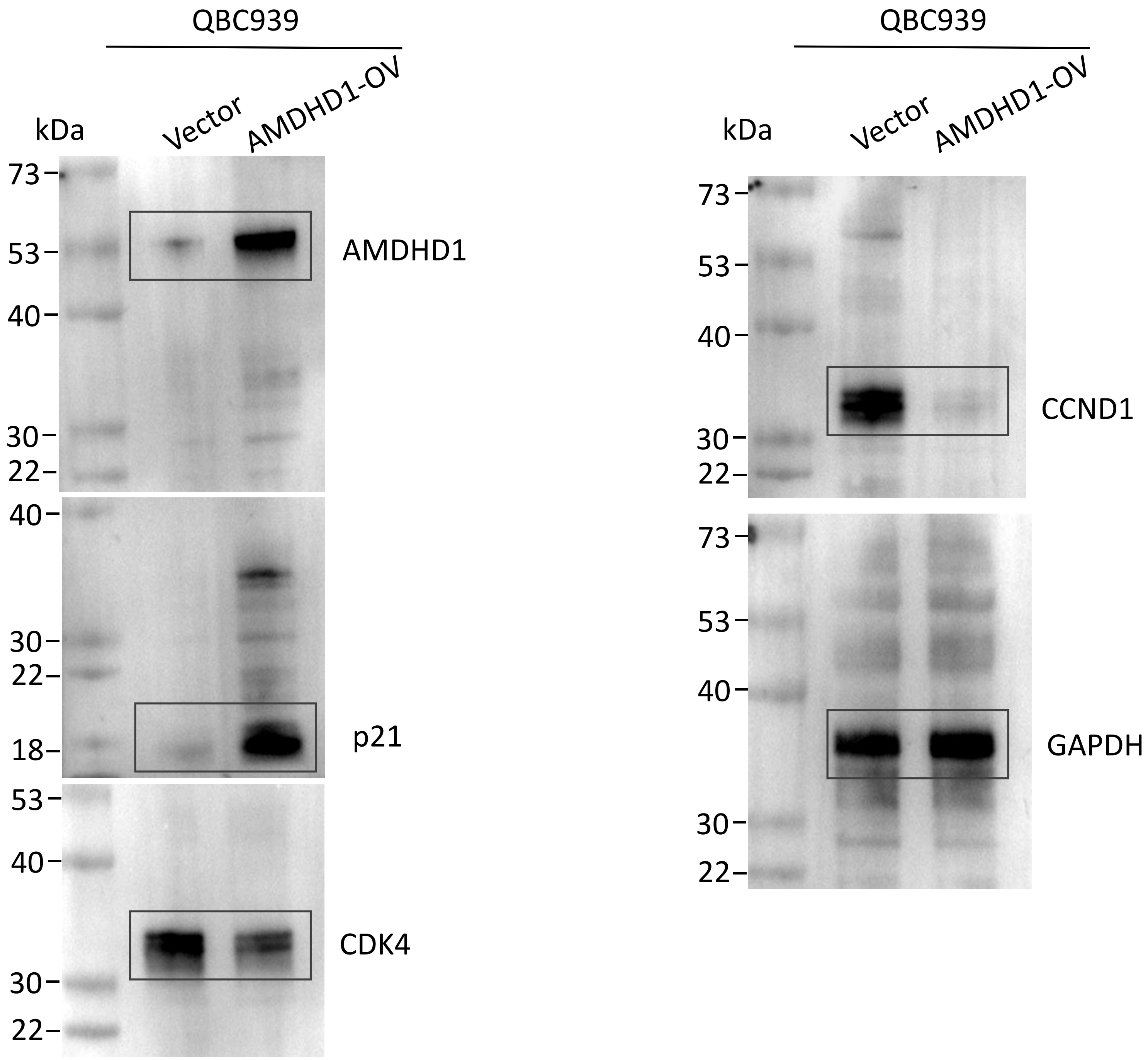
QBC939
kDa
Vector
AMDHD1-OV
73
53
40
30
22
AMDHD1
40
30
22
18
p21
53
40
30
22
CDK4
QBC939
kDa
Vector
AMDHD1-OV
73
53
40
30
22
CCND1
73
53
40
30
22
GAPDH
